# Novel hybrids of 1,2,3-triazole-benzoxazole: design, synthesis, and assessment of DprE1 enzyme inhibitors using fluorometric assay and computational analysis

**DOI:** 10.1080/14756366.2024.2403744

**Published:** 2024-09-27

**Authors:** Manisha Singh, Sarah M. Batt, Christian S. C. Canales, Fernando R. Pavan, Sethu Arun Kumar, Handattu S. Akshatha, Meduri Bhagyalalitha, Karthik G. Pujar, Durgesh Bidye, Gurubasavaraj V. Pujar, Gurdyal S. Besra

**Affiliations:** a Computer Aided Drug Design Lab, Department of Pharmaceutical Chemistry, JSS College of Pharmacy, JSS Academy of Higher Education and Research, Sri Shivarathreeshwara Nagara, Mysore, India; b School of Biosciences, University of Birmingham, Birmingham, United Kingdom; c Faculty of Pharmaceutical Sciences, Paulista State University—UNESP, Araraquara, SP, Brazil

**Keywords:** Tuberculosis, 1,2,3-triazoles, DprE1 inhibitor assay

## Abstract

Decaprenylphosphoryl-β-D-ribose-oxidase (DprE1), a subunit of the essential decaprenylphosphoribose-2′-epimerase, plays a crucial role in the synthesis of cell wall arabinan components in mycobacteria, including the pathogen responsible for tuberculosis, *Mycobacterium tuberculosis*. In this study, we designed, synthesised, and evaluated 15 (BOK-1–BOK-10 and BOP-1–BOP-5) potential inhibitors of DprE1 from a series of 1,2,3-triazole ligands using a validated DprE1 inhibition assay. Two compounds, BOK-2 and BOK-3, demonstrated significant inhibition with IC_50_ values of 2.2 ± 0.1 and 3.0 ± 0.6 μM, respectively, whereas the standard drug (TCA-1) showed inhibition at 3.0 ± 0.2 μM. Through molecular modelling and dynamic simulations, we explored the structural relationships between selected 1,2,3-triazole compounds and DprE1, revealing key features for effective drug–target interactions. This study introduces a novel approach for designing ligands against DprE1, offering a potential therapeutic strategy for tuberculosis treatment.

## HIGHLIGHTS

- Identification of 15 (BOK-1–BOK-10 and BOP-1–BOP-5) potent inhibitors of DprE1 enzyme from 1,2,3-triazole ligands.
- BOK-2 and BOK-3 exhibited significant DprE1 inhibition with IC_50_ values of 2.2 ± 0.1 and 3.0 ± 0.6 μM, respectively.
- Molecular modelling and dynamic simulations elucidated key structural features for effective drug–target interactions.
- Novel approach introduced for designing DprE1 ligands, potentially aiding tuberculosis treatment.
- Findings offer promising candidates for future tuberculosis research.

## Graphical abstract

**Figure:**
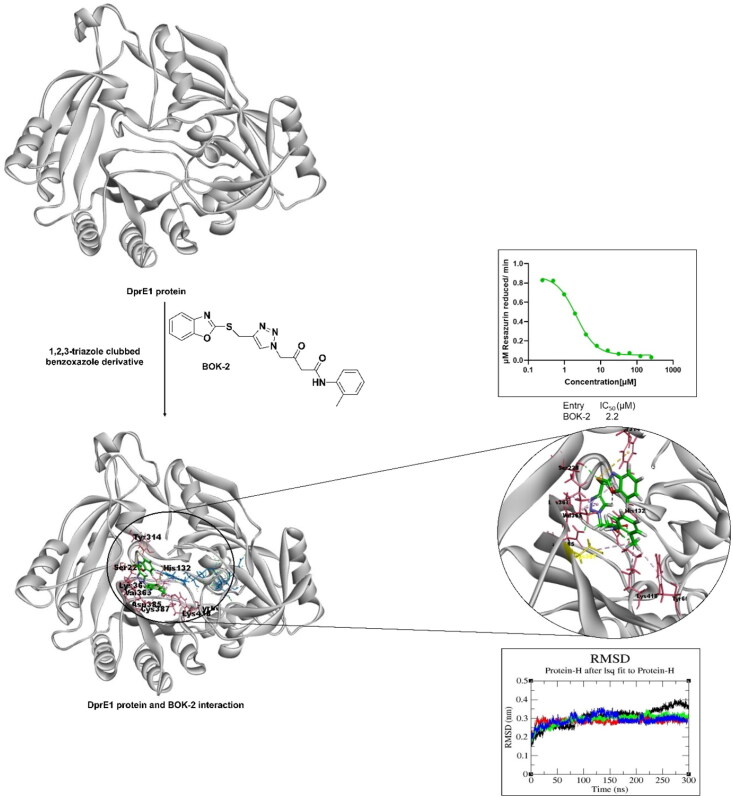

## Introduction

Tuberculosis (TB) continues to be a significant global health issue, with the emergence of drug-resistant TB (DR-TB) presenting a considerable challenge^1^. The increasing prevalence of DR-TB can complicate TB treatment, leading to higher failure rates, extended therapy durations, and more complex medication regimens^1^^,^^2^. Currently, TB treatment involves different therapeutic approaches, which can increase to 2 years for DR-TB. This lengthy treatment, along with the associated side effects, often results in patients prematurely discontinuing their treatment, exacerbating the issue of drug resistance^2^. Furthermore, the effect of the Coronavirus Disease - 2019 (COVID-19) pandemic has the potential to disrupt the prompt identification and treatment of newly diagnosed TB cases, adding to the burden of TB^3^.

In the fight against TB, a major obstacle is the limited availability of newer and more effective drugs. Since rifampicin gained approval in the 1960s, the FDA has sanctioned only two additional anti-TB drugs, namely pretomanid and bedaquiline^4^. Despite ongoing efforts, there is a pressing need to discover and develop newer and more efficacious anti-TB drugs. Among many approaches, treating tuberculosis could be achieved by inhibiting the mycobacterium cell wall synthesis^5^. Primary drugs like ethambutol and isoniazid inhibit key enzymes of *Mycobacterium tuberculosis* (Mtb) involved in the synthesis of the arabinogalactan and mycolic acid layers, which are essential to protect the cell from antibiotics and the host’s defences^6^. Recent advancements in whole-cell screening have led to the discovery of new drug families that specifically interact with crucial target proteins involved in the formation of structural elements of the cell wall^6^^,^^7^.

**Figure 1.**
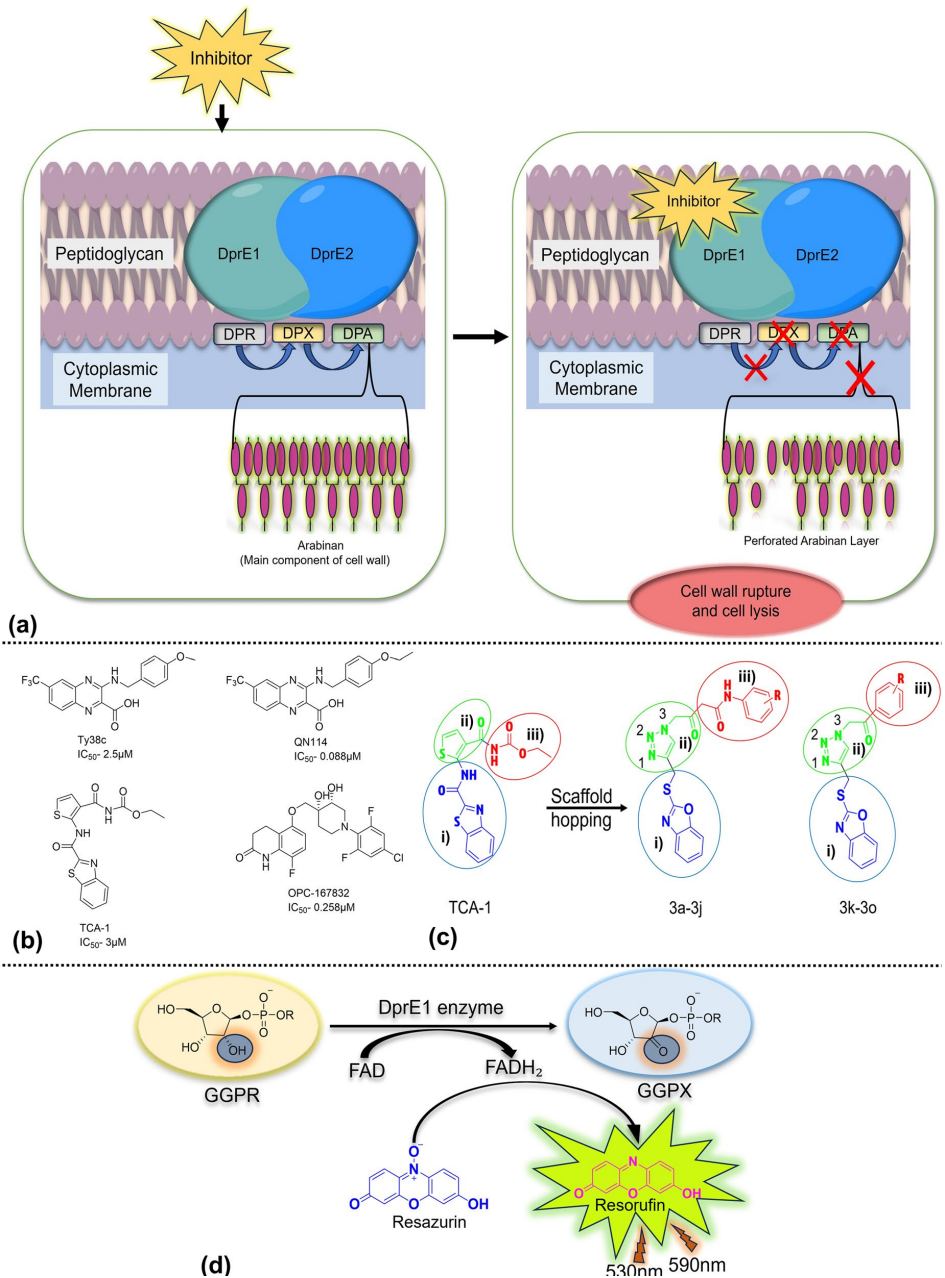
(a) DprE1 in the biosynthesis of arabinan in the cell wall of *Mtb* and the presence of inhibitor leading to interruption in arabinan biosynthesis. (b) Novel drug candidates: non-covalent inhibitors of DprE1 demonstrating IC_50_ values at early stages. (c) Scaffold-hopping strategy: polar tail (in circle iii), hydrophobic head (in circle ii)), and lipophilic trunk (in circle i). Exploration of novel ligands through the chemical structures of DprE1 inhibitor (TCA-1). (d) The DprE1 assay measures DprE1 activity using a fluorescence-based assay that involves the reduction of resazurin to resorufin, while DprE1 catalyses the oxidation of GGPR to GGPX using FAD as the cofactor.

Decaprenylphosphoryl-β-D-ribose-oxidase (DprE1) is one such essential enzyme, catalysing the flavin adenine dinucleotide (FAD)-dependent oxidation of decaprenylphosphoryl-β-D-ribose (DPR) to produce decaprenyl phosphoryl-2′-keto-D-erythro-pentofuranose (DPX). The keto group of DPX subsequently undergoes a reduction by decaprenylphosphoryl-D-2-ketoerythropentose reductase (DprE2), resulting in the formation of decaprenylphosphoryl-β-D-arabino­furanose. This molecule serves as a vital lipid-linked substrate used by membrane bound arabinosyltransferases to construct the essential arabinan layers of the cell wall (Figure 1a)^8^^,^^9^. The specificity of DprE1 for mycobacteria and actinomycetes makes it an enticing target for tuberculosis treatment development^10^^,^^11^.

Numerous compounds that function as inhibitors of DprE1, through non-covalent interactions, have been discovered (Figure 1b). One noteworthy example is TCA-1, a benzothiazole derivative that distinguishes itself for its ability to inhibit both proliferating and non-proliferating forms of Mtb^12^^,^^13^. The distinctive moiety in TCA-1, specifically the thiophene amide, has led us to shift our focus towards the discovery of a new series of 1,2,3-triazole clubbed benzoxazole derivatives, aiming for substantial improvement in TB drug discovery^14^. The goal is to improve their effectiveness and drug-like properties through the implementation of a scaffold-hopping strategy (Figure 1c), expanding on the foundation provided by the lead compound TCA-1^15^.

In the past 20 years, 1,2,3-triazoles have gained prominence as a crucial component in the field of drug development and discovery. This is predominantly attributed to their noteworthy biological efficacy, facile synthesis, stability across diverse conditions, and adaptability to a broad spectrum of chemical reactions^16^. The revolutionary concept, recognised with a Nobel Prize and originating from the Meldal and Sharpless groups, centred on refining the Huisgen 1,3-dipolar cycloaddition involving azides and terminal alkynes. This modification resulted in the production of 1,4-disubstituted 1,2,3-triazoles^14^^,^^16^. 1,2,3-Triazole and benzoxazole-based molecules have diverse biological activities, such as antimicrobial, anti-cancer, and anti-tuberculosis effects^17^^,^^18^. Acetoacetanilide, another intriguing pharmacophore, offers antimicrobial, antioxidant, and antiproliferative properties^19^. These scaffolds have potential for drug discovery, as proven with drugs such as rufinamide and benoxaprofen.

In the present work, we utilised molecular hybridisation that combines bioactive scaffolds to design and synthesise novel 1,2,3-triazole-linked benzoxazole derivatives to explore their anti-tubercular activity, addressing a gap in the previously reported *in-vitro* activity on DprE1 inhibition, which is guided by structure–activity relationships^20^. A biochemical assay elucidated their mechanism of action and affinity towards DprE1 (Figure 1d), emphasising molecular design encompassing synthesis, redox assays, ADMET studies, molecular interaction, and dynamics simulation analyses.

## Results and discussion

### Deciphering unique interaction of TCA-1 with DprE1

**Figure 2.**
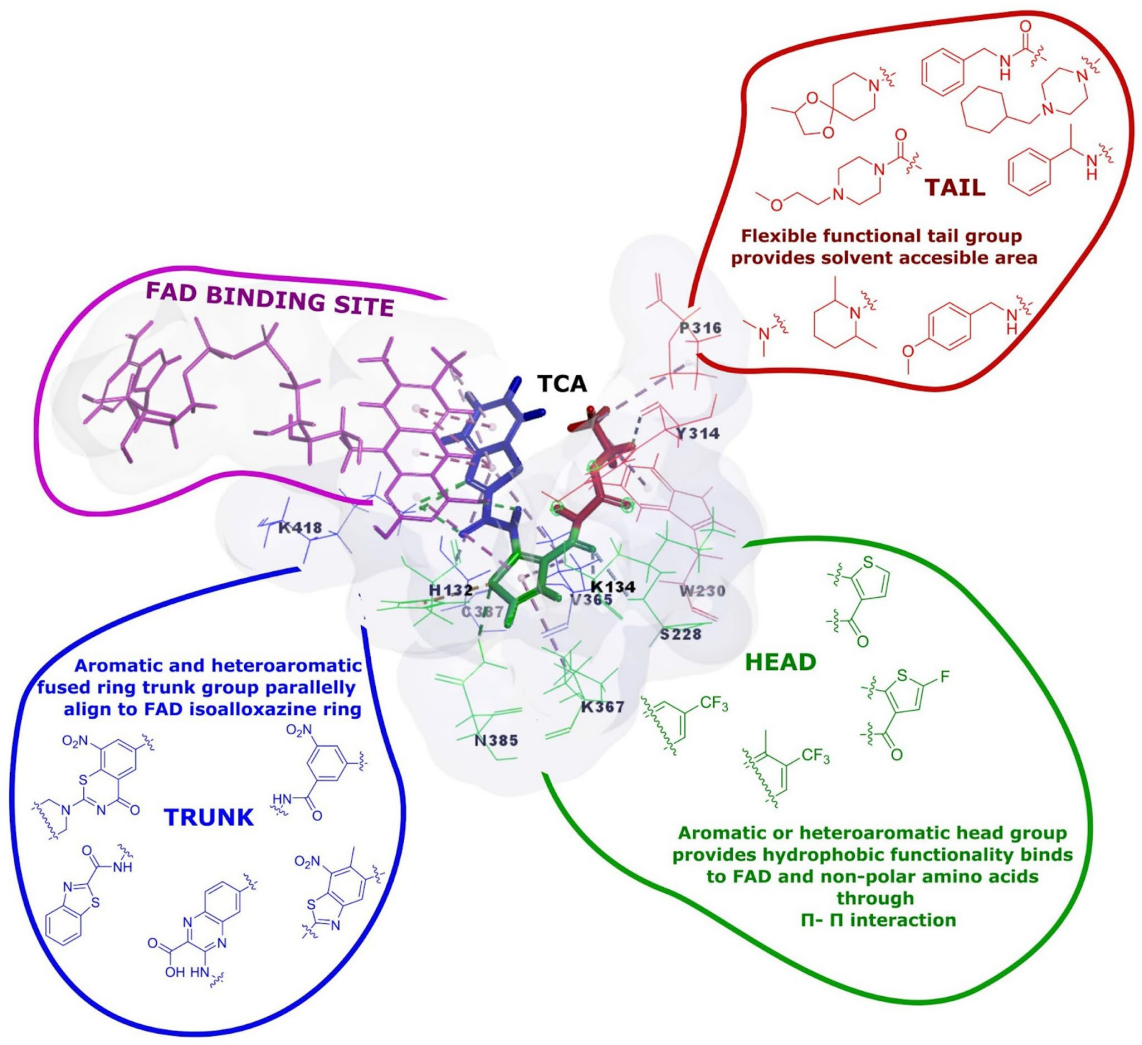
Visualisation of DprE1 enzyme’s functional sites using the TCA-1 inhibitor: mapping polar tail area, hydrophobic head regions, and lipophilic trunk regions. The lipophilic region aligns with FAD’s tricyclic hetero ring. (Inspired by Yadav et al.^10^).

TCA-1, the reported DprE1 inhibitor, has a unique structure of thiophenamide molecule attached to benzothiazole moiety. Driven by the structural interplay and inhibition of DprE1 by the TCA-1 molecule, we sought to study the structure of TCA-1 and its interaction with the protein. The active-site pocket of DprE1 (Figure 2) includes distinct regions: a polar region containing Lys418, His132, and Ser228; a hydrophobic site with Cys387 and Gln334; and a lipophilic area featuring Asn324, Arg325, and Leu317. We identified these regions using Discovery Studio 19.0 software with the PDB ID 4KW5 where the co-crystal ligand is TCA-1. The confirmation of these findings was obtained through the DprE1 protein binding sheet, accessible in the Supporting Information section (see Fig.S46) on the PDBsum server. With this insight, we developed the rationale for designing structures with molecular requirements i.e. having a hydrophobic head (1,2,3-triazole ring), lipophilic trunk (benzoxazole ring), and polar tail (phenacyl/acetoacetanilide) that would align and complement with DprE1 protein, aiming to elicit desired biological responses.

### Chemistry and synthesis

**Scheme 1.**
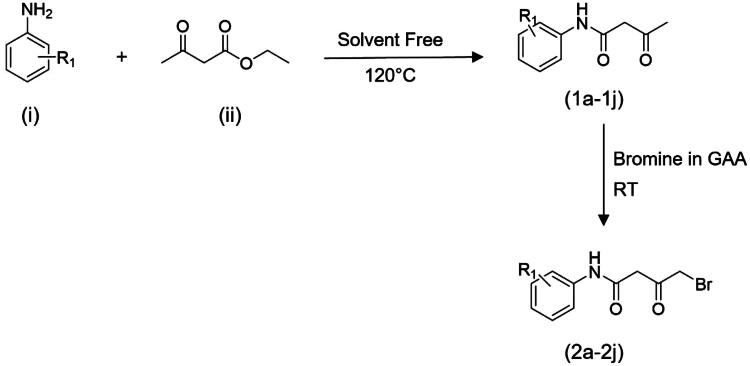
Synthesis pathway for the ω-bromoacetoacetanilides.

**Scheme 2.**
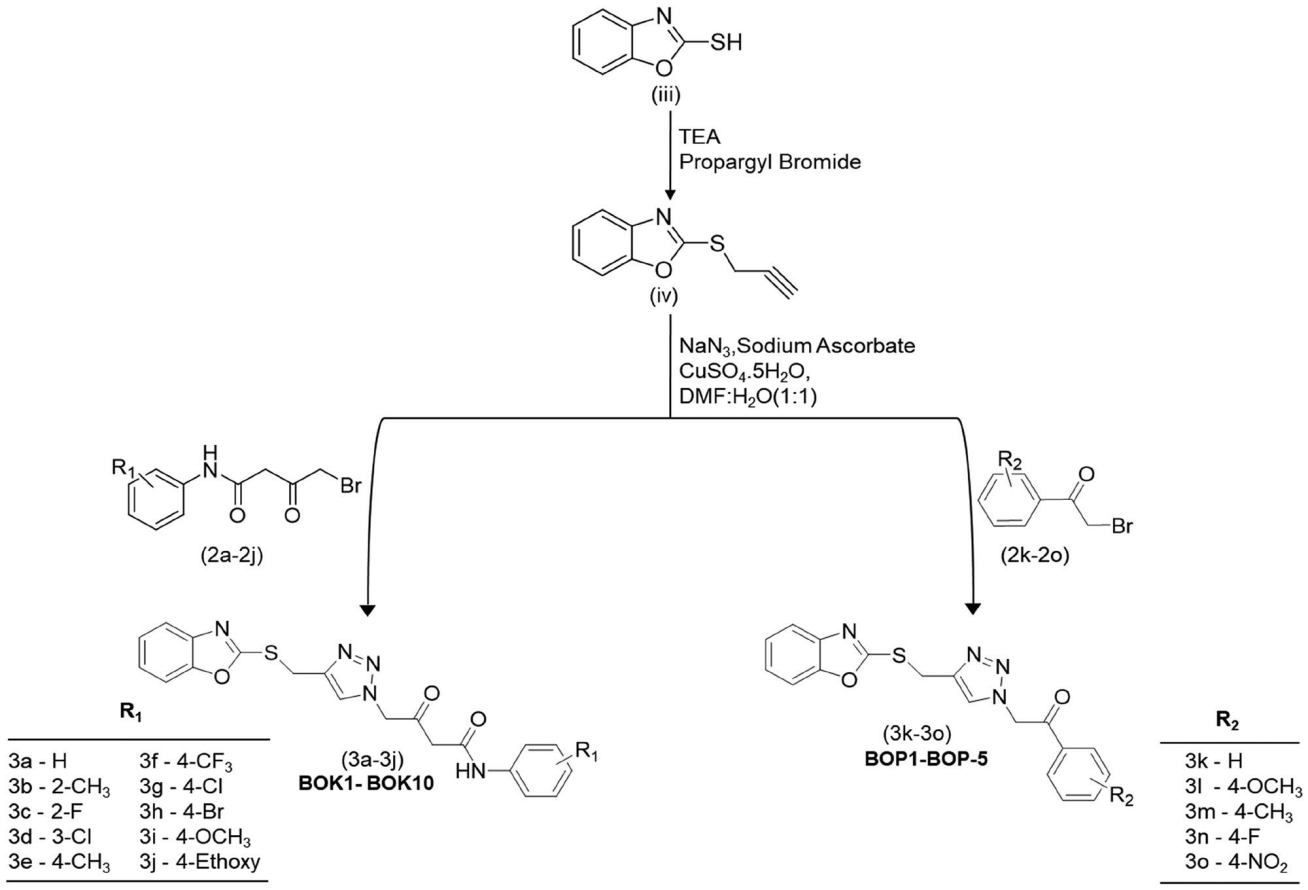
Novel synthesis of 1,2,3-triazole-linked benzoxazole phenacyl/acetoacetanilide derivatives.

The synthesis of the titled compounds involved refluxing commercially available substituted anilines (i) with ethyl acetoacetate (ii) in a solvent-free condition for 2 h at 120 °C, yielding substituted acetoacetanilides (1a − 1j) as outlined in Scheme 1. Subsequently, the intermediates (1a − 1j) underwent bromination by employing a solution of bromine in glacial acetic acid, supplemented with a small crystal of iodine^21^. The reaction solution was stirred for 6–8 h at ambient temperature, leading to the formation of ω-bromo acetoacetanilides (2a − 2j). Phenacyl bromide (2k–2o) was directly procured for use in Scheme 2. The synthesis of thiopropargylated benzoxazole (iv) was initiated with the utilisation of 2-mercapto­benzoxazole (iii) as the substrate and propargyl bromide (80% in toluene). Propargylation occurred at the thiol group located at position 2 of benzoxazole through reflux conditions in absolute ethanol with triethylamine as a basic catalyst, yielding the desired product in a high yield of 95% after 1 h. Subsequently, the final compounds (3a − 3j or **BOK-1–BOK-10**) and (3k − 3o or **BOP-1–BOP-5**) were generated through an azide–alkyne Huisgen cycloaddition reaction. In this process, thiopropargylated benzoxazole was combined with sodium azide, sodium ascorbate, and copper sulphate pentahydrate, along with either substituted ω-bromoacetoacetanilides or substituted phenacyl bromides. This reaction took place in a mixture of dimethylformamide (DMF) and H_2_O (5:5) at ambient temperature. After stirring for 8–12 h, the final compounds were obtained in high yields, ranging from 80% to 95%. This method served as a versatile, one-pot, multicomponent reaction for the synthesis of these compounds, as illustrated in Scheme 2.

In the infra-red (IR) spectra, the presence of signals at ∼1707.06 cm^−1^ and ∼1666.55 cm^−1^ confirms the C = O and C = O stretching vibrations, respectively, indicating the formation of ω-bromoacetoacetanilide (1a − 1j). This is further supported by a distinctive NH stretching signal at ∼3296.45 cm^−1^. Moreover, the integration of the propargyl residue at the thiol group at position 2 of the benzoxazole ring (iv) is confirmed by the presence of two clear bands appearing at approximately 3391 cm^−1^ and 2141 cm^−1^, indicating the existence of the ethyne hydrogen (≡C–H) and the ethyne (C≡C) group, respectively.

The synthesis of compounds 3a–3j and 3k–3o was achieved through the absence of peaks corresponding to C≡C at approximately 2141 cm^−1^ and ≡C–H at around 3391 cm^−1^ in the IR spectra confirming their participation in the cycloaddition reaction. Additionally, proton NMR analysis of the final compounds revealed the absence of signal assigned for the ethyne proton at δH ∼3.25 ppm in the starting S-alkyne, along with the emergence of a singlet at δH ∼8.85 ppm, indicative of –CH proton of the 1,2,3-triazole, further confirming the successful formation of these compounds.

### *Assessment of minimal inhibitory concentration against the H37Rv strain of* Mycobacterium tuberculosis

The compounds under investigation were assessed for their effectiveness in inhibiting the *in-vitro* growth of the *M. tuberculosis* H37Rv strain using the resazurin microtiter assay (REMA). Resazurin, employed as a redox indicator, exhibits a colorimetric transformation in correlation with the growth of *M. tuberculosis*. This alteration in colour was quantitatively assessed to gauge the proliferation of the bacterium. The minimum inhibitory concentration (MIC) denotes the smallest concentration of a substance required to completely halt bacterial growth. All screened compounds demonstrated *in-vitro* inhibitory efficacy against Mtb, with MIC spanning from 1.56 to ≥25.0 μg/mL (Table 1). The series consists of 15 compounds, of which the BOK series has an additional amide functional group along with the carbonyl group with respect to the BOP series. The amide functional group was added to the tail portion of the molecule to study the effect on the potency of the molecule (Figure 1c). From the MIC evaluation, we could conclude that the addition of an amide functional group led to improved potency. The estimated MIC of BOP-1 was 5.9 μg/mL, whereas the introduction of amide functionality increased the potency by approximately twofold (BOK-1 had an MIC of 3.2 μg/mL). Similarly, the potency of BOK-5 was found to have increased by twofold compared to BOP-3 (MIC 11.6 vs 20.5 μg/mL, respectively). The compounds BOK-2 and BOK-3 showed significant inhibitory potency against Mtb with MIC 1.8 and 2.4 μg/mL, respectively. Both the compounds had an amide group and substitution at the ortho position of the phenyl ring (polar portion). In contrast, other compounds having substitutions on the meta and a few para positions of the phenyl ring such as BOK-4, BOK-7, and BOP-4 inhibited Mtb at concentrations i.e. 6.9, 6.7, and 4.8 μg/mL, respectively. Other compounds featuring substitutions of ethoxy, trifluoromethyl, bromo, and nitro groups, positioned para to the phenyl ring showed activity >25 μg/mL (Table 1).

**Table 1.** Assessment of 1,2,3-triazole clubbed benzoxazole derivatives for *in-vitro* antitubercular evaluation (MIC_50_), cytotoxicity (IC_50_), selectivity index, and DprE1 inhibition assay.

S. no.	Compound	*Mtb H37Rv MIC*_50_ (μg/mL)	**3T3 cell** **IC_50_(μg/mL)**	Selectivity index*	**DprE1 inhibition** **assay IC_50_ (μM)**
1.	**BOK-1**	**3.2**	**118.5**	**37.07**	**5.2 ± 0.6**
2.	**BOK-2**	**1.8**	**103.92**	**57.77**	**2.2 ± 0.1**
3.	**BOK-3**	**2.4**	**112.1**	**46.75**	**3.0 ± 0.6**
4.	BOK-4	6.9	ND	ND	ND
5.	BOK-5	11.6	ND	ND	ND
6.	BOK-6	39.6	ND	ND	ND
7.	BOK-7	6.7	ND	ND	ND
8.	BOK-8	44.1	ND	ND	ND
9.	BOK-9	10.6	ND	ND	ND
10.	BOK-10	42.6	ND	ND	ND
11.	**BOP-1**	**5.9**	**51.69**	**8.76**	**3.3 ± 1.0**
12.	BOP-2	19.06	ND	ND	ND
13.	BOP-3	20.5	ND	ND	ND
14.	**BOP-4**	**4.8**	**55.8**	**11.62**	**3.1 ± 0.7**
15.	BOP-5	32.3	ND	ND	ND
16.	**TCA-1**	ND	ND	ND	**3.0 ± 0.2**
17.	**Rifampicin**	**< 0.004**	ND	ND	ND
18.	**5-Fluorouracil**	ND	**53.72**	ND	ND

*Selectivity index = IC_50_ (μg/mL)/MIC (μg/mL).

ND = Not done. The bold numbers represents compounds and standard drugs with significant activity.

### Assessment of derivatives on mouse fibroblast 3T3-cell replication

**Figure 3.**
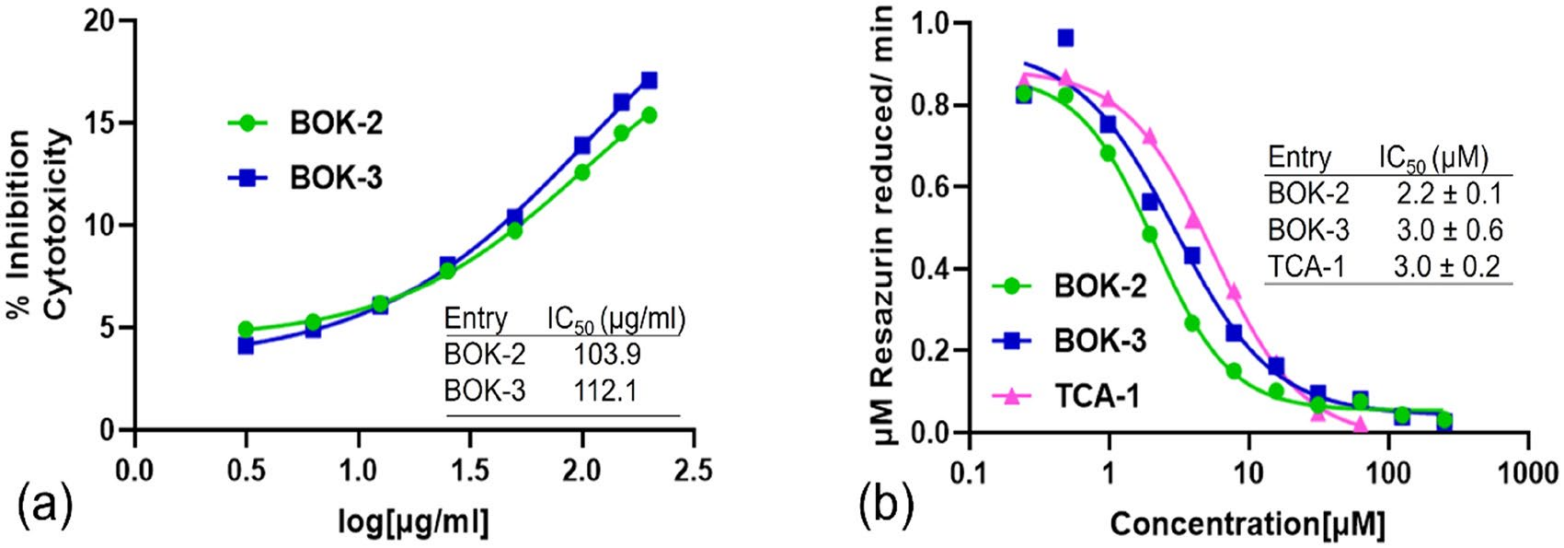
Assessment of synthesised compounds through *in-vitro* testing. (a) Effect of derivatives (3.13, 6.25, 12.5, 25, 50 100 150 and 200 μg/mL) on mouse fibroblast 3T3-cell proliferation showing IC_50_ of 103.9 μg/mL (BOK-2) and 112.1 μg/mL (BOK-3). (b) DprE1 inhibitory concentration − response graph of derivatives BOK-2 and BOK-3 (0.24 to 250.00 μM) determined by the DprE1 inhibition assay.

In the early stages of drug discovery, potential candidates are typically tested against mammalian cell lines, such as mouse fibroblast 3T3 cells, to gauge any potential cytotoxic effects. The safety assessment of compounds BOK-1, BOK-2, BOK-3, BOP-1, and BOP-4, each exhibiting MIC values of ≤6.25 μg/mL, was conducted using the MTT assay against mouse fibroblast 3T3 cells. The increase in cellular mortality implies a reduction in the enzymatic conversion of MTT dye to formazan within the mitochondria. Exposure to escalating concentrations (ranging from 3.13 to 200 μg/mL) of test compounds during a 48-h incubation led to a concentration-dependent decline in cell vitality relative to the control group (Figure 3a). Our results reveal that concentrations of up to 50 μg/mL were permissible for all the compounds tested. Based on these observations, we extended the concentration range to 100 μg/mL, as there was minimal inhibition observed, with values not even crossing 20%. In contrast, the cytotoxicity concentration for the standard, 5-fluorouracil, at 50 μg/mL exceeds 42% inhibition. The corresponding percentage inhibition data are presented in Table 1. The selectivity index (SI) is determined by the ratio between *in-vitro* cytotoxicity (μg/mL) and antimycobacterial activity (μg/mL). The compounds BOK-2 and BOK-3, which demonstrated the most promising antitubercular activity, exhibited an SI > 45, indicating a substantial safety margin (Table 1).

### DprE1 protein inhibition through the in-vitro DprE1 inhibition assay

For the identification and validation of the biological target for this new series of 1,2,3-triazole-linked benzoxazoles, compounds displaying antimycobacterial activity below 6 μg/mL and showcasing diverse side chains were assessed against the purified DprE1 protein (Table 1). The assessment of the compounds’ ability to inhibit DprE1 activity was conducted through an *in-vitro* assay. This involved purified DprE1 protein, geranylgeranyl-phosphoryl-β-d-ribose (GGPR) substrate, and resazurin as a redox indicator. The synthesised molecules were tested for their impact on DprE1 activity within this experimental setup. The initial rates of activity, characterised by increasing concentrations of the compound, were measured and compared to the control compound TCA-1. The half maximal inhibitory concentration (IC_50_) was then calculated for each synthesised compound, quantitatively assessing their inhibitory effect. Compounds BOK-2 and BOK-3 (Figure 3b) significantly inhibited DprE1 protein with IC_50_ values of 2.2 ± 0.1 μM and 3.0 ± 0.6 μM, respectively (see Fig. S47 of Supporting Information for the concentration-response graph of other compounds), demonstrating a strong correlation with the MIC values observed for the tested compounds. The promising results prompted us to delve deeper into these compounds through initial assessments of their druggability profiles and validation via computational studies. This approach aims to enhance our understanding of the structural features involved in the inhibition mechanism of the DprE1 enzyme, utilising docking and molecular dynamics (MD) simulation studies.

### Exploration of binding affinity and intermolecular interactions via molecular docking analysis

**Figure 4.**
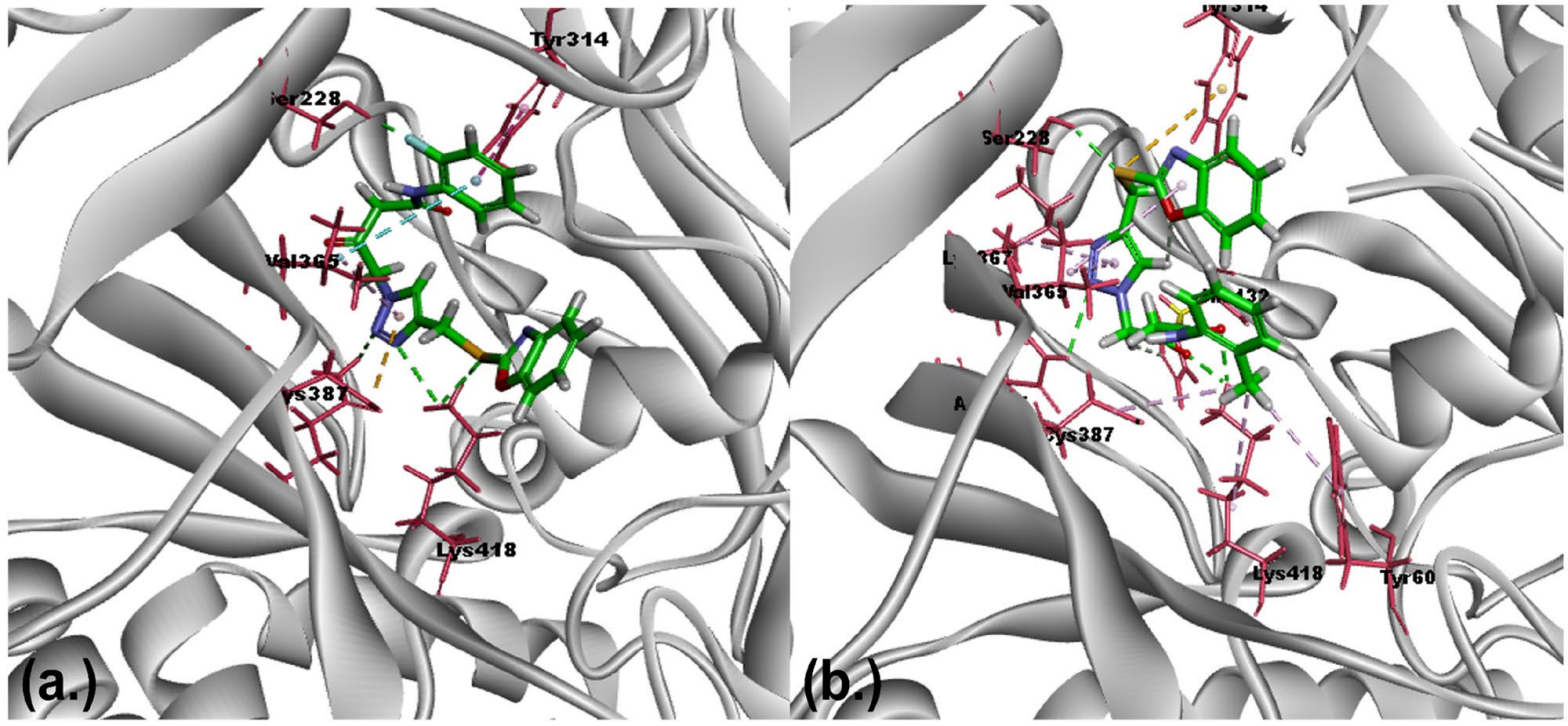
Assumed binding mode of DprE1 with ligands: molecular interactions with BOK-2 and BOK-3 (a) Molecular alignment of compound BOK-2 within the DprE1 binding pocket: exposing hydrogen bonding and hydrophobic interactions with amino acids His132, Ser228, Tyr314, Lys367, Val365, Asn385, Cys387, Tyr60, and Lys418. (b) Molecular alignment of compound BOK-3 within the DprE1 binding pocket: exposing hydrogen bonding and hydrophobic interactions with amino acids Tyr314, Ser228, Val365, Cys387, and Lys418.

Active compounds demonstrating IC_50_ values within the range of TCA-1 were selected for docking studies within the DprE1 binding pocket. The primary objective of molecular docking analysis is to unravel the binding pose of molecules, providing insights into their inhibitory potency through an understanding of intermolecular interactions with the target protein. In docking studies, the CDOCKER docking application, which utilises the CharmM force field, evaluates results based on -CDOCKER energy and -CDOCKER interaction energy^22^. -CDOCKER energy accounts for ligand internal strain and receptor–ligand interactions, while -CDOCKER interaction energy describes non-bonded interactions like van der Waals and electrostatic forces. Negative values for both energies indicate favourable binding between the protein and ligand^23^^,^^24^. Remarkably, the synthesised compounds BOK-2 and BOK-3, which showed the maximum potency (IC_50_), were also observed to bind to the active site, consistent with information documented in the RCSB crystallography database. These compounds exhibited maximum docking energy and interaction energy values of (–)59.05 and (–)58.65 kcal/mol, respectively (Table 2) (see Table S1 in the supplemental material for other compounds’ molecular docking scores). To validate the docking protocol, we re-docked the most potent compound, revealing a minimal root mean square deviation (RMSD) value of 0.532 Å for the bioactive conformations^25^. Through these studies, key amino acid residues, including Lys418, His132, Ser228, Cys387, and Gln334, have been identified as crucial for binding. Additional insights into how these residues contribute to forming hydrogen bonds and participating in hydrophobic interactions, can be found in the Supporting Information (see Fig. S46). In Figure 4, the alignment of essential amino acids with the designed ligands within the complex of DprE1 inhibition is depicted (see Fig. S48 of Supporting Information for further details). To enhance our comprehension of the dynamics and stability MD simulations were conducted.

**Table 2.** Molecular docking scores and DprE1 inhibition IC_50_ values for synthesised compounds with different substitutions.

S.no.	Compound code	Substitution	Position of substitution	(-) CDocker energy	(-) CDocker interaction energy	DprE1 inhibition IC_50_ (μM)
1	BOK-1	H	–	36.04	52.16	5.2 ± 0.6
2	**BOK-2**	**Methyl**	**Ortho**	**40.10**	**59.05**	**2.2 ± 0.1**
3	**BOK-3**	**Fluoro**	**Ortho**	**39.12**	**58.65**	**3.0 ± 0.6**
4	BOP-1	H	–	28.18	48.12	3.3 ± 1.0
5	BOP-4	Fluoro	Para	28.11	47.36	3.1 ± 0.7
6	TCA-1	–	–	35.35	55.98	3.0 ± 0.2

### Dynamic stability of DprE1 protein in complex with BOK-2 and BOK-3 along with cofactor FAD through MD simulation analysis

**Figure 5.**
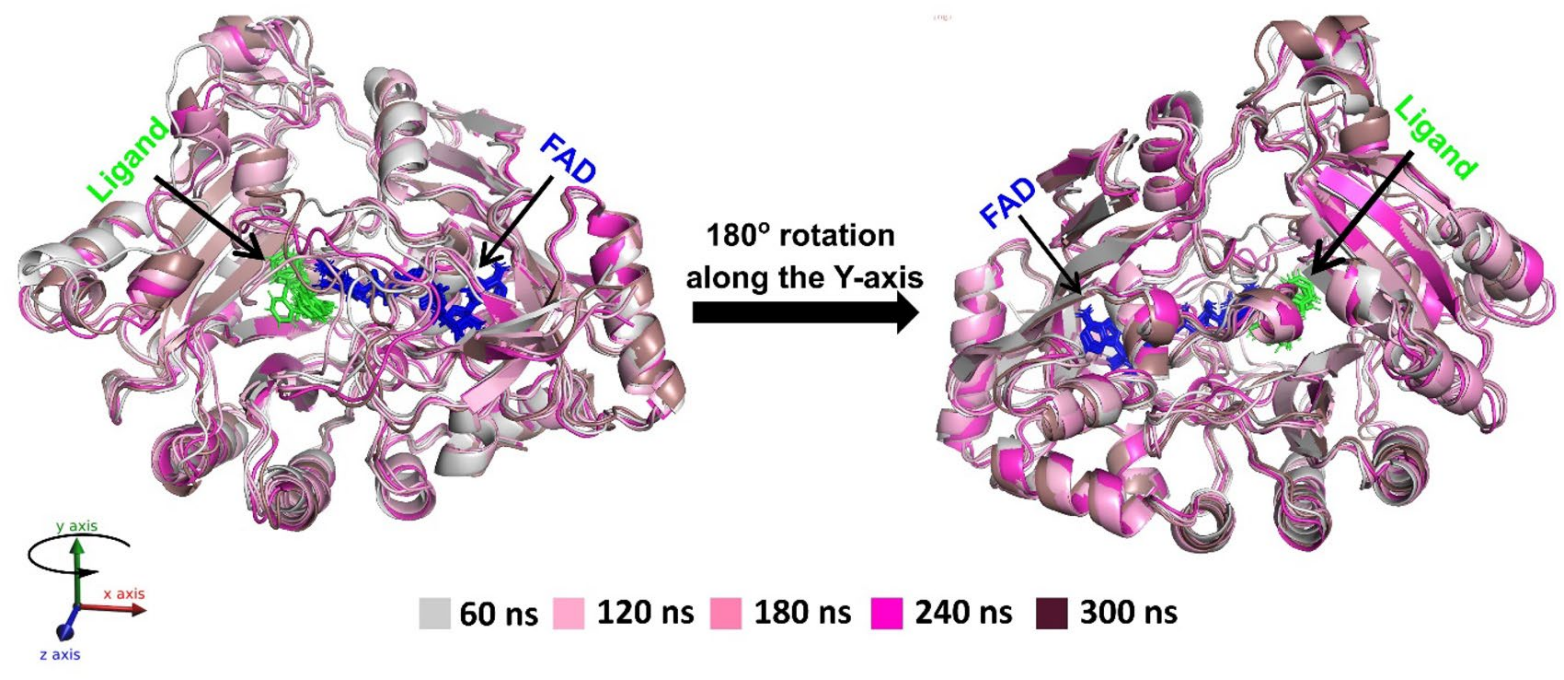
Superimposition of ligand (BOK-3) bound DprE1 protein along with FAD complex throughout trajectory in time-lapse at 60th, 120th, 180th, 260th, and 300th ns time step.

While docking offers a static view, MD offers a dynamic understanding by conducting long-range time-dependent studies confirming the binding pose observed in docking. The binding pose of the ligand is consistent with the reference molecule and is maintained throughout the MD simulation run. The study began with 300 ns production runs to observe the behaviour of ligand-free DprE1 protein and its complexes with BOK-2, BOK-3, and TCA-1, independently. Dynamics of active ligands within the DprE1 ligand binding domain (LBD), alongside cofactor FAD, were studied over time in a solvated environment^26^. Superimposed structures at regular intervals (60th, 120th, 180th, 240th, and 300th ns) reveal conformational fluctuations in the presence of ligand (BOK-3) at the LBD, complemented by FAD binding (Figure 5). During simulations, the cofactor FAD remained intact, consistent with literature suggesting its complementary role in ligand binding to the LBD through intermolecular hydrogen bonds.

**Figure 6.**
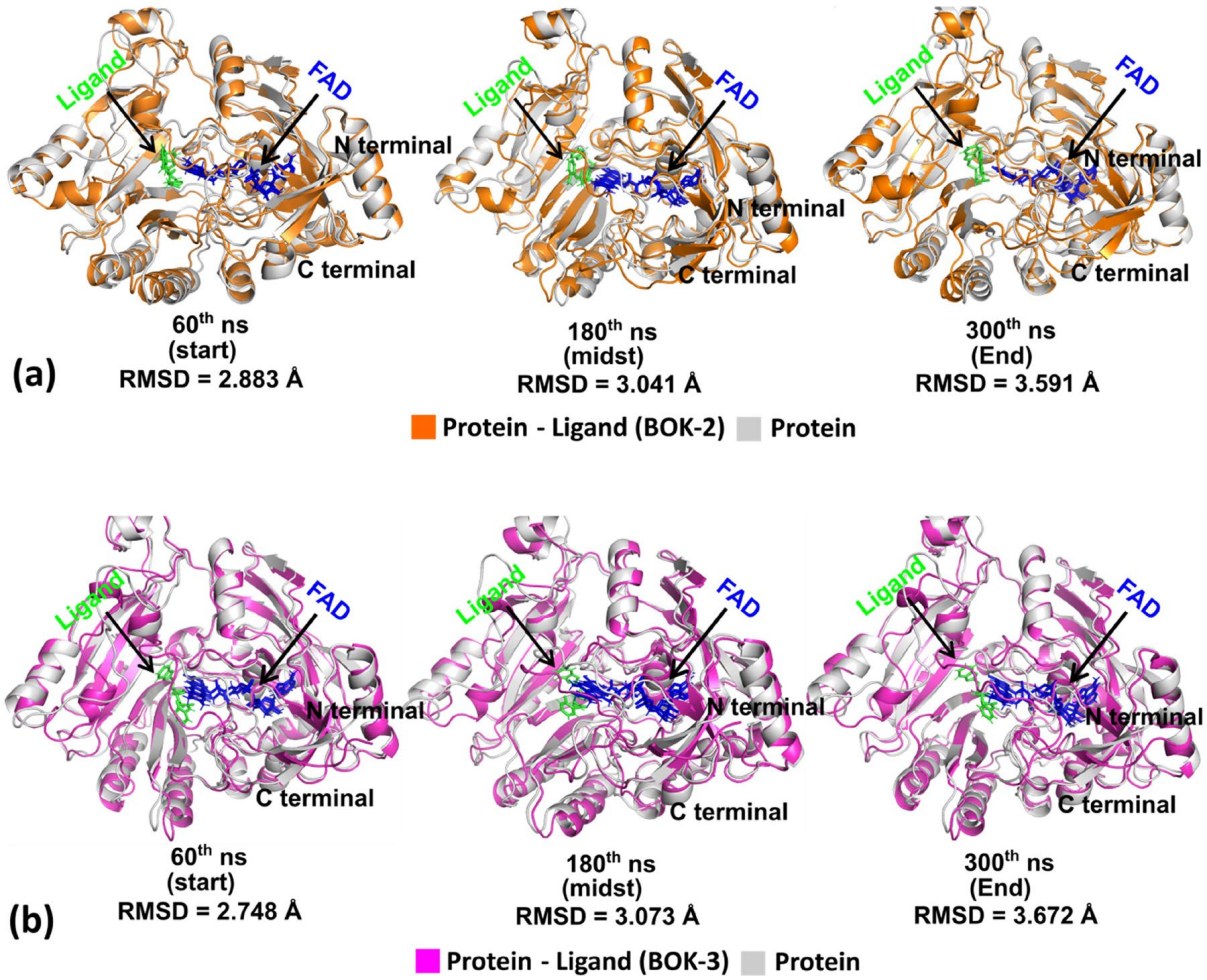
(a) and (b) Progressive superimposition of trajectory conformations of DprE1 complex with ligand-free protein and with ligands (**BOK-2 and BOK-3**) at time points 60th, 180th, and 300th ns.

Trajectory analysis (Figure 6a and b) illustrates conformational changes, with structures superimposed in the absence and presence of ligand at intervals (60th, 180th, and 300th ns) (see Fig.S49 in the supplemental material for other time intervals). Initially, protein structures without and with ligands overlapped precisely, but larger deviations in RMSD values were observed as the MD run progressed, while the ligands, FAD binding pose, and secondary structures remained intact.

**Figure 7.**
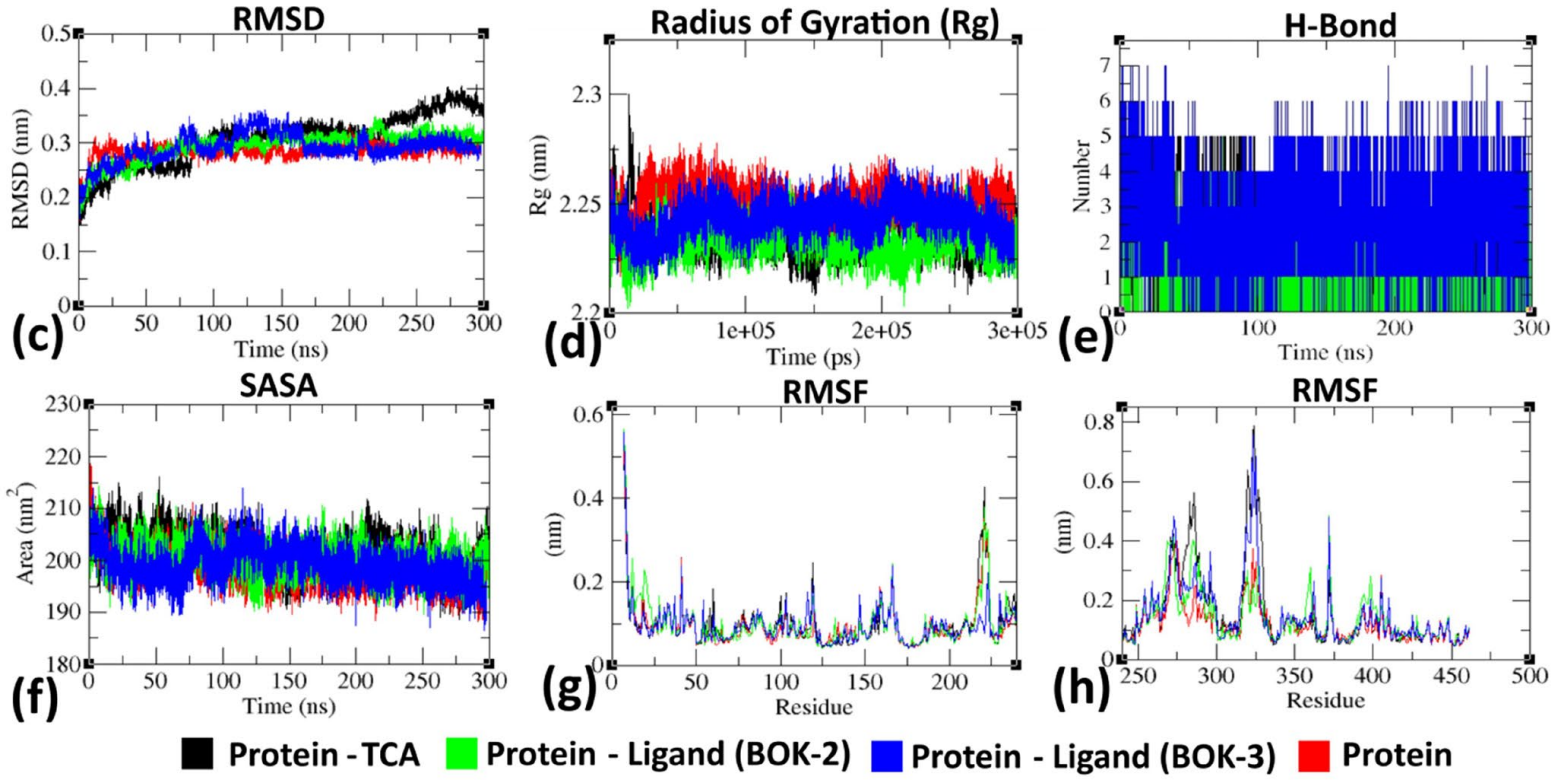
Evaluation of RMSD, Rg, hydrogen bond formation, SASA, and RMSF of DprE1 (protein) with BOK-2, BOK-3, and TCA-1 (ligand) complexes at 300,000 ps (300 ns). (c) Temporal changes in backbone RMSD of DprE1 protein with and without ligand complexes. (d) Variation of protein backbone Rg between its unbound and complexed states throughout the simulation duration. The *y*-axis represents Rg (nm), while the *x*-axis depicts the time interval (ps). (e) Temporal evolution of hydrogen bonds between protein and ligand throughout simulation (ns) (f) Time-dependent SASA analysis. The *y*-axis represents SASA (nm), while the *x*-axis denotes time (ns). (g) and (h) Comparison of residue-wise average RMSF plot between protein in native and ligand-bound states.

Analysis of RMSD (Figure 7c) reveals that the equilibrium of the C–α atoms for both unliganded and ligand-bound protein assemblies was reached around 75–90 ns, followed by stable trajectories with minimal deviation (0.10 − 0.15 nm), suggesting enhanced structural stability in ligand-bound protein assemblies. As per Figure 7d, the ligand-free protein showed reduced gyration fluctuations (∼0.15 nm) compared to the stable trajectories observed in the ligand-bound protein (∼0.05 nm). Furthermore, the formation of intermolecular hydrogen bonds between ligands (BOK-2, BOK-3, and TCA-1) and LBD at different time frames indicated protein-ligand stability and biological responses^25^^,^^27^. The hydrogen bond count remained constant over time, consistent with molecular docking results (Figure 7e). The solvent-accessible surface area (SASA) values (Figure 7f) consistently fluctuated within the range of 10–20 nm^2^ approximately^28^. Root Mean Square Fluctuation (RMSF) analysis (Figure 7g and h) revealed residue-wise fluctuations, with crucial amino acids exhibiting increased stiffness in the complex compared to the protein’s native state. These findings are consistent with residues identified during the molecular docking experiment^29–31^. Overall, the simulation outcomes provide valuable insights into how ligands (BOK-2 and BOK-3) interact with the DprE1 protein, revealing their bioactive characteristics.

### Computational drug-Likeness and ADMET prediction for safety evaluation

Ensuring optimal oral absorption and a favourable ADMET profile is crucial for drug success, reducing late-stage trial setbacks. Utilising computational tools like http://www.swissadme.ch/, we forecasted ADME attributes (Supporting Information, see Table S2) within the recommended ranges for 95% of known drugs. SMILES notation facilitated rapid predictions, encompassing gastrointestinal absorption, bioavailability, CYP enzyme inhibition, blood–brain barrier permeability, skin permeability, and pan-assay interference compounds (PAINS) alerts. Topological polar surface area (TPSA) indicates polarity impacting absorption and blood–brain barrier traversal, while iLOGP reflects solvation-free energy. ESOL LogS indicates hydrophilicity, and cytochrome-P450 isozymes influence metabolism and drug removal, with enzyme suppression leading to interactions, accumulation, and adverse effects. PAINS alerts aid in detecting false positives in drug discovery^31^^,^^32^.

Among the synthesised compounds, no molecules exhibit violations in Lipinski’s rule, indicating that these compounds were most probably orally active. The synthesised compounds were predicted to have logP values ranging from 2.07 to 3.29. As we are concerned with anti-tubercular potential, the logP value is a crucial parameter contributing to drug activity. The active drugs BOK-2 and BOK-3 reveal predicted logP values of 2.7 and 2.54, respectively, which is found to be better than the reference standard TCA-1 (1.81). Notably, the predicted blood–brain barrier penetration characteristics and PAINS alert were favourable for the synthesised compounds (supplementary data). Overall, these preliminary screening data suggested that the synthesised compounds can be promising drug candidates that can be taken forward for further evaluation in the drug discovery pipeline.

### SAR analysis of 1,2,3-triazole clubbed benzoxazole derivatives

Piton et al.^32^ and Chikhale et al.^6^, extensively characterised DprE1 protein, revealing three distinct regions – head, trunk, and tail – based on ligand conformations (TCA-1). The binding pocket consistently houses FAD, supporting ligand binding. The head region forms a hydrophobic cavity, the trunk is flanked by FAD and specific residues, and the tail is flexible, featuring crucial amino acids. These insights deepen our understanding of DprE1’s structure and guide potential drug-targeting strategies.

Inspired by DprE1’s characterisation, we designed novel compounds with 1,2,3-triazole clubbed benzoxazole moieties (Figure 1c). Our efficient and cost-effective synthetic scheme ensures timely synthesis, combining structural innovation with practical considerations. *In-vitro* screening against Mtb H37Rv was conducted for compounds (3a–3j and 3k–3o), wherein their antimycobacterial activity was determined and reported as MIC_50_ (minimum inhibitory concentration inhibiting 50% bacterial growth). For effective structure–activity relationship (SAR), variations in chain length and substitutions involving the phenyl moiety were introduced at the N-3 position of the triazole nucleus.

Introducing the amide group via ω-bromo acetoacetanilide (BOK series) enhanced antimycobacterial and DprE1 inhibition activity twofold compared to substituted phenacyl bromide (BOP series) (BOP-1: 5.9 μg/mL, BOK-1: MIC 3.2 μg/mL). Substitution patterns (ortho, meta, para) of the phenyl ring in the side chain influenced both activities. Ortho-substitution, as seen in BOK-2 (methyl at ortho), displayed superior activity (MIC 1.8 μg/mL, IC_50_ 2.0 μM in DprE1 inhibition), while para-substitution, as in BOK-5 (methyl at para), showed lower activity (11.6 μg/mL) and no enzyme inhibition. BOK-3 (fluoro atom at ortho) demonstrated MIC 2.4 μg/mL and IC_50_ 3.0 μM in DprE1 inhibition activity.

Furthermore, a comparison between BOK-4 (chloro at meta position) and BOK-7 (chloro at para position) highlights the favourable activity of meta-substitution over para-substitution. BOK-4 exhibited higher activity at 3.7 μg/mL, while BOK-7 showed lower activity at 6.9 μg/mL. Docking studies were conducted to elucidate the inhibition of the DprE1 enzyme by the synthesised compounds. The *in-silico* analysis reinforced the evidence that ortho-substitution is the most active. BOK-2 and BOK-3 displayed the highest -CDocker energy (40.1096 and 39.1274, respectively) and -CDocker interaction energy (59.0549 and 58.6535, respectively). These two ligands underwent simulation studies to assess stability under various physiological conditions.

## Conclusions

In the present paper, we explore the potential of synthetic molecules to modulate DprE1, offering a promising new pathway for developing effective therapeutic interventions against tuberculosis. Until now, a limited number of scholarly publications have focused on leveraging the 1,2,3-triazole moiety to inhibit the DprE1 protein. We showcased an innovative synthetic scheme for the purposeful design of 15 1,2,3-triazole fused benzoxazole compounds (BOK-1–BOK-10 and BOP-1–BOP-5), employing the Cu(I)-catalysed cycloaddition method as a highly effective catalyst under mild conditions. The MIC against the Mtb H_37_R_v_ strain was determined using the REMA assay for the newly synthesised derivatives. The SAR analysis highlighted that introducing the amide moiety in the BOK series resulted in improved antimycobacterial activity. Amongst all the compounds, BOK-2 and BOK-3 showed significant anti-TB activity with MIC i.e. 1.8 and 2.4 μg/mL, respectively. Upon evaluating the DprE1 inhibitory activity, compounds BOK-2 and BOK-3 demonstrated IC_50_ values of 2.2 ± 0.1 and 3.0 ± 0.6 μM, respectively, as compared to the standard drug TCA-1 (IC_50_ = 3.0 ± 0.2 μM). The *in-silico* results were also aligned with the outcomes of the biochemical assessments. The docking studies not only suggested the binding pose of protein–ligand interaction but also highlighted the crucial amino acids involved in pivotal binding interactions, including Lys418, His132, Ser228, Cys387, and Gln334, like the literature-reported molecule (TCA-1). The MD simulation study conducted for 300 ns validated the docking study and provided valuable structural insights highlighting the stability of the protein–ligand complex.

## Experimental section

### Chemistry

All the chemicals and solvents were procured from Sigma-Aldrich and used without further purification. Intermediates and final products were verified for purity using thin-layer chromatography (TLC), IR, 1HNMR, 13CNMR, and mass spectroscopy. TLC analysis employed Kieselgel 60 F254 aluminium sheets with solvent system of n-hexane/ethyl acetate/methanol (3:2:0.5). Melting points were determined using standard apparatus and were reported without correction. IR spectra were obtained using KBr pellets with a Shimadzu FTIR 8400-S spectrophotometer. LC/MS spectra were acquired with electrospray (ES) ionisation at −70 eV. NMR spectra were recorded in dimethylsulphoxide (DMSO)-d6 at 298K using a Bruker 400 MHz FT-NMR spectrophotometer with reference to DMSO-d6 at δH 2.50 ppm for proton NMR and δC 39.5 ppm for carbon NMR. Resonance patterns were characterised as s (singlet), d (doublet), t (triplet), q (quartet), or m (multiplet), with additional coupling constants (J) provided.

#### Substituted acetoacetanilide synthesis (1a − 1j)

A solution containing ethyl acetoacetate (0.01 M) and substituted aniline (0.01 M) was prepared and subjected to reflux for approximately 2–3 h. Following the reflux, the resulting yellowish/brown liquid was further heated on a water bath to eliminate the alcohol by-product generated during the reaction^33^. After allowing the reaction solution to reach ambient temperature, the crude solid product was obtained by filtration and washed with ether. The purification process involved recrystallization, utilising a solution comprising 25% alcohol in water^33^^,^^34^. The yields proved quite promising, with results ranging from 68% to 98% as white to colourless crystals.

#### ω-bromoacetoacetanilides synthesis (2a − 2j)

In a reaction vessel, a mixture comprising 0.022 M of substituted acetoacetanilide dissolved in 12 ml of glacial acetic acid underwent gradual addition of a bromine solution. This solution, consisting of 0.022 M of bromine, dissolved in 17 ml of glacial acetic acid and containing a small crystal of iodine^17^^,^^21^ was added slowly over the course of 1 h at ambient temperature. After the addition, the solution was further stirred for an additional 6–8 h until the reaction reaches completion, confirmed by TLC analysis. Subsequently, the solution was poured into water, resulting in the formation of ω-bromo substituted acetoacetanilides^35^. The product was isolated by crystallisation, using ethanol as the solvent, yielding a white amorphous product with a yield of 85%–90%.

#### Synthesis of thiopropargylated benzoxazole (iv)

A solution consisting of 10 mM of 2-mercapto benzoxazole dissolved in 30 ml of absolute ethanol, along with 12 mM of triethylamine (Et3N) and 12 mM of propargyl bromide, was introduced with continuous stirring. The resulting mixture was then heated to 90 °C for a duration of 1 h. The surplus solvent was then evaporated using a rotary evaporator. Afterwards, the solid product obtained underwent a thorough washing procedure with cold water. Finally, it was subjected to recrystallization from ethanol, resulting in the isolation of the compound in a high yield of 94%, forming colourless crystals.

#### Synthesis of 1,2,3-triazole clubbed benzoxazole acetoacetanilide/phenacyl derivatives (3a − 3j and 3k − 3o)

In a round bottom flask, a solution containing thiopropargylated benzoxazole (1 M equiv), substituted ω-bromo acetoacetanilide/substituted phenacyl bromide (1 M equiv), and sodium azide (1.1 M equiv) in a mixture of DMF and H_2_O in a 1:1 (v/v) ratio was prepared. To this blend, CuSO_4_·5H_2_O (0.15 M equiv) and sodium ascorbate (0.30 M equiv) were introduced^36^. The ensuing solution was agitated at ambient temperature for 8–12 h, while the reaction advancement was tracked using TLC. After the completion of the reaction, the solution was cooled, and the reaction was halted by pouring it into ice water^37^. The resultant solid product was isolated via filtration and rinsed with water. Following this, the product underwent recrystallization from ethanol, yielding 82%–95%.

##### 4–(4-((Benzo[d]oxazol-2-ylthio)methyl)-1H-1,2,3-triazol-1-yl)-3-oxo-N-phenyl butanamide(3a) (see Fig. S1,S2,S3):

Light brown solid, yield 85%, m.p.:161 °C − 165 °C, FTIR νmax (KBr, cm^−1^): 3366.20(N–H str.), 3153.26 (C–H str., triazole ring), 2891.42 (C–H str.,al), 1707.06 (C = O), 1663.20 (C = O str., amide), 1510.30, 1452.34 (C = C str., Ar), 697.50 (C–S str.) cm^−1^.1H NMR (δ ppm, DMSO-d6): 3.726 (s, 2H, CH_2_), 4.719 (s, 2H, CH_2_), 5.600 (s, 2H, CH_2_), 7.070 (t, 1H, ArH, *J* = 7.6 Hz), 7.080 (t, 2H, ArH, *J* = 7.6 Hz), 7.317 (t, 1H, ArH, *J* = 7.6 Hz), 7.335 (t, 1H, ArH, *J* = 7.6 Hz), 7.550 (d, 2H, ArH, *J* = 7.6 Hz), 7.569 (d, 1H, ArH, *J* = 2.8 Hz), 7.668 (d, 1H, ArH, *J* = 6.8 Hz), 8.086 (s, 1H, CH), 10.167 (s, 1H, NH).13C NMR (δ ppm, DMSO-d6): 26.890, 49.282, 58.641, 110.742, 118.847, 119.404, 119.644, 124.087, 124.870, 125.135, 128.910, 129.265, 139.111, 141.676, 151.813, 164.813, 164.801, 197.905. MS (m/z): M + 1 analysed 408.12, M + 1 predicted 408.46.

##### 4–(4-((Benzo[d]oxazol-2-ylthio)methyl)-1H-1,2,3-triazol-1-yl)-3-oxo-N-(o-tolyl) butanamide(3b) (see Fig. S4,S5,S6)

Light brown solid, yield: 89%; m.p.: 172 °C–176 °C; FTIR νmax (KBr, cm^−1^): 3357.60 (N–H str.), 3134.20 (C–H str., triazole ring), 2871.32 (C–H str., al), 1710.16 (C = O), 1643.70 (C = O str.,amide), 1520.20, 1472.14 (C = C str., Ar), 687.43 (C–S str.) cm^−1^. 1H NMR (δ ppm, DMSO-d6): 2.192 (s, 3H, CH_3_), 3.756 (s, 2H, CH_2_), 4.720 (s, 2H, CH_2_), 5.597 (s, 2H, CH_2_), 7.093 (d, 1H, ArH, *J* = 6.4 Hz), 7.183 (d, 1H, ArH, *J* = 7.2 Hz), 7.223 (t, 1H, ArH, *J* = 7.2 Hz), 7.347 (t, 1H, ArH, *J* = 6.0 Hz), 7.406 (t, 1H, ArH, *J* = 7.6 Hz), 7.425 (t, 1H, ArH, *J* = 7.6 Hz), 7.656 (d, 1H, ArH, *J* = 6.4 Hz), 7.672 (d, 1H, ArH, *J* = 6.4 Hz), 8.098 (s, 1H, CH), 9.524 (s, 1H, NH).13C NMR (δ ppm, DMSO-d6): 18.240, 26.909, 48.642, 58.620, 110.749, 118.857, 124.890, 124.995, 125.146, 125.337, 125.898, 126.436, 130.756, 130.812, 132.039, 136.262, 141.665, 151.808, 164.642, 165.800, 198.136. MS (m/z): M + 1 analysed 422.13, M + 1 predicted 422.48.

##### 4–(4-((Benzo[d]oxazol-2-ylthio)methyl)-1H-1,2,3-triazol-1-yl)-N-(2-fluorophenyl)-3-oxobutanamide(3c) (see Fig. S7,S8,S9):

Light brown solid, yield: 91%; m.p.: 175 °C–179 °C; FTIR νmax (KBr, cm^−1^): 3417.10 (N–H str.), 3124.40 (C–H str., triazole ring), 2861.62 (C–H str., al), 1712.10 (C = O), 1653.60 (C = O str., amide), 1530.10, 1462.24 (C = C str., Ar), 689.13 (C–S str.) cm^−1^. 1H NMR (δ ppm, DMSO-d6): 3.810 (s, 2H, CH_2_), 4.726 (s, 2H, CH_2_), 5.582 (s, 2H, CH_2_), 7.010 (t, 1H, ArH, *J* = 7.2 Hz), 7.161 (t, 1H, ArH, *J* = 6.0 Hz), 7.263 (d, 1H, ArH, *J* = 7.2 Hz), 7.339 (t, 1H, ArH, *J* = 7.6 Hz), 7.364 (t, 1H, ArH, *J* = 7.6 Hz), 7.656 (d, 1H, ArH, *J* = 4.4 Hz), 7.667 (d, 1H, ArH, *J* = 4.4 Hz), 7.963 (d, 1H, ArH, *J* = 6.4 Hz), 8.095 (s, 1H, CH), 9.980(s, 1H, NH).13C NMR (δ ppm, DMSO-d6): 26.888, 48.884, 58.506, 110.754, 115.825, 116.016, 118.851, 123.994, 124.858, 125.137, 125.751, 125.827, 126.235, 126.347, 141.715, 151.835, 154.863, 164.130, 165.152, 197.927.MS (m/z): M + 1 analysed 426.10, M + 1 predicted 426.44.

##### 4–(4-((Benzo[d]oxazol-2-ylthio)methyl)-1H-1,2,3-triazol-1-yl)-N-(3-chlorophenyl)-3-oxobutanamide(3d) (see Fig. S10,S11,S12):

Light brown solid, yield: 92%; m.p.: 165 °C–169 °C; FTIR νmax (KBr, cm^−1^): 3347.80 (N–H str.), 3124.80 (C–H str., triazole ring), 2881.22 (C–H str., al), 1718.10 (C = O), 1665.10 (C = O str.,amide), 1542.10, 1481.18 (C = C str., Ar), 692.33 (C–S str.) cm^−1^. 1H NMR (δ ppm, DMSO-d6): 3.740 (s, 2H, CH_2_), 4.726 (s, 2H, CH_2_), 5.604 (s, 2H, CH_2_), 7.148 (d, 1H, ArH, *J* = 7.6 Hz), 7.350 (d, 1H, ArH, *J* = 8.0 Hz), 7.370 (t, 1H, ArH, *J* = 8.0 Hz), 7.382 (t, 2H, ArH, *J* = 4.8 Hz), 7.402 (t, 1H, ArH, *J* = 8.0 Hz), 7.656 (d, 1H, ArH, *J* = 8.2 Hz), 7.677 (d, 1H, ArH, *J* = 8.4 Hz), 7.799 (s, 1H, CH), 8.082 (s, 2H, CH_2_), 10.357 (s, 1H, NH).13C NMR (δ ppm, DMSO-d6): 26.885, 49.292, 58.608, 110.750, 118.007, 118.851, 119.119, 123.800, 124.864, 125.135, 131.007, 133.584, 140.531, 141.691, 151.823, 164.184, 165.064, 197.711. MS (m/z): M + 1 analysed 442.07, M + 1 predicted 442.44.

##### 4–(4-((Benzo[d]oxazol-2-ylthio)methyl)-1H-1,2,3-triazol-1-yl)-3-oxo-N-(p-tolyl) butanamide(3e) (see Fig. S13,S14,S15):

Light brown solid, yield: 91%; m.p.: 175 °C–179 °C; FTIR νmax (KBr, cm^−1^): 3317.70(N–H str.), 3164.40 (C–H str., triazole ring), 2901.22 (C–H str.,al), 1716.19 (C = O), 1654.50 (C = O str.,amide), 1531.20, 1467.04 (C = C str., Ar), 690.03 (C–S str.) cm^−1^. 1H NMR (δ ppm, DMSO-d6): 2.245 (s, 3H, CH_3_), 3.715 (s, 2H, CH_2_), 4.723 (s, 2H, CH_2_), 5.606 (s, 2H, CH_2_),7.105 (d, 2H, ArH, *J* = 8.4 Hz), 7.324 (t, 1H, ArH, *J* = 7.2 Hz), 7.338 (t, 1H, ArH, *J* = 7.2 Hz), 7.441 (d, 2H, ArH, *J* = 8.4 Hz), 7.460 (d, 1H, ArH, *J* = 6.4 Hz), 7.662(d, 1H, ArH, *J* = 6.4 Hz), 8.085(s, 1H, CH), 10.091(s, 1H, NH).13C NMR (δ ppm, DMSO-d6): 20.889, 26.849, 49.238, 58.656, 110.726, 118.837, 119.673, 120.272, 124.863, 125.124, 125.871, 129.622, 133. 058, 136.612, 141.663, 151.798, 164.213, 164.375, 197.961. MS (m/z): M + 1 analysed 422.13, M + 1 predicted 415.13.

##### 4–(4-((Benzo[d]oxazol-2-ylthio)methyl)-1H-1,2,3-triazol-1-yl)-3-oxo-N-(4-(trifluoromethyl) phenyl)butanamide (3f) (see Fig. S16,S17,S18):

Light brown solid, yield: 84%; m.p.: 185 °C–189 °C; FTIR νmax (KBr, cm^−1^): 3417.50(N–H str.), 3214.10 (C–H str., triazole ring), 2907.39 (C–H str.,al), 1740.17 (C = O), 1683.20 (C = O str.,amide), 1537.30, 1492.10 (C = C str., Ar), 691.13 (C–S str.) cm^−1^. 1H NMR (δ ppm, DMSO-d6): 3.778 (s, 2H, CH_2_), 4.718 (s, 1H, CH_2_), 5.603 (s, 2H, CH_2_), 7.346 (d, 2H, ArH, *J* = 7.6 Hz), 7.375 (d, 2H, ArH, *J* = 7.6 Hz), 7.664 (t, 1H, ArH, *J* = 7.2 Hz), 7.705 (t, 1H, ArH, *J* = 7.2 Hz), 7.768 (d, 1H, ArH, *J* = 8.2 Hz), 7.790 (d, 1H, ArH, *J* = 8.4 Hz), 8.064 (s, 1H, CH), 10.536 (s, 1H, NH).13C NMR (δ ppm, DMSO-d6): 26.885, 49.354, 58.582, 110.746, 118.845, 119.534, 124.835, 124.602, 125.123, 125.717, 126.596, 126.633, 141.730, 142.688, 142.773, 151.843, 164.101, 165.349, 197.721. MS (m/z): M + 1 analysed 476.10, M + 1 predicted 476.09.

##### 4–(4-((Benzo[d]oxazol-2-ylthio)methyl)-1H-1,2,3-triazol-1-yl)-N-(4-chlorophenyl)-3-oxo butanamide(3g) (see Fig. S19,S20,S21):

Light brown solid, yield: 89%; m.p.: 171 °C–174 °C; FTIR νmax (KBr, cm^−1^): 3350.41(N–H str.), 3129.30 (C–H str., triazole ring), 2931.42 (C–H str.,al), 1721.11 (C = O), 1639.84 (C = O str.,amide), 1541.10, 1487.10 (C = C str., Ar), 689.49 (C–S str.) cm^−1^. 1H NMR (δ ppm, DMSO-d6): 3.728 (s, 2H, CH_2_), 4.720 (s, 2H, CH_2_), 5.597 (s, 2H, CH_2_), 7.361 (t, 1H, ArH, *J* = 6.8 Hz), 7.378 (t, 1H, ArH, *J* = 6.8 Hz), 7.381 (d, 2H, ArH, *J* = 8.0 Hz), 7.577 (d, 1H, ArH, *J* = 8.0 Hz), 7.597 (d, 2H, ArH, *J* = 8.0 Hz), 7.665 (d, 1H, ArH, *J* = 8.0 Hz), 8.086 (s, 1H, CH), 10.303 (s, 1H, NH).13C NMR (δ ppm, DMSO-d6): 26.846, 49.237, 58.644, 110.746, 118.838, 121.206, 124.892, 125.141, 126.078, 127.647, 129.181, 138.049, 140.052, 141.643, 151.788, 164.269, 164.798, 197.767. MS (m/z): M + 1 analysed 442.09, M + 1 predicted 442.06.

##### 4–(4-((Benzo[d]oxazol-2-ylthio)methyl)-1H-1,2,3-triazol-1-yl)-N-(4-bromophenyl)-3-oxo butanamide(3h) (see Fig. S22,S23,S24):

Light brown solid, yield: 82%; m.p.: 210 °C–214 °C; FTIR νmax (KBr, cm^−1^): 3367.67(N–H str.), 3224.10 (C–H str., triazole ring), 2969.02 (C–H str.,al), 1780.06 (C = O), 1671.42 (C = O str.,amide), 1567.51, 1482.24 (C = C str., Ar), 688.40 (C–S str.) cm^−1^. 1H NMR (δ ppm, DMSO-d6): 3.725 (s, 2H, CH_2_), 4.717 (s, 2H, CH_2_), 5.591 (s, 2H, CH_2_), 7.349 (d, 2H, ArH, *J* = 7.4 Hz), 7.357 (t, 1H, ArH, *J* = 6.6 Hz), 7.374 (t, 1H, ArH, *J* = 6.6 Hz), 7.521 (d, 2H, ArH, *J* = 7.4 Hz), 7.662 (d, 1H, ArH, *J* = 4.8 Hz), 7.674 (d, 1H, ArH, *J* = 5.0 Hz), 8.064 (s, 1H, CH), 10.292 (s, 1H, NH). 13C NMR (δ ppm, DMSO-d6): 26.896, 49.281, 58.633, 110.733, 115.669, 118.827, 121.573, 122.912, 123.852, 124.858, 125.122, 132.087, 138.481, 141.671, 151.810, 164.206, 164.824, 197.762. MS (m/z): M + 1 analysed 486.04, M + 1 predicted 486.34.

##### 4–(4-((Benzo[d]oxazol-2-ylthio)methyl)-1H-1,2,3-triazol-1-yl)-N-(4-methoxyphenyl)-3-oxo butanamide(3i) (see Fig. S25,S26,S27):

Light brown solid, yield: 87%; m.p.: 169 °C–172 °C; FTIR νmax (KBr, cm^−1^): 3347.55(N–H str.), 3124.22 (C–H str., triazole ring), 2869.12 (C–H str.,al), 1712.30 (C = O), 1653.41 (C = O str.,amide), 1537.39, 1491.14 (C = C str., Ar), 682.73 (C–S str.) cm^−1^. 1H NMR (δ ppm, DMSO-d6): 3.685 (s, 3H, CH_3_), 3.720 (s, 2H, CH_2_), 4.717 (s, 2H, CH_2_), 5.594 (s, 2H, CH_2_), 6.901(d, 2H, ArH, *J* = 8.4 Hz), 7.344 (t, 1H, ArH, *J* = 7.2 Hz), 7.458 (d, 2H, ArH, *J* = 8.4 Hz), 7.480 (t, 1H, ArH, *J* = 7.2 Hz), 7.656 (d, 1H, ArH, *J* = 6.4 Hz), 7.671 (d, 1H, ArH, *J* = 6.4 Hz), 8.076 (s, 1H, CH), 10.303(s, 1H, NH).13C NMR (δ ppm, DMSO-d6): 26.876, 49.135, 55.623, 58.637, 110.746, 112.493, 114.367, 118.846, 121.241, 124.871, 125.140, 130.644, 132.248, 141.676, 151.814, 155.910, 164.086, 164.185, 197.992. MS (m/z): M + 1 analysed 438.13, M + 1 predicted 438.47.

##### 4–(4-((Benzo[d]oxazol-2-ylthio)methyl)-1H-1,2,3-triazol-1-yl)-N-(4-ethoxyphenyl)-3-oxo butanamide(3j) (see Fig. S28,S29,S30):

Light brown solid, yield: 93%; m.p.: 175 °C–179 °C; FTIR νmax (KBr, cm^−1^): 3357.60 (N–H str.), 3134.20 (C–H str., triazole ring), 2871.32 (C–H str.,al), 1710.16 (C = O), 1643.70 (C = O str., amide), 1520.20, 1472.14 (C = C str., Ar), 687.43 (C–S str.) cm^−1^. 1H NMR (δ ppm, DMSO-d6): 1.305 (t, 3H, CH_3_), 3.682 (s, 2H, CH_2_), 3.987 (m, 2H, CH_2_), 4.716 (s, 2H, CH_2_), 5.591 (s, 2H, CH_2_), 6.884 (d, 2H, ArH, *J* = 8.4 Hz), 7.336 (t, 1H, ArH, *J* = 4.0 Hz), 7.346 (t, 1H, ArH, *J* = 4.0 Hz), 7.424 (d, 2H, ArH, *J* = 8.4 Hz), 7.468 (d, 1H, ArH, *J* = 8.4 Hz), 7.676 (d, 1H, ArH, *J* = 8.0 Hz), 8.066 (s, 1H, CH), 10.018 (s, 1H, NH).13C NMR (δ ppm, DMSO-d6): 15.120, 26.895, 49.165, 58.632, 63.555, 110.731, 114.886, 114.966, 118.824, 121.234, 124.849, 125.119, 125.832, 132.168, 141.685, 151.817, 155.181, 164.061, 164.177,197.988. MS (m/z): M + 1 analysed 452.15, M + 1 predicted 452.50.

##### 2–(4-((Benzo[d]oxazol-2-ylthio)methyl)-1H-1,2,3-triazol-1-yl)-1-phenylethan-1-one (3k) (see Fig. S31,S32,S33):

White solid, yield: 90%; m.p.: 135 °C–139 °C; FTIR νmax (KBr, cm^−1^): 3029.80 (C–H str., triazole ring), 2842.52 (C–H str.,al),1653.12 (C = O str.), 1552.12, 1469.31 (C = C str., Ar), 689.19 (C–S str.) cm^−1^. 1H NMR (δ ppm, DMSO-d6): 4.791 (s, 2H, CH_2_), 6.202 (s, 2H, CH_2_), 7.341 (t, 1H, ArH, *J* = 7.6 Hz), 7.593 (t, 2H, ArH, *J* = 7.6 Hz), 7.612 (t, 1H, ArH, *J* = 7.6 Hz), 7.659 (d, 1H, ArH, *J* = 2.8 Hz), 7.674 (d, 1H, ArH, *J* = 6.0 Hz), 7.726 (t, 1H, ArH, *J* = 7.6 Hz), 8.055 (d, 2H, ArH, *J* = 7.6 Hz), 8.212 (s, 1H, CH). 13C NMR (δ ppm, DMSO-d6): 26.795, 56.629, 110.772, 118.893, 125.017, 125.117, 126.370, 128.617, 129.435, 134.481, 134.727, 141.494, 143.385, 151.670, 164.626, 192.401. MS (m/z): M + 1 analysed 351.19, M + 1 predicted 351.39.

##### 2–(4-((Benzo[d]oxazol-2-ylthio)methyl)-1H-1,2,3-triazol-1-yl)-1–(4-methoxyphenyl)ethan-1-one (3 l) (see Fig. S34,S35,S36):

White solid, yield: 86%; m.p.: 148 °C–152 °C; FTIR νmax (KBr, cm^−1^): 3084.30 (C–H str., triazole ring), 2886.02 (C–H str.,al),1674.15 (C = O str.), 1582.42, 1474.31 (C = C str., Ar), 683.99 (C–S str.) cm^−1^. 1H NMR (δ ppm, DMSO-d6): 3.868 (s, 3H, CH_3_), 4.749 (s, 2H, CH_2_), 6.099 (s, 2H, CH_2_), 7.117 (d, 2H, ArH, *J* = 8.8 Hz), 7.330 (d, 1H, ArH, *J* = 2.8 Hz), 7.345 (d, 1H, ArH, *J* = 2.0 Hz), 7.359 (t, 1H, ArH, *J* = 7.2 Hz), 7.661 (t, 1H, ArH, *J* = 7.2 Hz), 8.035 (d, 2H, ArH, *J* = 8.8 Hz), 8.146 (s, 1H, CH).13C NMR (δ ppm, DMSO-d6): 26.963, 56.054, 56.144, 110.751, 114.668, 118.835, 124.859, 125.133, 126.132, 127.389, 131.023, 141.693, 142.795, 151.819, 164.209, 164.365, 190.739. MS (m/z): M + 1 analysed 381.19, M + 1 predicted 381.42.

##### 2–(4-((Benzo[d]oxazol-2-ylthio)methyl)-1H-1,2,3-triazol-1-yl)-1-(p-tolyl)ethan-1-one(3m) (see Fig. S37,S38,S39):

White solid, yield: 87%; m.p.: 142 °C–146 °C; FTIR νmax (KBr, cm^−1^): 3073.14 (C–H str., triazole ring), 2840.27 (C–H str.,al),1683.12 (C = O str.), 1544.32, 1445.19 (C = C str., Ar), 687.19 (C–S str.) cm^−1^. 1H NMR (δ ppm, DMSO-d6): 2.400 (s, 3H, CH_3_), 4.747 (s, 2H, CH_2_), 6.122 (s, 2H, CH_2_), 7.355 (t, 1H, ArH, *J* = 7.6 Hz),7.400 (d, 2H, ArH, *J* = 8.0 Hz), 7.657 (d, 1H, ArH, *J* = 7.8 Hz),7.678 (d, 1H, ArH, *J* = 7.4 Hz), 7.696 (t, 1H, ArH, *J* = 7.6 Hz),7.946 (d, 2H, ArH, *J* = 8.0 Hz), 8.154 (s, 1H, CH).13C NMR (δ ppm, DMSO-d6): 21.723, 26.870, 56.309, 110.757, 118.864, 124.914, 125.160, 126.141, 128.704, 128.921, 129.971, 132.010, 141.639, 145.349, 151.781, 164.274, 191.954. MS (m/z): M + 1 analysed 365.16, M + 1 predicted 365.43.

##### 2–(4-((Benzo[d]oxazol-2-ylthio)methyl)-1H-1,2,3-triazol-1-yl)-1–(4-fluorophenyl)ethan-1-one(3n) (see Fig. S40,S41,S42):

White solid, yield: 86%; m.p.: 145 °C–149 °C; FTIR νmax (KBr, cm^−1^): 3054.30 (C–H str., triazole ring), 2871.11 (C–H str.,al),1623.22 (C = O str.), 1526.12, 1459.84 (C = C str., Ar), 692.09 (C–S str.) cm^−1^. 1H NMR (δ ppm, DMSO-d6): 4.759 (s, 2H, CH_2_), 6.173 (s, 2H, CH_2_), 7.347 (t, 1H, ArH, *J* = 8.8 Hz), 7.443 (t, 1H, ArH, *J* = 8.8 Hz), 7.665 (d, 1H, ArH, *J* = 8.0 Hz), 7.668 (d, 1H, ArH, *J* = 8.0 Hz), 8.137 (d, 2H, ArH, *J* = 8.8 Hz), 8.150 (d, 2H, ArH, *J* = 8.4 Hz), 8.166 (s, 1H, CH).13C NMR (δ ppm, DMSO-d6): 26.923, 52.382, 110.729, 116.415, 116.634, 118.849, 124.855, 125.115, 126.102, 131.292, 131.320, 131.679, 131.775, 141.679, 151.806, 164.226, 164.763, 167.278, 191.182. MS (m/z): M + 1 analysed 369.17, M + 1 predicted 369.38.

##### 2–(4-((Benzo[d]oxazol-2-ylthio)methyl)-1H-1,2,3-triazol-1-yl)-1–(4-nitrophenyl)ethan-1-one (3o) (see Fig. S43,S44,S45):

Light pink solid, yield: 81%; m.p.: 142 °C–146 °C; FTIR νmax (KBr, cm^−1^): 3341.20 (C–H str., triazole ring), 2891.16 (C–H str.,al),1632.62 (C = O str.), 1534.52, 1478.21 (C = C str., Ar), 681.4 9(C–S str.) cm^−1^. 1H NMR (δ ppm, DMSO-d6): 4.753 (s, 2H, CH_2_), 6.246 (s, 2H, CH_2_), 7.354 (t, 1H, ArH, *J* = 3.2 Hz), 7.384 (t, 1H1H, ArH, *J* = 3.2 Hz), 7.671 (d, 1H, ArH, *J* = 7.6 Hz), 7.691 (d, 1H, ArH, *J* = 7.2 Hz), 8.143 (s, 1H, CH), 8.286 (d, 2H, ArH, *J* = 9.2 Hz), 8.416 (d, 2H, ArH, *J* = 8.6 Hz).13C NMR (δ ppm, DMSO-d6): 26.927, 56.810, 110.758, 118.851, 124.432, 124.864, 125.141, 125.949, 129.701, 130.112, 139.216, 141.713, 150.886, 151.837, 164.144, 191.988. MS (m/z): M + 1 analysed 396.16, M + 1 predicted 396.39.

### Biological evaluation

#### Determination of MIC using REMA method

To begin, master solutions of each compound were prepared at a concentration of 10,000 μg/mL using DMSO to ensure sterility. Subsequently, a dilution plate was set up from these master solutions, achieving a final compound concentration of 100 μg/mL. Next, a 96-well plate was set up for MIC determination. In every well, 100 μL of Middlebrook 7H9 broth enriched with 10% oleic acid, albumin, dextrose, and catalase was dispensed, except for the control wells. In the compound control, 150 μL was added, and in the medium control, 200 μL was added^37^. Each compound was added in 100 μL volumes, undergoing serial dilutions. Concurrently, a suspension of MTB H37Rv (ATCC 27294) was prepared to achieve a density of 3 × 10^6^ CFU/mL. From this mixture, 100 μL was dispensed into every well of the 96-well plate, excluding control wells, establishing an initial concentration of 25 μg/mL for each compound. Following this, the plates were placed in a 5% CO_2_ atmosphere and incubated at 37 °C for 7 days. After incubation, 30 μL of 0.01% resazurin, dissolved in sterile distilled water, was added to every well. Following a 24-h incubation, fluorescence intensity was measured using the Biotek^®^ Synergy H1 device. The MIC90, representing the concentration causing a 90% reduction in bacterial growth, was derived from these measurements. This assay was conducted in triplicate, and the reported result represents the average of the three independent trials^38^^,^^39^.

#### Cytotoxicity assay

Mouse fibroblast 3T3 cells (ATCC CRL-1658) were seeded at a density of 2 × 10^5^ cells/mL in a 96-well plate. They were cultured overnight in DMEM medium supplemented with 10% FBS and penicillin/streptomycin (100 units/mL). The cells were maintained under 5% CO_2_ at 37 °C^29^. Following a 24-h incubation period, the existing media were replaced. The cells were then exposed to different concentrations of the test compounds. Subsequently, they underwent an additional 24 h incubation. After this incubation period, the cells were washed. The MTT solution was added to the plate and incubated for 4 h. After incubation, dimethyl sulfoxide (100 µL) was added. This addition was maintained for 15 min at room temperature to dissolve formazan crystals. Subsequently, the absorbance at 540 nm was recorded using a microplate reader (BioTEK, USA)^24^. The IC_50_, representing the drug concentration (µM) causing cytotoxicity in 50% of the cells, was calculated accordingly.

#### DprE1 redox indicator assay

A redox indicator assay was conducted following a previously described protocol, utilising the reduction of resazurin to its fluorescent form, resorufin. In this assay, the DprE1 cofactor FAD undergoes reduction to FADH_2_, while the C-2 hydroxyl group of the substrate GGPR is oxidised to form a keto-intermediate known as geranylgeranylphosphoryl-β-d-2′-keto-erythro-penta-furanose (GGPX)^11^^,^^40^. The inhibition of DprE1 was assessed using an activity assay, with the half maximal inhibitory concentration (IC_50_) determined for each tested compound. Intermediate plates of compounds were prepared in V-bottomed 96-well plates, where the compounds were serially diluted twofold in DMSO. Subsequently, 1 µL of each compound dilution was pipetted in triplicate into Greiner black-bottomed 384 plates. The master mix (19 µL), comprising 5 µM DprE1, 50 mM Hepes pH 7.5, 100 mM NaCl, and 100 µM resazurin, was added to the compounds in the plate. The assay was initiated with 5 µL of 1 mM GGPR using the pump on the POLARstar Omega plate reader (BMG Labtech). Emission was measured at 590 nm with excitation at 530 nm at 37 °C^41^^.^ IC_50_ values were calculated using Prism GraphPad, fitting the data to a four-parameter dose–response curve. The initial rates of activity were used for the calculations, and fluorescence units were converted to µM resazurin reduced by referencing a resorufin standard curve.

### Exploration of molecular interaction via molecular docking analysis

Utilising computational methods, docking serves as a valuable tool for investigating intermolecular interactions, with CDOCKER emerging as a prominent algorithm in this domain. CDOCKER employs a simulated annealing-based approach and relies on the CHARMm force field for structural representation^24^^,^^42^. The three-dimensional (3D) X-ray crystal structure of the DprE1 protein (PDB ID: 4KW5) was initially obtained from the protein structure database (http://www.rcsb.org). The structure had a resolution of 2.61 Å. Subsequent to this, a protein preparation protocol was applied to rectify structural irregularities, including side chain corrections, loop region adjustments, and conformations. Meanwhile, ligands underwent processing to ensure correct chemical valences and charges. This step involved using the CHARMm all-atom force field^23^. Identification of the catalytic site for ligand binding on the target receptor was facilitated through a receptor cavity search. This search utilised the flood-filling algorithm and specifically focused on the DprE1 binding region, known as the catalytic site. Subsequently, the prepared receptor PDB and optimised ligand structure files underwent docking using the CDOCKER algorithm to unravel the molecular interactions. The potential binding site was identified with a volume of 1630.6 Å^3^ and a point count of 7066, using an equally spaced grid of 0.5 Å in the *X*, *Y*, and *Z* directions^23^^,^^25^. The designed compounds were subjected to docking at a specified spherical site with coordinates 13.72 (*X*), −19.10 (*Y*), and 36.52 (*Z*). Conformations were randomly generated through 1000 dynamic steps with default simulation annealing. The interactions of the ligands in their docked poses were analysed, and subsequently, these interactions were considered for further MD simulations for a more in-depth examination.

### Assessment of stability in protein–ligand complex using MD simulation

Through an MD simulation experiment, dynamic stability across a time span was verified for the static pose of the protein–ligand complex extracted from the molecular docking investigation. Further utilising energy graphs to visualise the relative stability, MD modelling was applied to natural protein structures in innate and docked complexes with ligands and pre-existing cofactor. To execute the simulations, GROMACS 2023 version on Ubuntu platform and other web-based servers were utilised^43^. The protein and ligand topology files were constructed using the CHARMm27 force field. This process was conducted either externally via the SwissParam online server (http://www.swissparam.ch/) or internally using the native GROMACS platform. The innate and complex protein systems were virtually grouped in a triclinic box, with a distance cut-off of 1.0 nm separating the protein’s outer surface from the box’s edges and conserving Van der Waal interactions. The partial Mesh Ewald summation method, with a 1.0 nm cut-off, was utilised to compute coulombic interactions for long-range electrostatics. The system was explicitly solvated using the TIP3P water model, maintaining periodic boundary conditions. Next, the system’s electro-neutrality was maintained by supplying the needed counter ions (Na^+^/Cl^-^)^44^. Using the steepest descent algorithm, the whole system’s energy was sequentially minimised over 5000 steps, with a tolerance of 1000 KJ mol^−1^ nm^–1^. Position constraints were applied to the complex to equilibrate the system. Canonical NVT and NPT ensembles were used to run 200 ps simulations at a constant temperature of 300 K and 1 bar of pressure. The initial velocities were generated following the Maxwell distribution. Temperature coupling was performed using velocity rescaling with a coupling constant of 0.1 ps. Temperature–pressure coupling was conducted using an extended ensemble Parrinello–Rahman algorithm, with a coupling constant of 2 ps. Consequently, a 300-ns MD production run with a 2-fs time step integration was applied to the equilibrated system. Every 500 steps, the trajectories were saved, the default GROMACS analytic tools were used for analysis, and the XMGRACE-5.1.22 program was used to visualise the data (http://plasma-gate.weizmann.ac.il/Grace/)^29^^,^^45^.

### Computational studies on ADMET and drug-likeness

Using the SwissADME web tool, various pharmacokinetic parameters for the top compounds were predicted. These include molecular weight (MW), TPSA, octanol-water partition coefficient (iLOGP), molecular refractivity (MR), aqueous solubility (ESOL LogS), cytochrome-P450 enzyme inhibition, GI absorption, bioavailability score, skin permeation (Log Kp), Lipinsky violations, and PAINS alerts.

## Supplementary Material

Supplementary_data.pdf

## Data Availability

The authors confirm that the data supporting the findings of this study are available within the article [and/or] its supplementary materials.
