# Supplementary material for: Novel hybrids of 1,2,3-triazole-benzoxazole: design, synthesis, and assessment of DprE1 enzyme inhibitors using fluorometric assay and computational analysis
Source: J Enzyme Inhib Med Chem. 2024 Sep 27;39(1):2403744. doi: 10.1080/14756366.2024.2403744 (PMC11441021; doi:10.1080/14756366.2024.2403744)
Supplement: Supplementary_data.pdf [file IENZ_A_2403744_SM3479.pdf]

COMPOUND 3a (BOK-1) SPECTRAL DATA

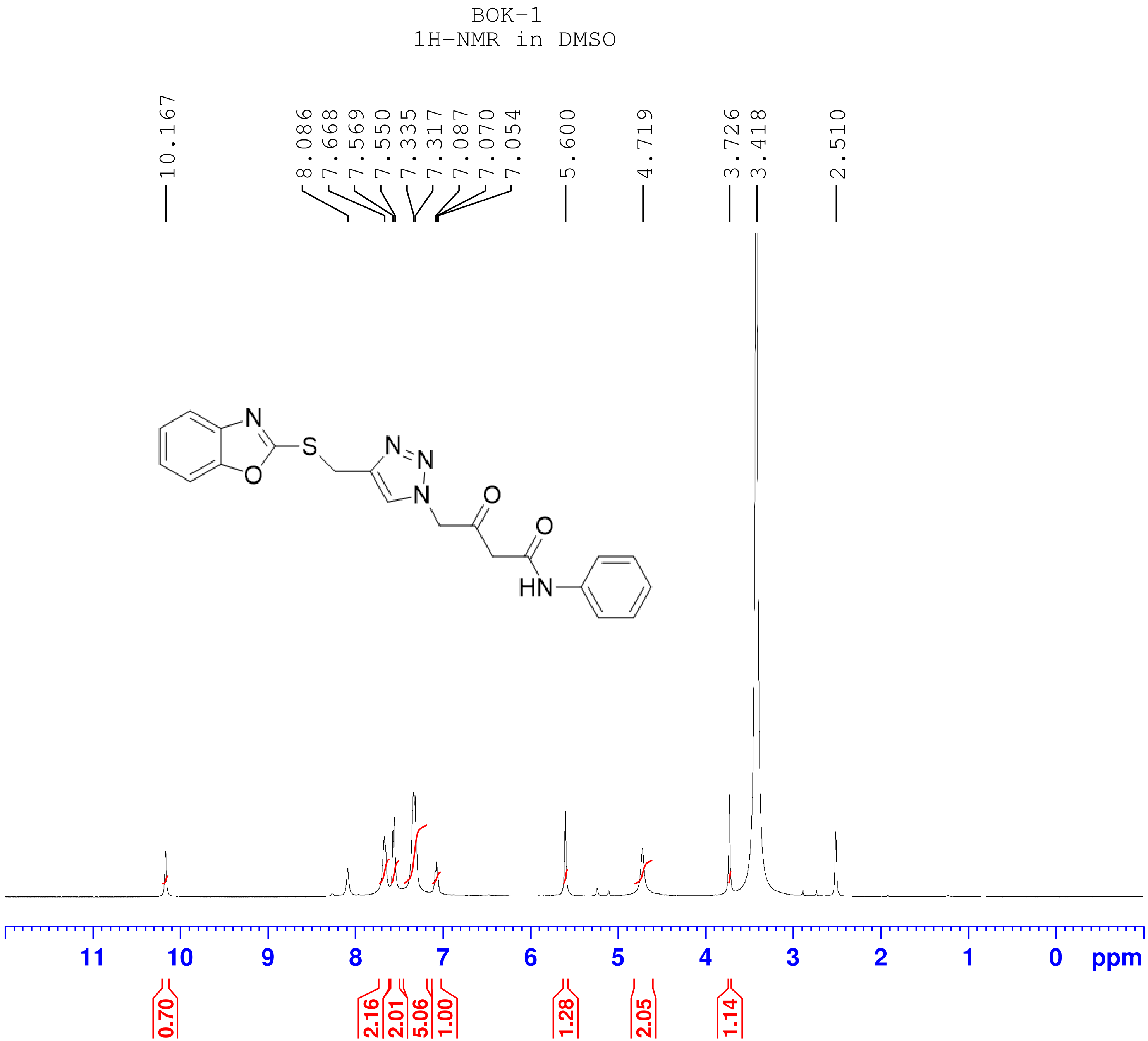

Current Data Parameters
NAME 23000786-BO-AN
EXPNO 1
PROCNO 1

F2 - Acquisition Parameters
Date_ 20230801
Time 8.21 h
INSTRUM spect
PROBHD Z108618_0984 (
PULPROG zg30
TD 65536
SOLVENT DMSO
NS 16
DS 2
SWH 8012.820 Hz
FIDRES 0.244532 Hz
AQ 4.0894465 sec
RG 100.18
DW 62.400 usec
DE 17.09 usec
TE 297.2 K
D1 1.00000000 sec
TD0 1
SFO1 400.3124719 MHz
NUC1 1H
P0 4.67 usec
P1 14.00 usec
PLW1 11.28999996 W

F2 - Processing parameters
SI 65536
SF 400.3100000 MHz
WDW EM
SSB 0
LB 0.30 Hz
GB 0
PC 1.00

^{1}H NMR spectrum of 4-((benzo[d]oxazol-2-ylthio)methyl)-1H-1,2,3-triazol-1-yl)-3-oxo-N-phenyl butanamide **3a (BOK-1)**

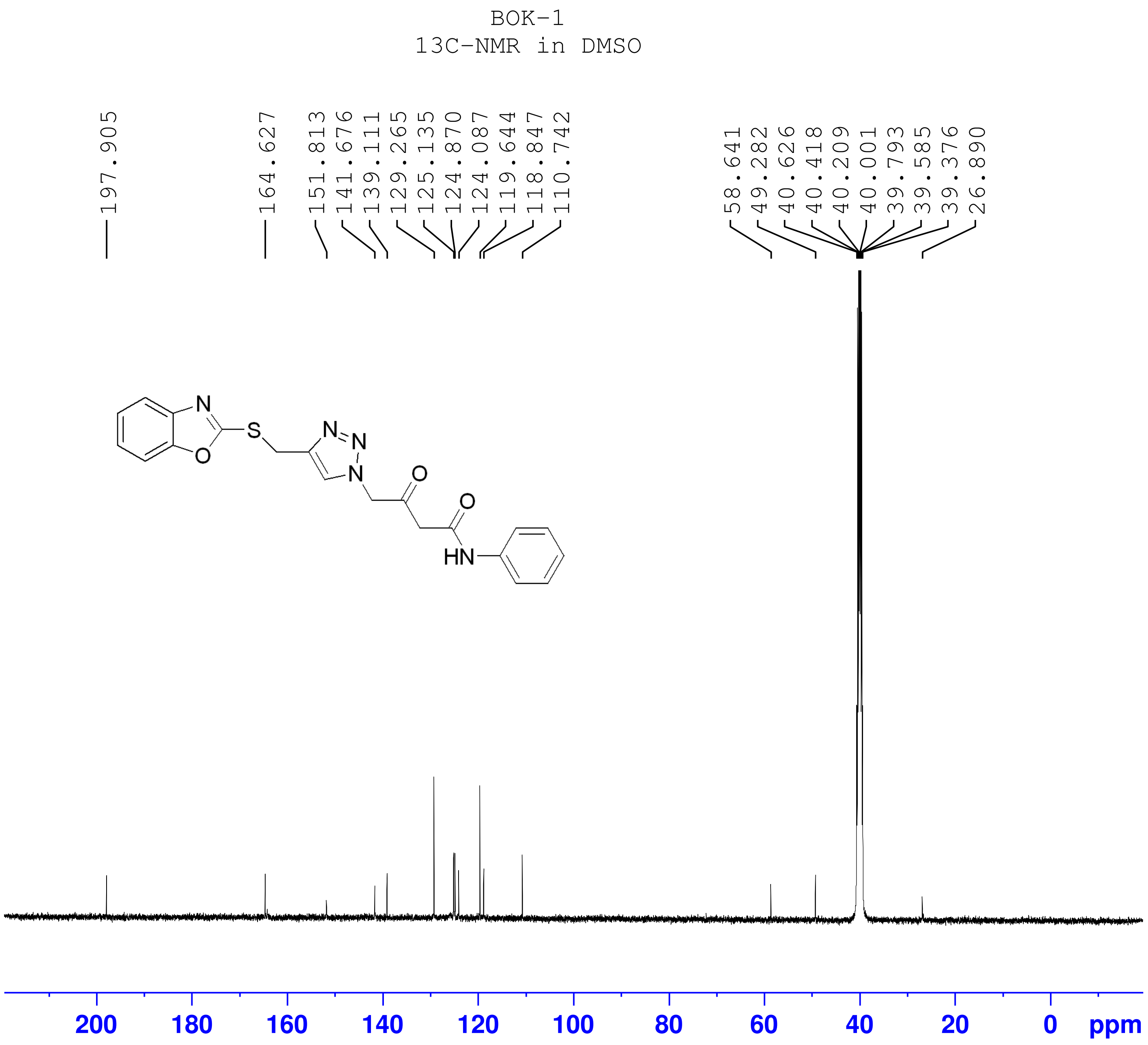

BRUKER

Current Data Parameters
NAME 23000786-BO-AN
EXPNO 2
PROCNO 1

F2 - Acquisition Parameters

Date_	20230801	
Time	9.03	h
INSTRUM	spect	
PROBHD	Z108618_0984 (	
PULPROG	zgpg30	
TD	65536	
SOLVENT	DMSO	
NS	1024	
DS	4	
SWH	24038.461	Hz
FIDRES	0.733596	Hz
AQ	1.3631488	sec
RG	2050	
DW	20.800	usec
DE	6.50	usec
TE	298.2	K
D1	1.00000000	sec
D11	0.03000000	sec
TD0	1	
SFO1	100.6680954	MHz
NUC1	13C	
P0	3.33	usec
P1	10.00	usec
PLW1	51.02600098	W
SFO2	400.3116012	MHz
NUC2	1H	
CPDPRG[2	waltz65	
PCPD2	90.00	usec
PLW2	11.28999996	W
PLW12	0.27318001	W
PLW13	0.13741000	W

F2 - Processing parameters

SI	32768	
SF	100.6580296	MHz
WDW	EM	
SSB	0	
LB	1.00	Hz
GB	0	
PC	1.40	

^{13}C NMR spectrum of 4-((benzo[d]oxazol-2-ylthio)methyl)-1H-1,2,3-triazol-1-yl)-3-oxo-N-phenyl butanamide **3a (BOK-1)**

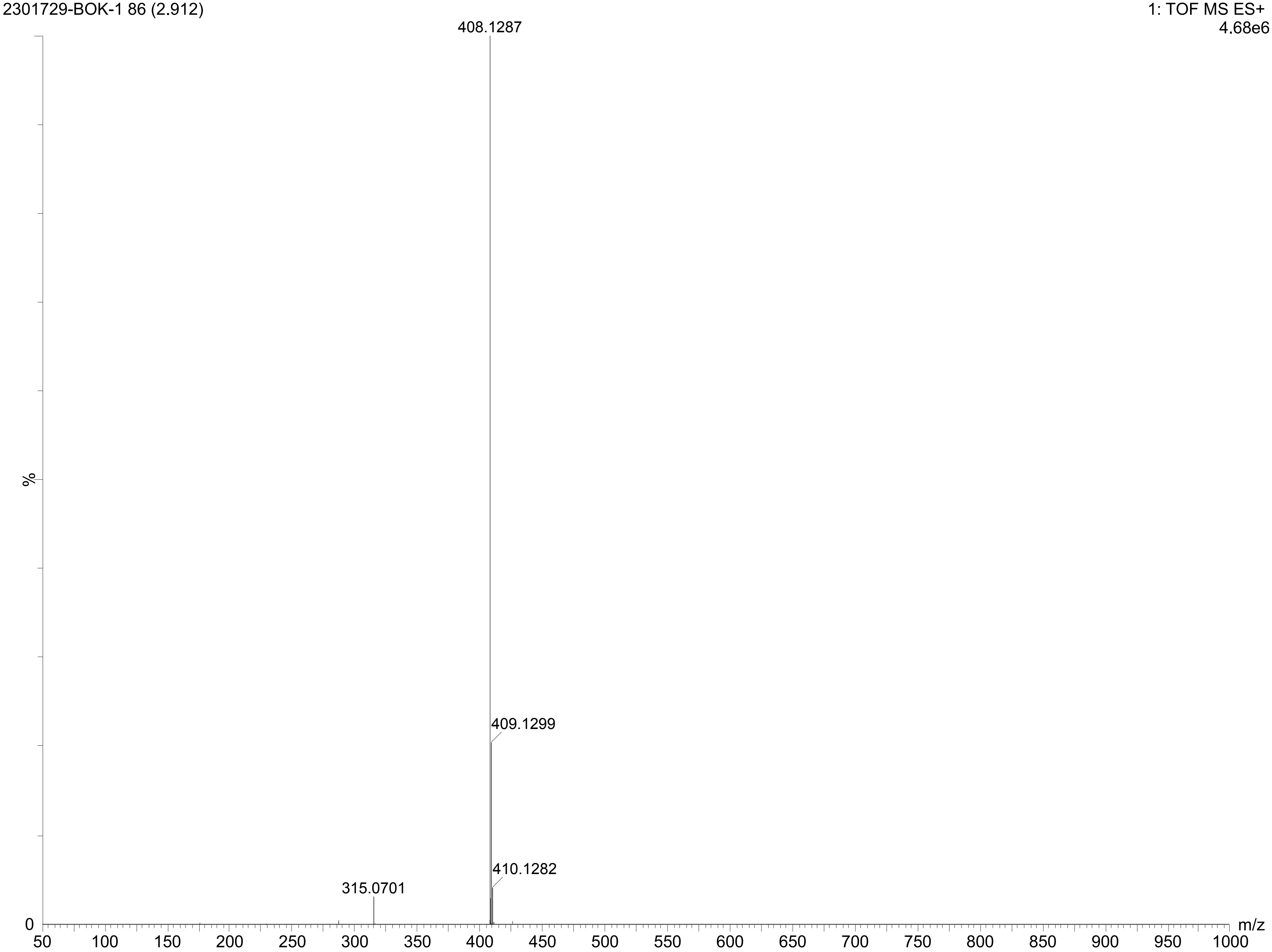

Mass spectrum of 4-((benzo[d]oxazol-2-ylthio)methyl)-1H-1,2,3-triazol-1-yl)-3-oxo-N-phenyl butanamide **3a (BOK-1)**

COMPOUND 3b (BOK-2) SPECTRAL DATA

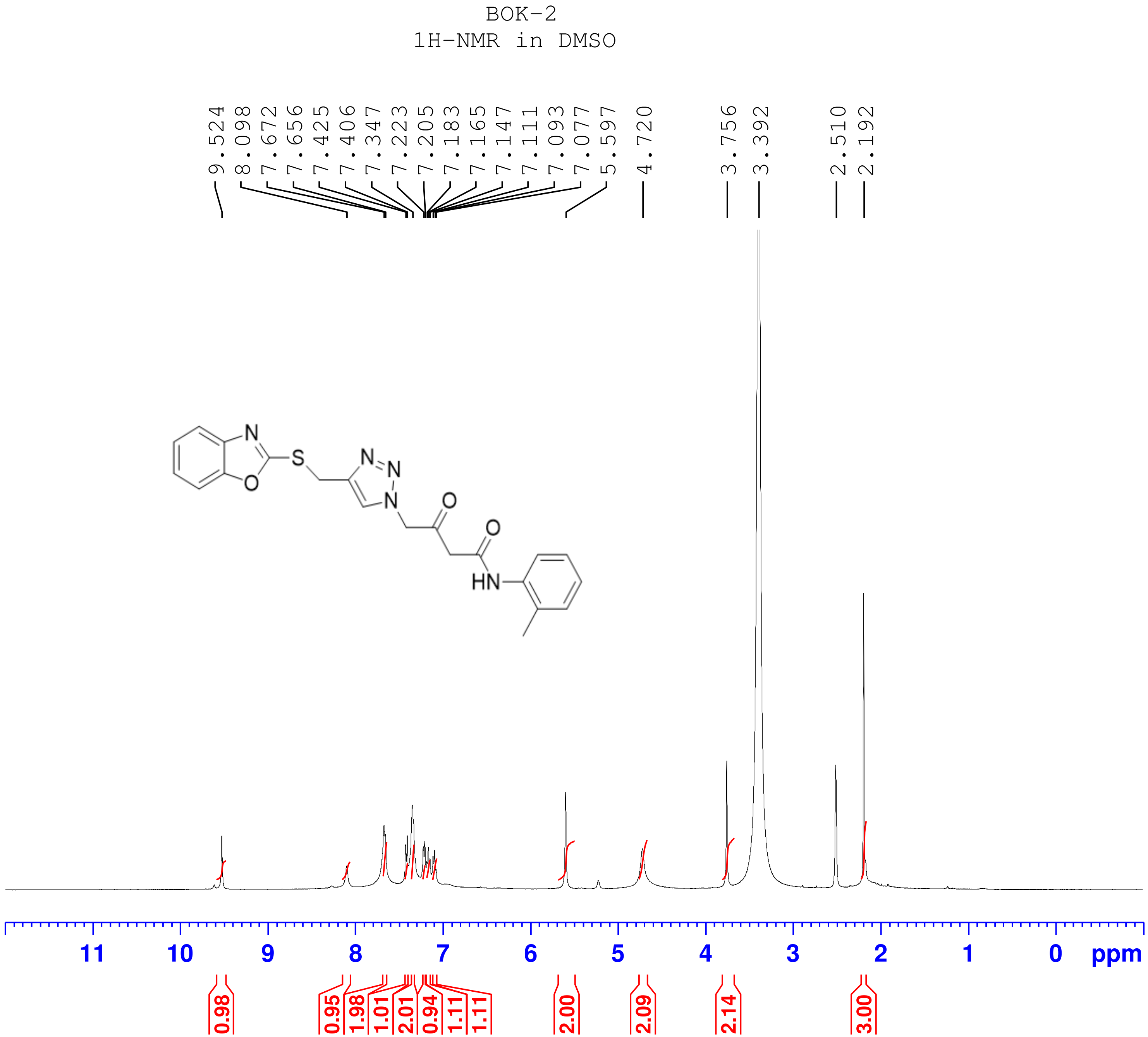

Current Data Parameters
NAME 23000780-BO-2CH3
EXPNO 10
PROCNO 1

F2 - Acquisition Parameters
Date_ 20230803
Time 10.03 h
INSTRUM spect
PROBHD Z108618_0984 (
PULPROG zg30
TD 65536
SOLVENT DMSO
NS 16
DS 2
SWH 8012.820 Hz
FIDRES 0.244532 Hz
AQ 4.0894465 sec
RG 32.13
DW 62.400 usec
DE 17.09 usec
TE 300.4 K
D1 1.00000000 sec
TD0 1
SFO1 400.3124719 MHz
NUC1 1H
P0 4.67 usec
P1 14.00 usec
PLW1 11.28999996 W

F2 - Processing parameters
SI 65536
SF 400.3100000 MHz
WDW EM
SSB 0
LB 0.30 Hz
GB 0
PC 1.00

^{1}H NMR spectrum of 4-(4-((benzo[d]oxazol-2-ylthio)methyl)-1H-1,2,3-triazol-1-yl)-3-oxo-N-(o-tolyl) butanamide 3b (BOK-2).

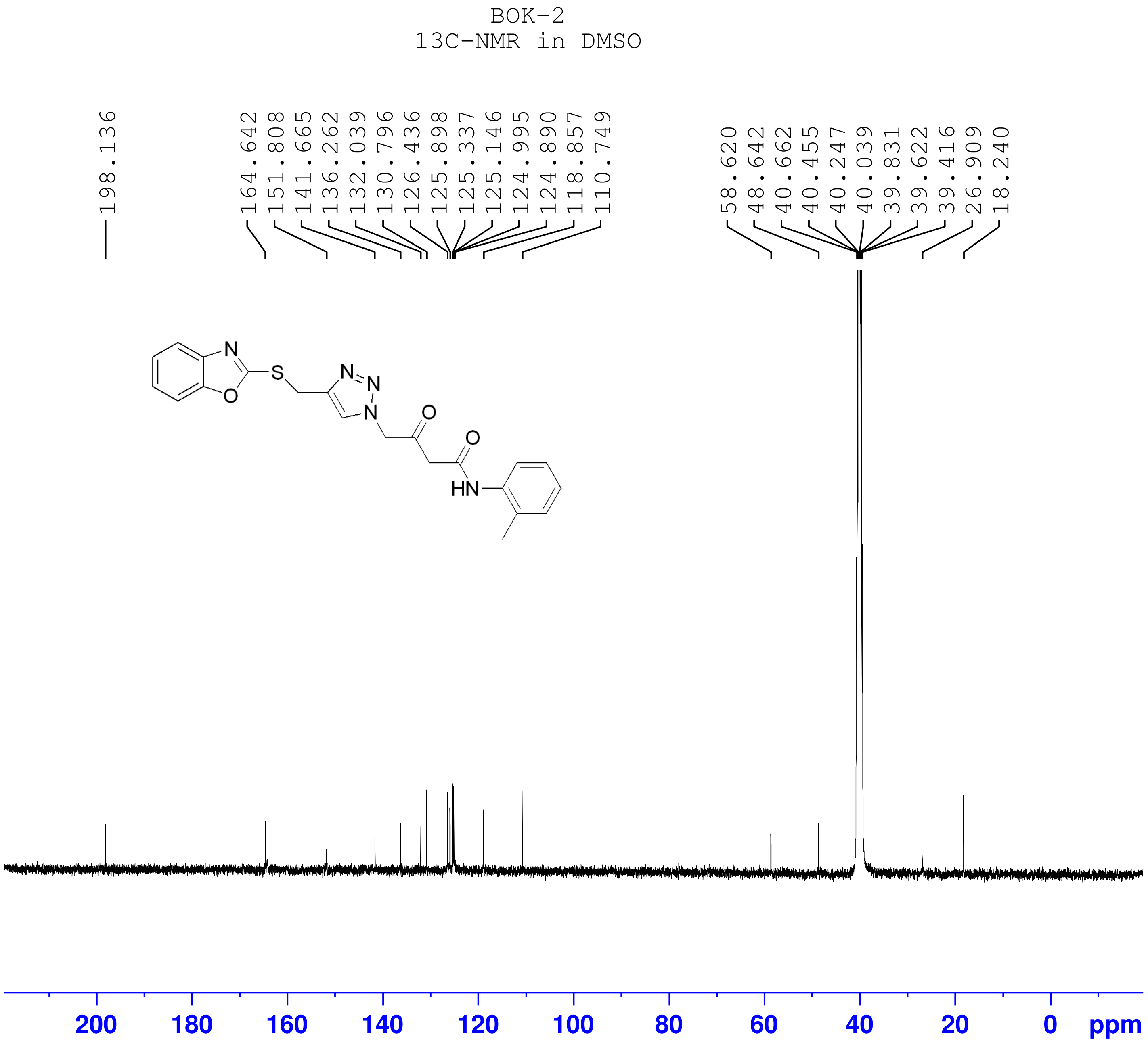

Current Data Parameters
NAME 23000780-BO-2CH3
EXPNO 2
PROCNO 1

F2 - Acquisition Parameters
Date_ 20230801
Time 5.07 h
INSTRUM spect
PROBHD Z108618_0984 (
PULPROG zgpg30
TD 65536
SOLVENT DMSO
NS 1024
DS 4
SWH 24038.461 Hz
FIDRES 0.733596 Hz
AQ 1.3631488 sec
RG 2050
DW 20.800 usec
DE 6.50 usec
TE 298.1 K
D1 1.00000000 sec
D11 0.03000000 sec
TD0 1
SFO1 100.6680954 MHz
NUC1 13C
P0 3.33 usec
P1 10.00 usec
PLW1 51.02600098 W
SFO2 400.3116012 MHz
NUC2 1H
CPDPRG[2 waltz65
PCPD2 90.00 usec
PLW2 11.28999996 W
PLW12 0.27318001 W
PLW13 0.13741000 W

F2 - Processing parameters
SI 32768
SF 100.6580296 MHz
WDW EM
SSB 0
LB 1.00 Hz
GB 0
PC 1.40

^{13}C NMR spectrum of 4-(4-((benzo[d]oxazol-2-ylthio)methyl)-1H-1,2,3-triazol-1-yl)-3-oxo-N-(o-tolyl) butanamide 3b (BOK-2).

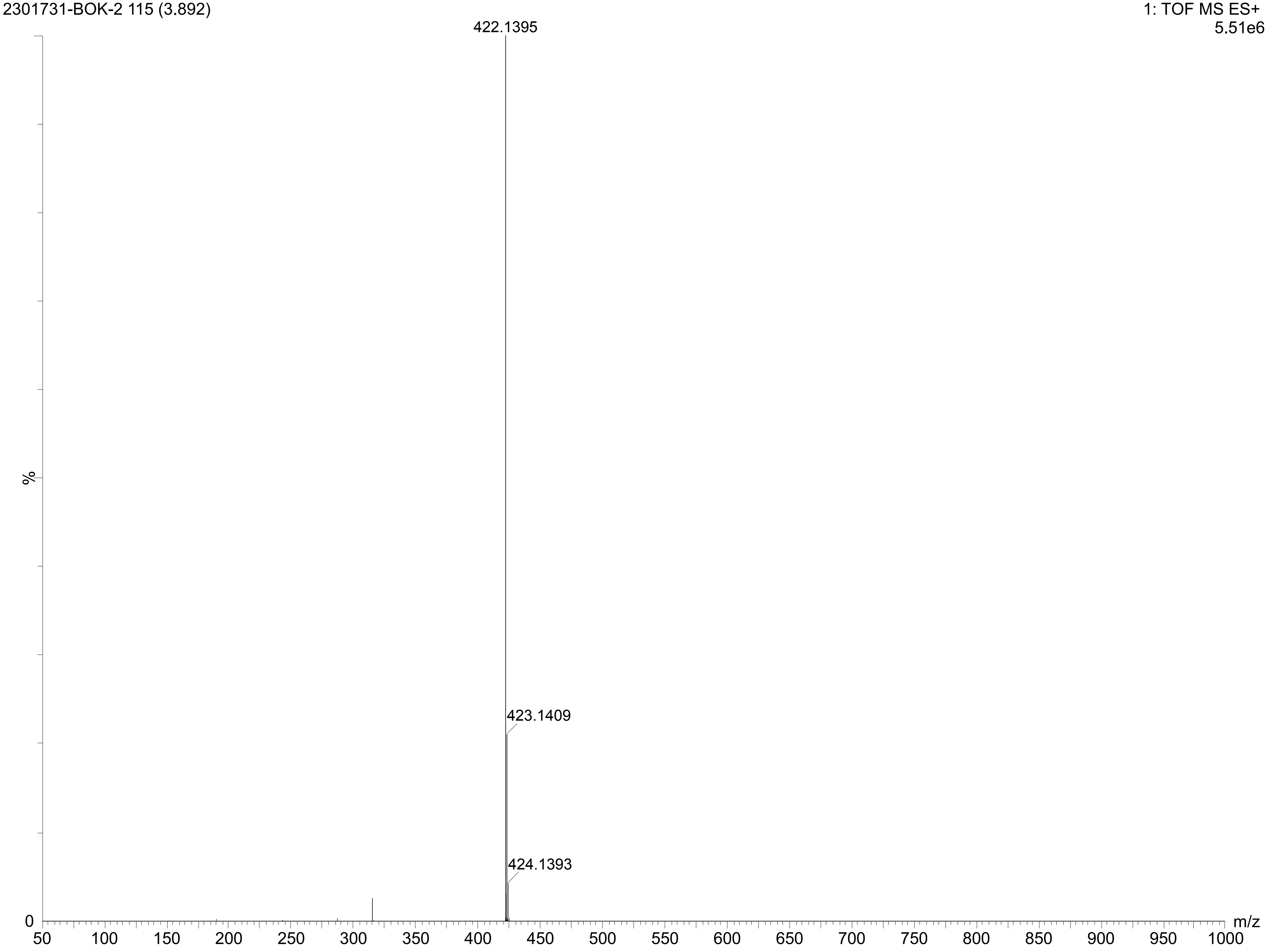

Mass spectrum of 4-(4-((benzo[d]oxazol-2-ylthio)methyl)-1H-1,2,3-triazol-1-yl)-3-oxo-N-(o-tolyl) butanamide 3b (BOK-2).

COMPOUND 3c (BOK-3) SPECTRAL DATA

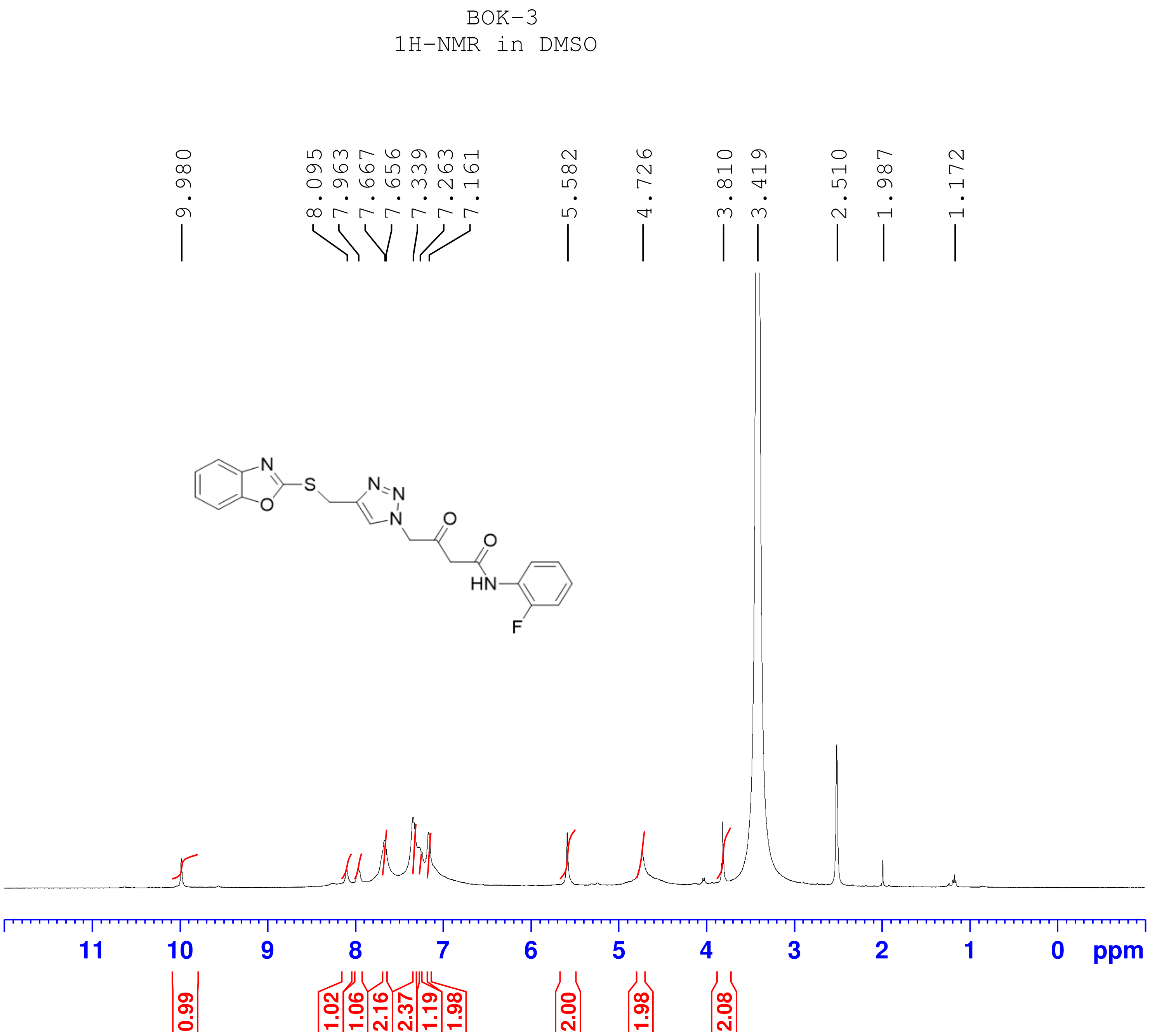

Current Data Parameters
NAME 23000784-BO-2F
EXPNO 1
PROCNO 1

F2 - Acquisition Parameters
Date_ 20230801
Time 7.30 h
INSTRUM spect
PROBHD Z108618_0984 (
PULPROG zg30
TD 65536
SOLVENT DMSO
NS 16
DS 2
SWH 8012.820 Hz
FIDRES 0.244532 Hz
AQ 4.0894465 sec
RG 114.18
DW 62.400 usec
DE 17.09 usec
TE 297.2 K
D1 1.00000000 sec
TD0 1
SFO1 400.3124719 MHz
NUC1 1H
P0 4.67 usec
P1 14.00 usec
PLW1 11.28999996 W

F2 - Processing parameters
SI 65536
SF 400.3100000 MHz
WDW EM
SSB 0
LB 0.30 Hz
GB 0
PC 1.00

^{1}H NMR spectrum of4-(4-((benzo[d]oxazol-2-ylthio)methyl)-1H-1,2,3-triazol-1-yl)-N-(2-fluorophenyl)-3-oxobutanamide **3c (BOK-3)**.

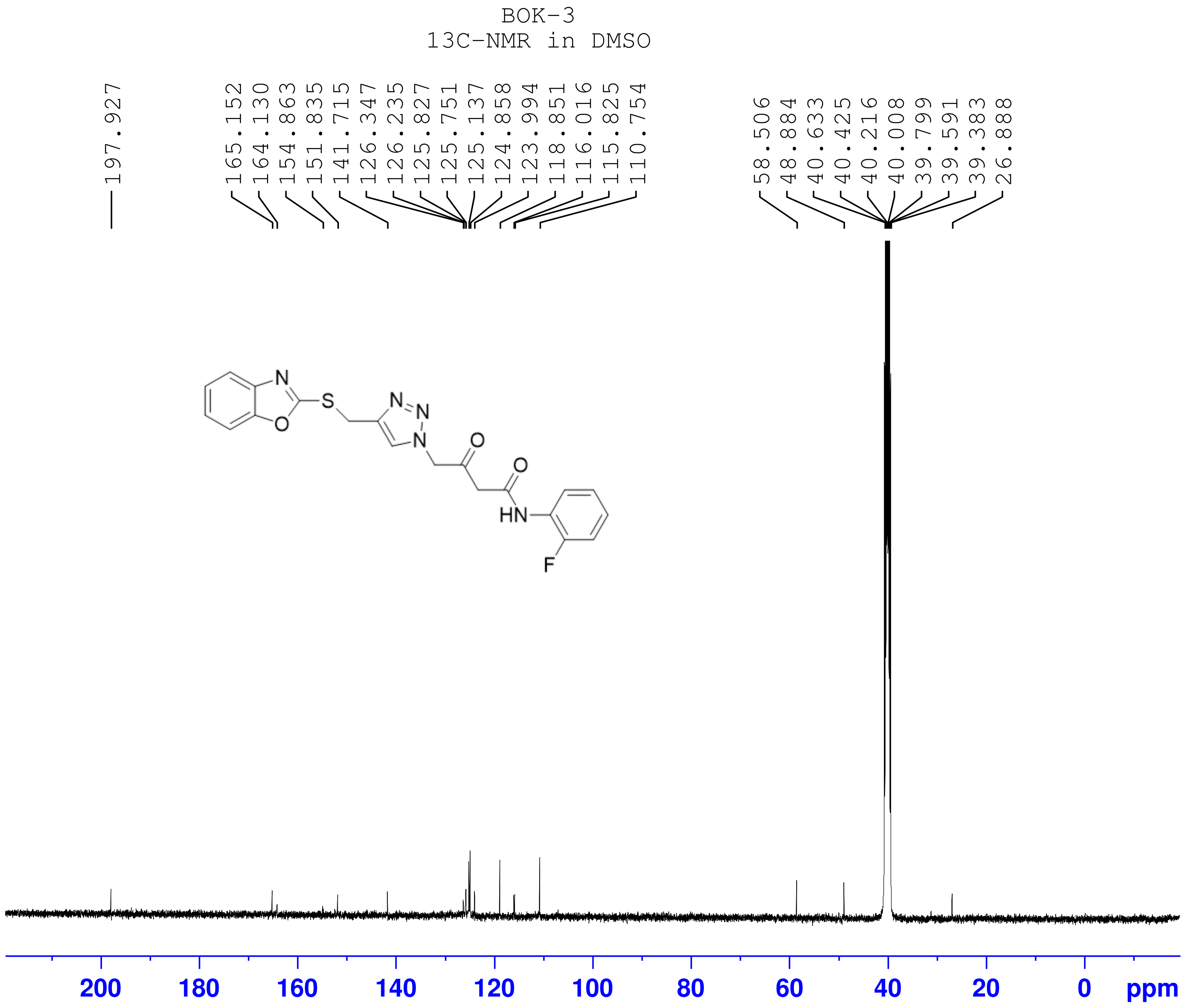

Current Data Parameters
NAME 23000847-BO-2F
EXPNO 2
PROCNO 1

F2 - Acquisition Parameters
Date_ 20230817
Time 21.11 h
INSTRUM spect
PROBHD Z108618_0984 (
PULPROG zgpg30
TD 65536
SOLVENT DMSO
NS 3000
DS 4
SWH 24038.461 Hz
FIDRES 0.733596 Hz
AQ 1.3631488 sec
RG 202.84
DW 20.800 usec
DE 6.50 usec
TE 298.1 K
D1 1.00000000 sec
D11 0.03000000 sec
TD0 1
SFO1 100.6680954 MHz
NUC1 13C
P0 3.33 usec
P1 10.00 usec
PLW1 51.02600098 W
SFO2 400.3116012 MHz
NUC2 1H
CPDPRG[2 waltz65
PCPD2 90.00 usec
PLW2 11.28999996 W
PLW12 0.27318001 W
PLW13 0.13741000 W

F2 - Processing parameters
SI 32768
SF 100.6580296 MHz
WDW EM
SSB 0
LB 1.00 Hz
GB 0
PC 1.40

^{13}C NMR spectrum of 4-(4-((benzo[d]oxazol-2-ylthio)methyl)-1H-1,2,3-triazol-1-yl)-N-(2-fluorophenyl)-3-oxobutanamide **3**c **(BOK-3)**.

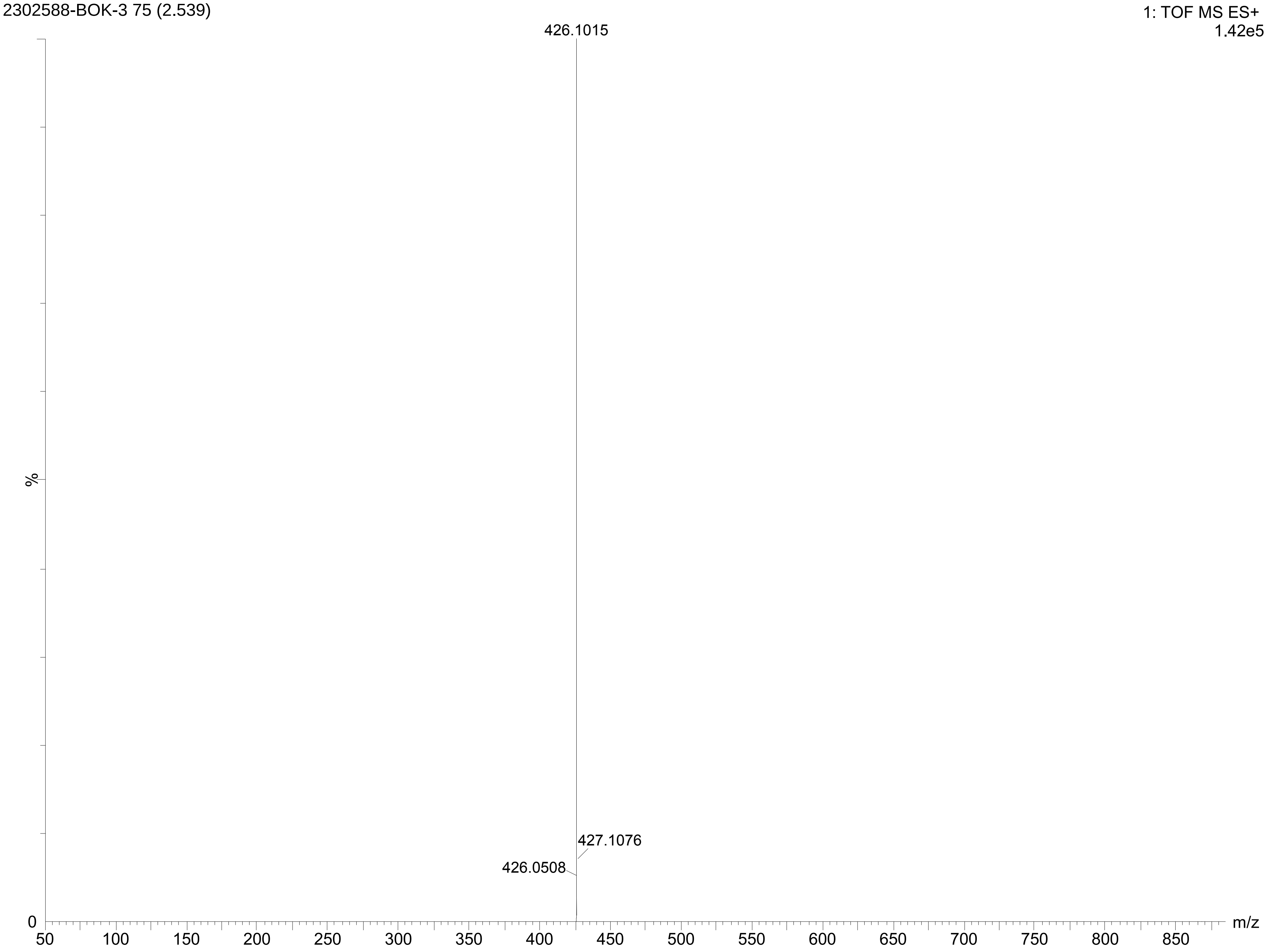

Mass spectrum of 4-(4-((benzo[d]oxazol-2-ylthio)methyl)-1H-1,2,3-triazol-1-yl)-N-(2-fluorophenyl)-3-oxobutanamide **3c (BOK-3)**.

COMPOUND 3d (BOK-4) SPECTRAL DATA

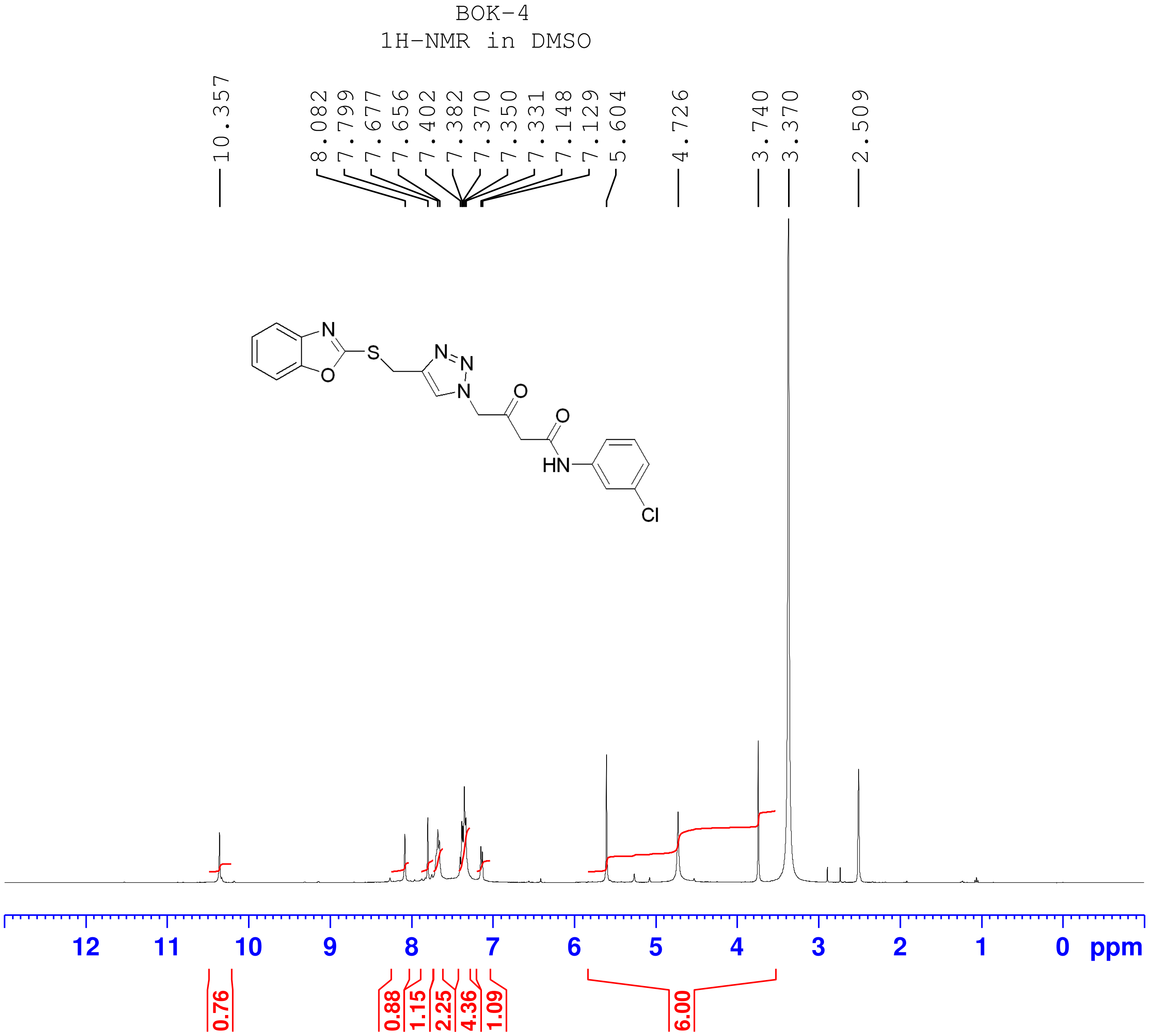

Current Data Parameters

NAME	23000836-BO-3Cl
EXPNO	1
PROCNO	1

F2 - Acquisition Parameters

Date_	20230814
Time	17.56 h
INSTRUM	spect
PROBHD	Z108618_0984 (
PULPROG	zg30
TD	65536
SOLVENT	DMSO
NS	16
DS	2
SWH	8012.820 Hz
FIDRES	0.244532 Hz
AQ	4.0894465 sec
RG	160.67
DW	62.400 usec
DE	17.09 usec
TE	297.8 K
D1	1.00000000 sec
TD0	1
SFO1	400.3124719 MHz
NUC1	1H
P0	4.67 usec
P1	14.00 usec
PLW1	11.28999996 W

F2 - Processing parameters

SI	65536
SF	400.3100000 MHz
WDW	EM
SSB	0
LB	0.30 Hz
GB	0
PC	1.00

^{1}H NMR spectrum of 4-(4-((benzo[d]oxazol-2-ylthio)methyl)-1H-1,2,3-triazol-1-yl)-N-(3-chlorophenyl)-3-oxobutanamide **3d (BOK-4)**

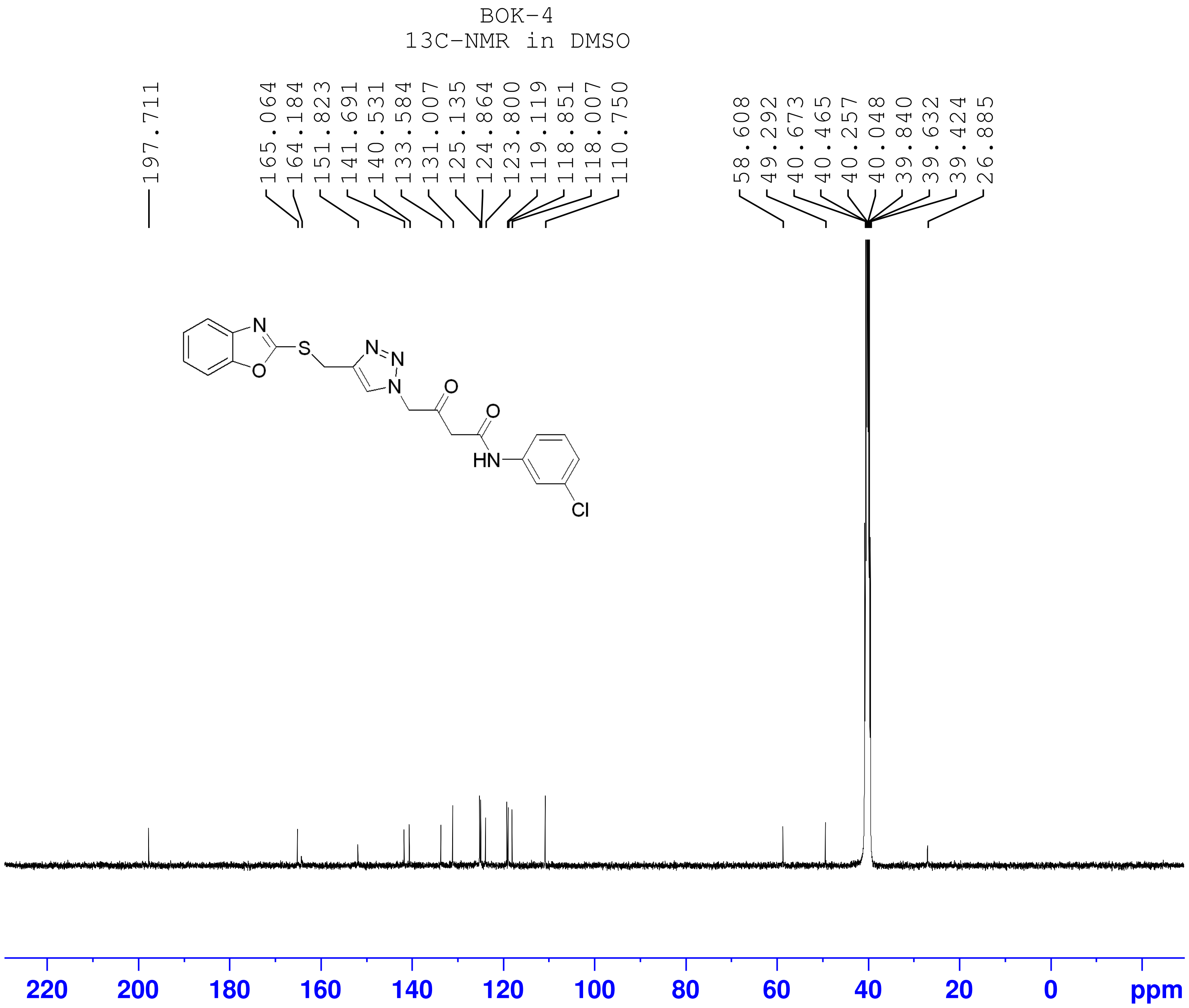

Current Data Parameters	
NAME	23000836-BO-3Cl
EXPNO	2
PROCNO	1

F2 - Acquisition Parameters	
Date_	20230816
Time	11.22 h
INSTRUM	spect
PROBHD	Z108618_0984 (
PULPROG	zgpg30
TD	65536
SOLVENT	DMSO
NS	2048
DS	4
SWH	26041.666 Hz
FIDRES	0.794729 Hz
AQ	1.2582912 sec
RG	202.84
DW	19.200 usec
DE	6.50 usec
TE	298.0 K
D1	1.00000000 sec
D11	0.03000000 sec
TD0	1
SFO1	100.6680954 MHz
NUC1	13C
P0	3.33 usec
P1	10.00 usec
PLW1	51.02600098 W
SFO2	400.3116012 MHz
NUC2	1H
CPDPRG[2	waltz65
PCPD2	90.00 usec
PLW2	11.28999996 W
PLW12	0.27318001 W
PLW13	0.13741000 W

F2 - Processing parameters	
SI	32768
SF	100.6580296 MHz
WDW	EM
SSB	0
LB	1.00 Hz
GB	0
PC	1.40

^{13}C NMR spectrum of 4-(4-((benzo[d]oxazol-2-ylthio)methyl)-1H-1,2,3-triazol-1-yl)-N-(3-chlorophenyl)-3-oxobutanamide **3**d **(BOK**-4**)**.

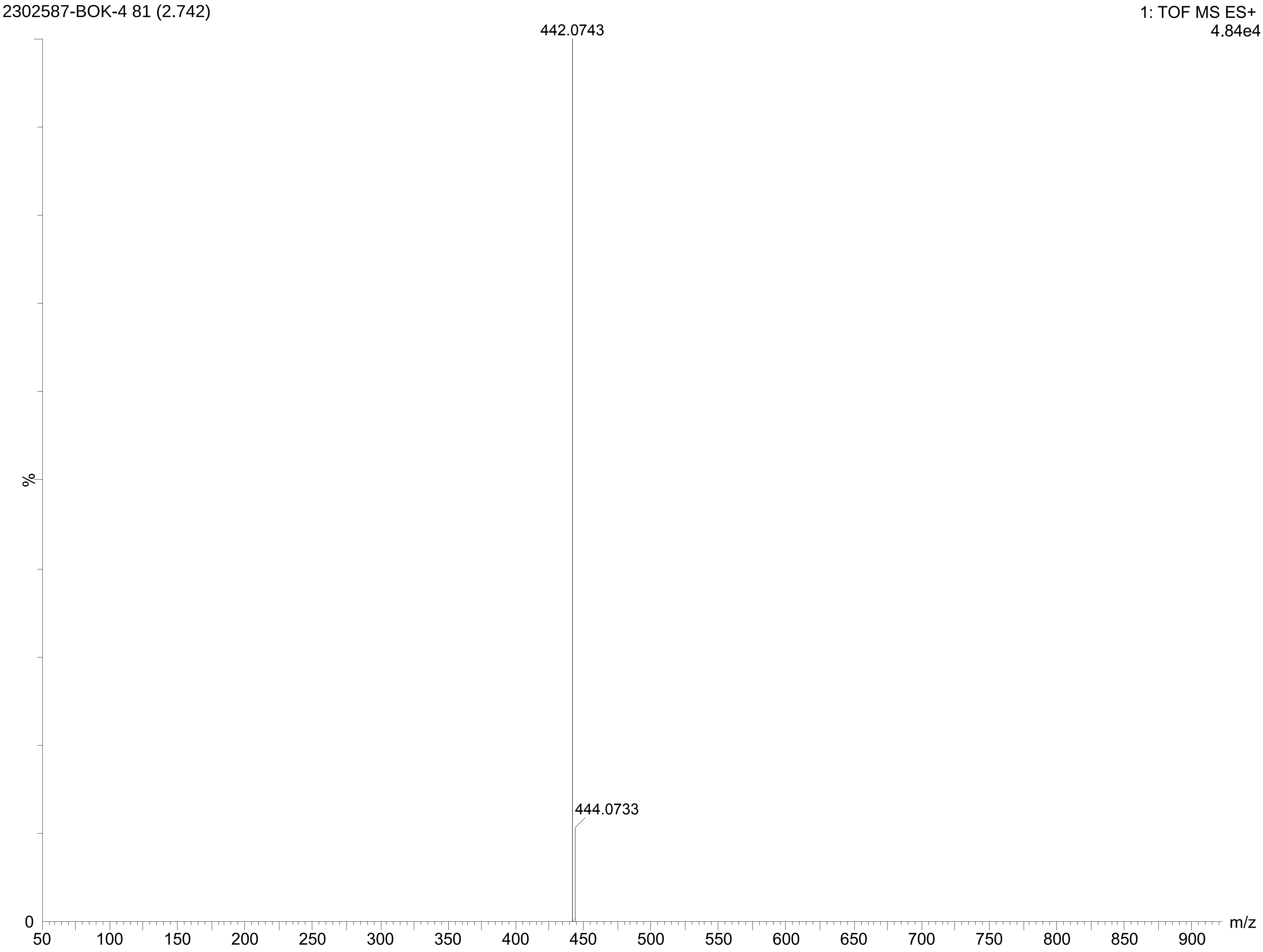

Mass spectrum of 4-(4-((benzo[d]oxazol-2-ylthio)methyl)-1H-1,2,3-triazol-1-yl)-N-(3-chlorophenyl)-3-oxobutanamide **3d (BOK-4)**

COMPOUND 3e (BOK-5) SPECTRAL DATA

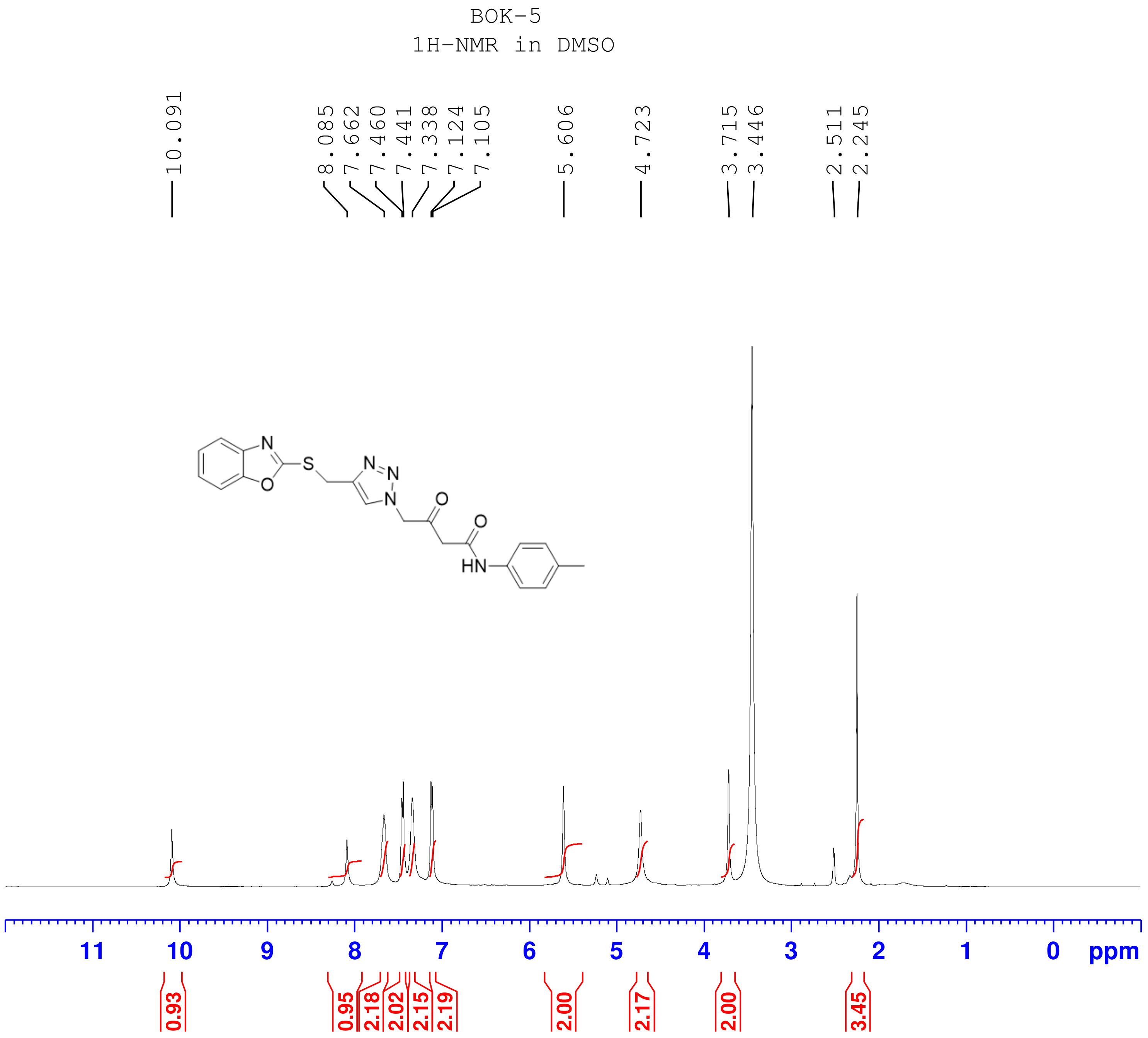

Current Data Parameters
NAME 23000781-BO-4CH3
EXPNO 1
PROCNO 1

F2 - Acquisition Parameters
Date_ 20230801
Time 5.11 h
INSTRUM spect
PROBHD Z108618_0984 (
PULPROG zg30
TD 65536
SOLVENT DMSO
NS 16
DS 2
SWH 8012.820 Hz
FIDRES 0.244532 Hz
AQ 4.0894465 sec
RG 65.15
DW 62.400 usec
DE 17.09 usec
TE 297.0 K
D1 1.00000000 sec
TD0 1
SFO1 400.3124719 MHz
NUC1 1H
P0 4.67 usec
P1 14.00 usec
PLW1 11.28999996 W

F2 - Processing parameters
SI 65536
SF 400.3100000 MHz
WDW EM
SSB 0
LB 0.30 Hz
GB 0
PC 1.00

^{1}H NMR spectrum of 4-(4-((benzo[d]oxazol-2-ylthio)methyl)-1H-1,2,3-triazol-1-yl)-3-oxo-N-(p-tolyl) butanamide **3e (BOK-5).**

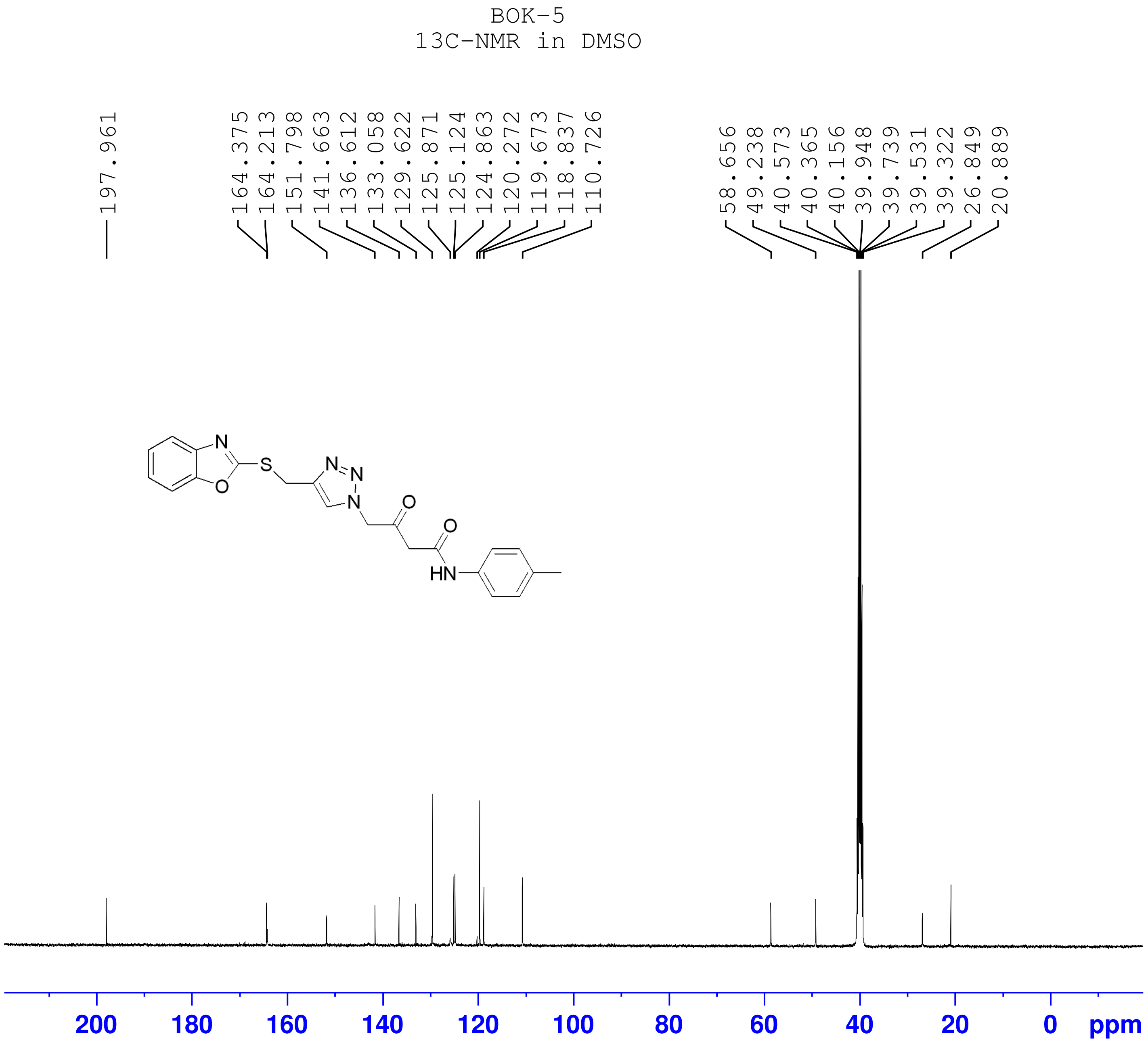

Current Data Parameters
NAME 23000781-BO-4CH3
EXPNO 2
PROCNO 1

F2 - Acquisition Parameters
Date_ 20230801
Time 5.53 h
INSTRUM spect
PROBHD Z108618_0984 (
PULPROG zgpg30
TD 65536
SOLVENT DMSO
NS 1024
DS 4
SWH 24038.461 Hz
FIDRES 0.733596 Hz
AQ 1.3631488 sec
RG 2050
DW 20.800 usec
DE 6.50 usec
TE 297.9 K
D1 1.00000000 sec
D11 0.03000000 sec
TD0 1
SFO1 100.6680954 MHz
NUC1 13C
P0 3.33 usec
P1 10.00 usec
PLW1 51.02600098 W
SFO2 400.3116012 MHz
NUC2 1H
CPDPRG[2 waltz65
PCPD2 90.00 usec
PLW2 11.28999996 W
PLW12 0.27318001 W
PLW13 0.13741000 W

F2 - Processing parameters
SI 32768
SF 100.6580296 MHz
WDW EM
SSB 0
LB 1.00 Hz
GB 0
PC 1.40

^{13}C NMR spectrum of 4-(4-((benzo[d]oxazol-2-ylthio)methyl)-1H-1,2,3-triazol-1-yl)-3-oxo-N-(p-tolyl) butanamide **3e (BOK-5).**

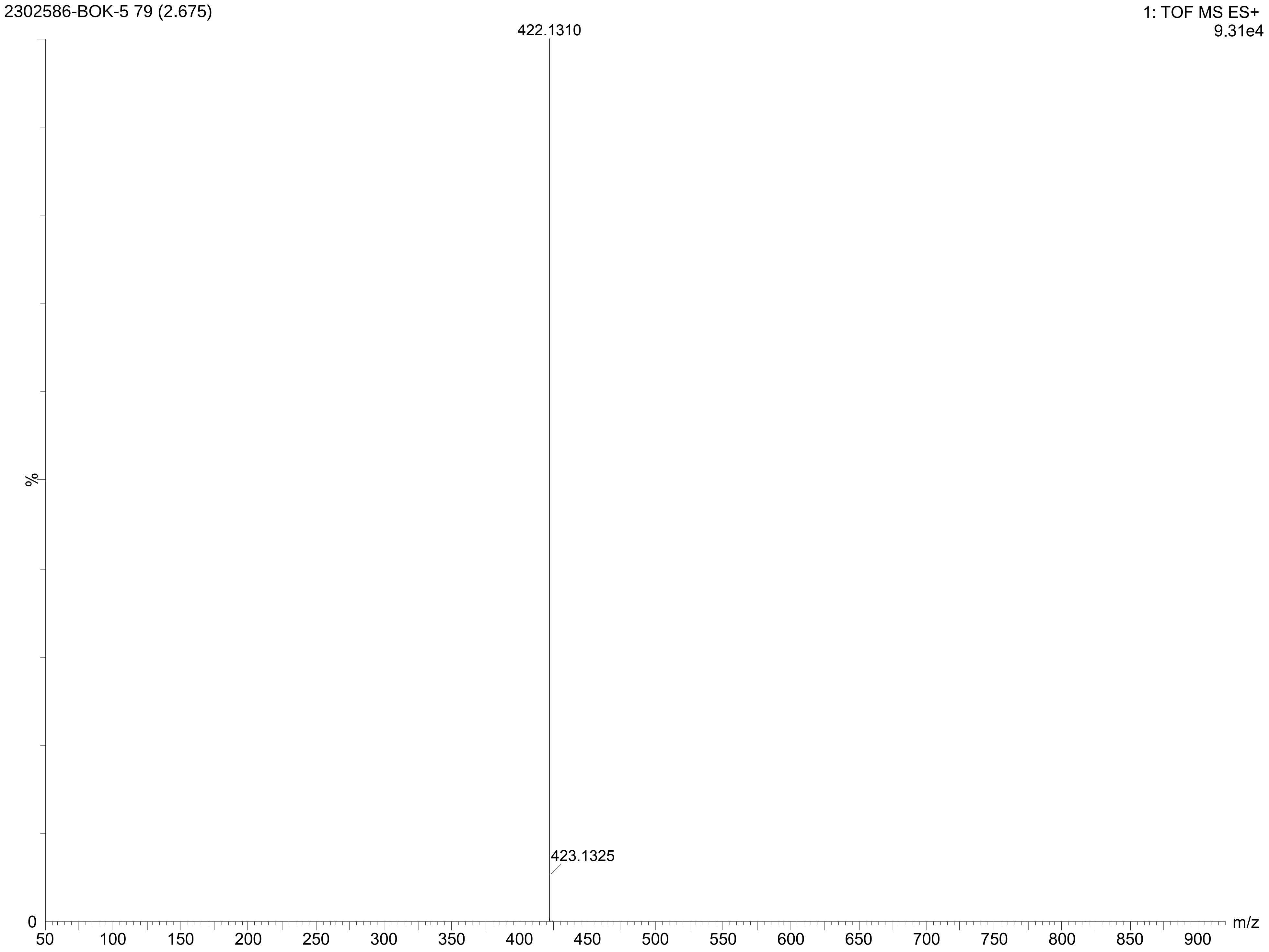

Mass spectrum of 4-(4-((benzo[d]oxazol-2-ylthio)methyl)-1H-1,2,3-triazol-1-yl)-3-oxo-N-(p-tolyl) butanamide**3e (BOK-5).**

COMPOUND 3f (BOK-6) SPECTRAL DATA

BOK-6
1H-NMR in DMSO

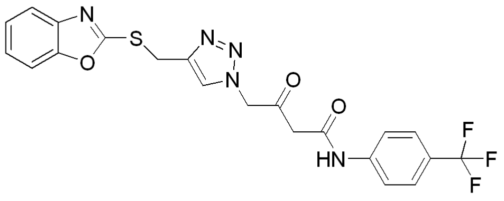

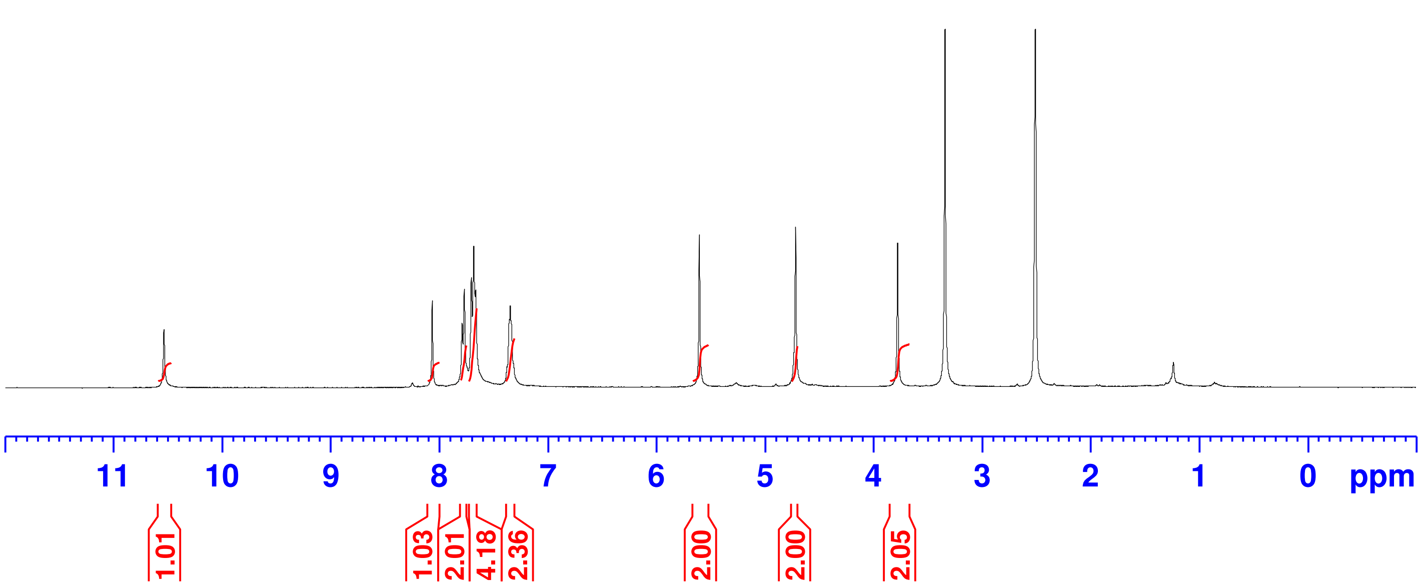

Current Data Parameters
NAME 23000350-BO-CF3
EXPNO 1
PROCNO 1

F2 - Acquisition Parameters
Date_ 20230403
Time 16.06 h
INSTRUM spect
PROBHD Z108618_0984 (
PULPROG zg30
TD 65536
SOLVENT DMSO
NS 16
DS 2
SWH 8012.820 Hz
FIDRES 0.244532 Hz
AQ 4.0894465 sec
RG 255.12
DW 62.400 usec
DE 17.09 usec
TE 297.3 K
D1 1.00000000 sec
TD0 1
SFO1 400.3124719 MHz
NUC1 1H
P0 4.67 usec
P1 14.00 usec
PLW1 11.28999996 W

F2 - Processing parameters
SI 65536
SF 400.3100000 MHz
WDW EM
SSB 0
LB 0.30 Hz
GB 0
PC 1.00

^{1}H NMR spectrum of 4-(4-((benzo[d]oxazol-2-ylthio)methyl)-1H-1,2,3-triazol-1-yl)-3-oxo-N-(4-(trifluoromethyl) phenyl)butanamide **3f (BOK-6).**

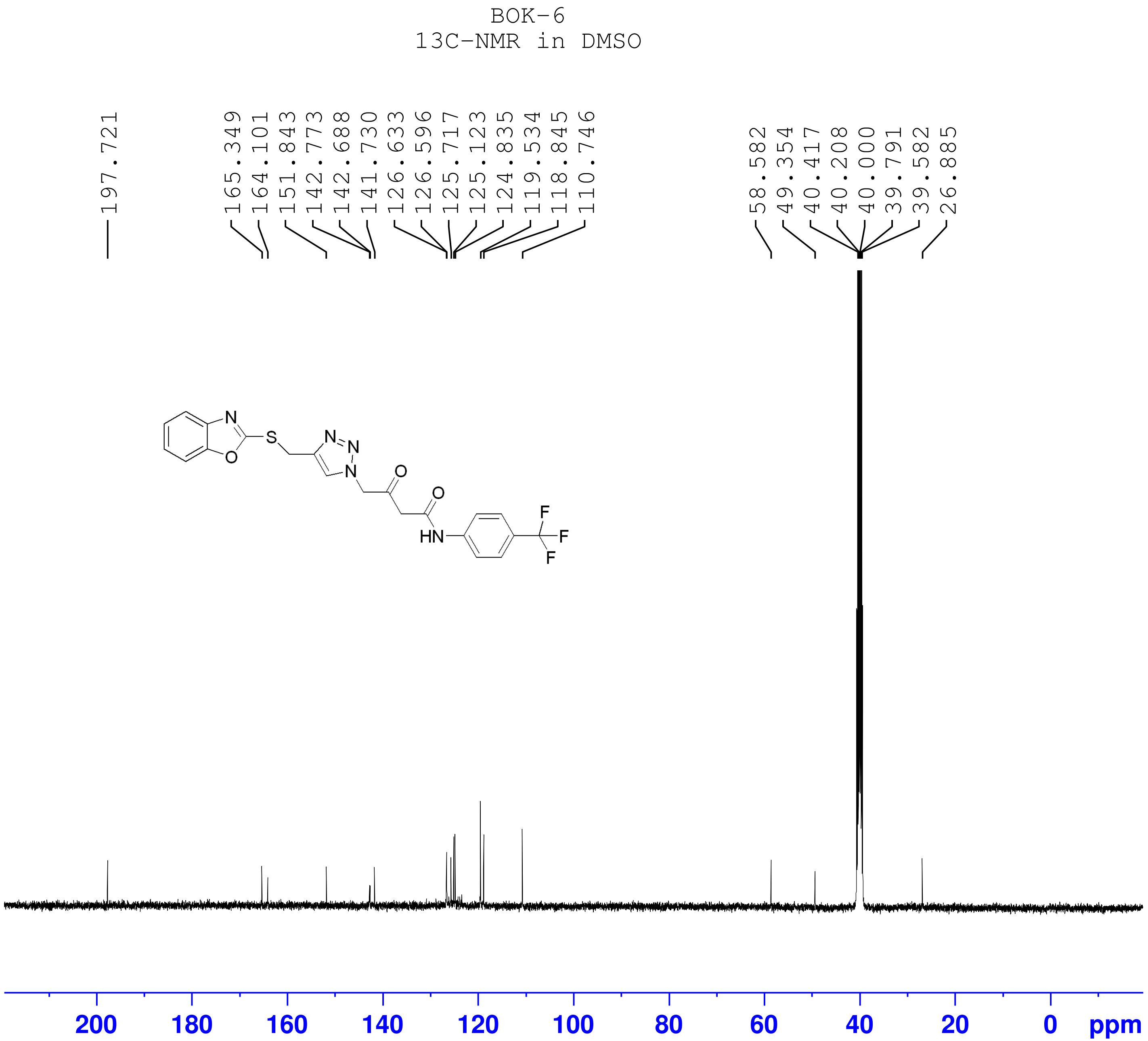

Current Data Parameters
NAME 23000439-BO-4CF3
EXPNO 2
PROCNO 1

F2 - Acquisition Parameters
Date_ 20230430
Time 16.16 h
INSTRUM spect
PROBHD Z108618_0984 (
PULPROG zgpg30
TD 65536
SOLVENT DMSO
NS 1024
DS 4
SWH 24038.461 Hz
FIDRES 0.733596 Hz
AQ 1.3631488 sec
RG 202.84
DW 20.800 usec
DE 6.50 usec
TE 298.5 K
D1 1.00000000 sec
D11 0.03000000 sec
TD0 1
SFO1 100.6680954 MHz
NUC1 13C
P0 3.33 usec
P1 10.00 usec
PLW1 51.02600098 W
SFO2 400.3116012 MHz
NUC2 1H
CPDPRG[2 waltz65
PCPD2 90.00 usec
PLW2 11.28999996 W
PLW12 0.27318001 W
PLW13 0.13741000 W

F2 - Processing parameters
SI 32768
SF 100.6580296 MHz
WDW EM
SSB 0
LB 1.00 Hz
GB 0
PC 1.40

^{13}C NMR spectrum of 4-(4-((benzo[d]oxazol-2-ylthio)methyl)-1H-1,2,3-triazol-1-yl)-3-oxo-N-(4-(trifluoromethyl) phenyl)butanamide **3f (BOK-6)**.

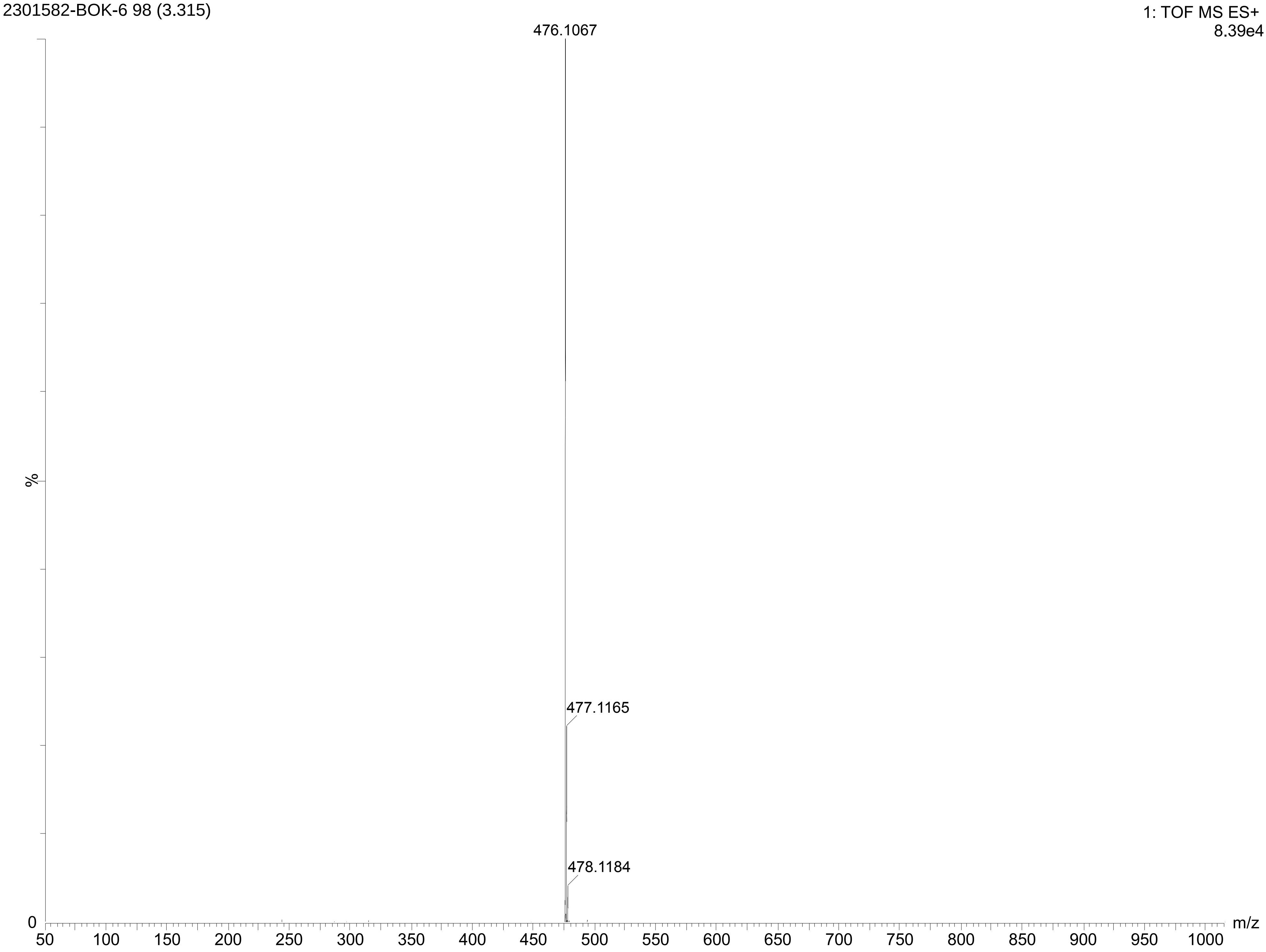

Mass spectrum of 4-(4-((benzo[d]oxazol-2-ylthio)methyl)-1H-1,2,3-triazol-1-yl)-3-oxo-N-(4-(trifluoromethyl) phenyl)butanamide **3f (BOK-6)**.

COMPOUND 3g (BOK-7) SPECTRAL DATA

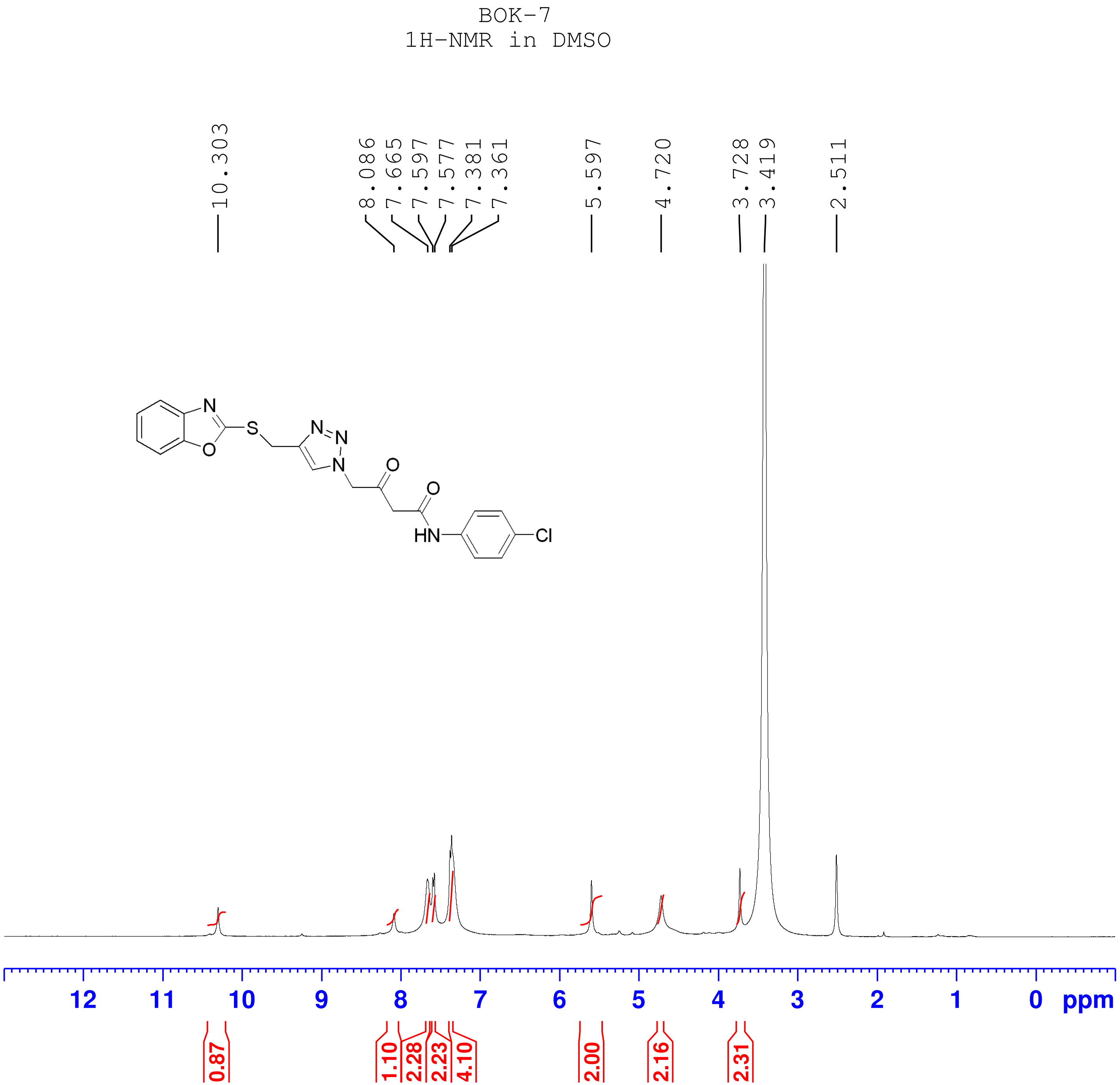


```
Current Data Parameters
NAME        23000783-BO-4CL
EXPNO                     1
PROCNO                    1

F2 - Acquisition Parameters
Date_              20230801
Time                   6.44 h
INSTRUM               spect
PROBHD      Z108618_0984 (
PULPROG                zg30
TD                    65536
SOLVENT                DMSO
NS                       16
DS                        2
SWH                8012.820 Hz
FIDRES             0.244532 Hz
AQ                4.0894465 sec
RG                   114.18
DW                   62.400 usec
DE                    17.09 usec
TE                    297.2 K
D1               1.00000000 sec
TD0                       1
SFO1            400.3124719 MHz
NUC1                     1H
P0                     4.67 usec
P1                    14.00 usec
PLW1            11.28999996 W

F2 - Processing parameters
SI                    65536
SF              400.3100000 MHz
WDW                      EM
SSB                       0
LB                     0.30 Hz
GB                        0
PC                     1.00
```

^{1}H NMR spectrum of 4-(4-((benzo[d]oxazol-2-ylthio)methyl)-1H-1,2,3-triazol-1-yl)-N-(4-chlorophenyl)-3-oxo butanamide **3g (BOK-7).**

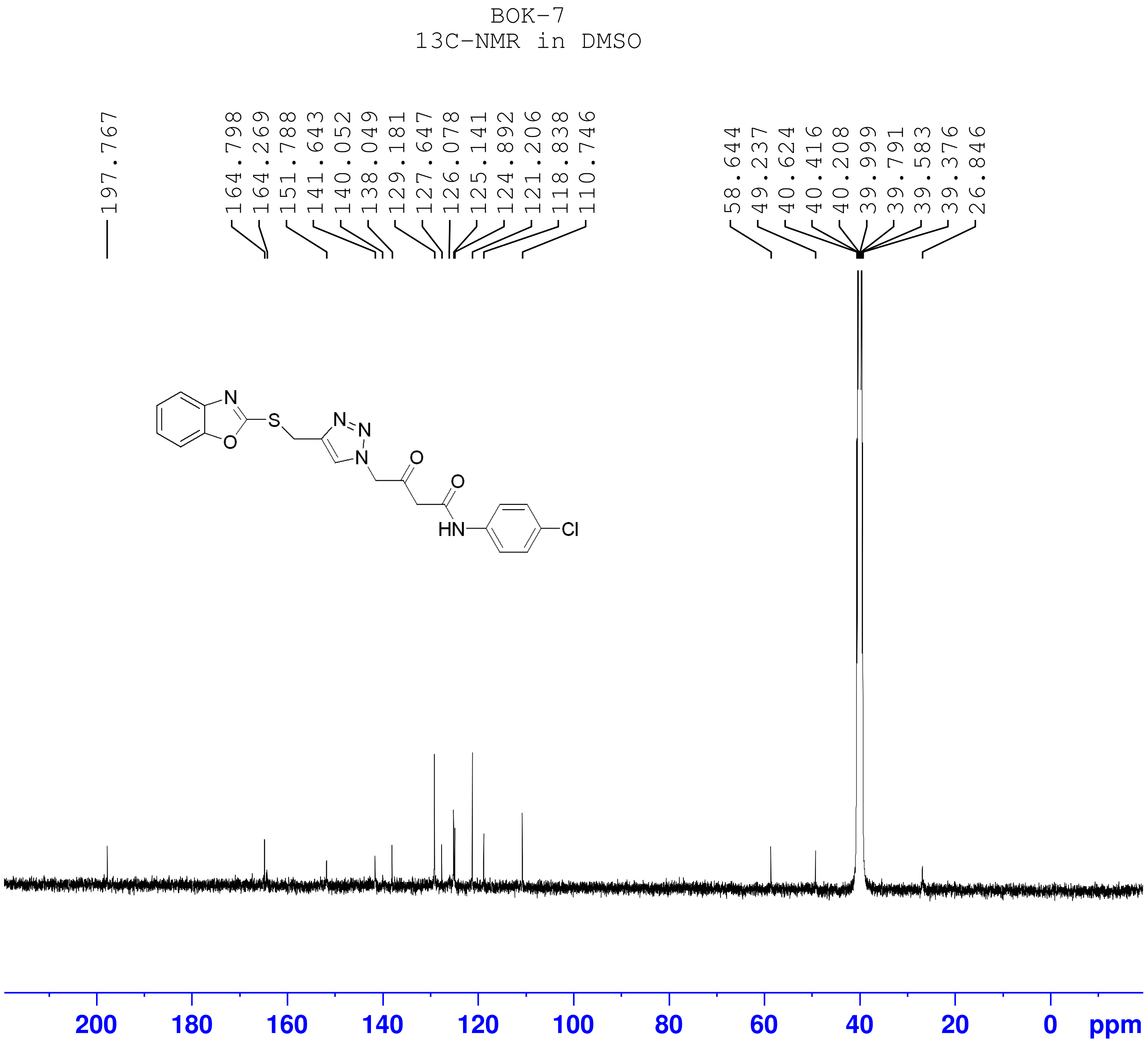


```
Current Data Parameters
NAME       23000783-BO-4CL
EXPNO                    2
PROCNO                   1

F2 - Acquisition Parameters
Date_             20230801
Time                  7.26 h
INSTRUM              spect
PROBHD     Z108618_0984 (
PULPROG            zgpg30
TD                   65536
SOLVENT               DMSO
NS                    1024
DS                       4
SWH             24038.461 Hz
FIDRES           0.733596 Hz
AQ              1.3631488 sec
RG                    2050
DW                  20.800 usec
DE                    6.50 usec
TE                   298.2 K
D1             1.00000000 sec
D11            0.03000000 sec
TD0                      1
SFO1          100.6680954 MHz
NUC1                   13C
P0                    3.33 usec
P1                   10.00 usec
PLW1           51.02600098 W
SFO2          400.3116012 MHz
NUC2                    1H
CPDPRG[2           waltz65
PCPD2                90.00 usec
PLW2           11.28999996 W
PLW12           0.27318001 W
PLW13           0.13741000 W

F2 - Processing parameters
SI                   32768
SF            100.6580296 MHz
WDW                     EM
SSB                      0
LB                    1.00 Hz
GB                       0
PC                    1.40
```

^{13}C NMR spectrum of4-(4-((benzo[d]oxazol-2-ylthio)methyl)-1H-1,2,3-triazol-1-yl)-N-(4-chlorophenyl)-3-oxo butanamide **3g (BOK-7)**.

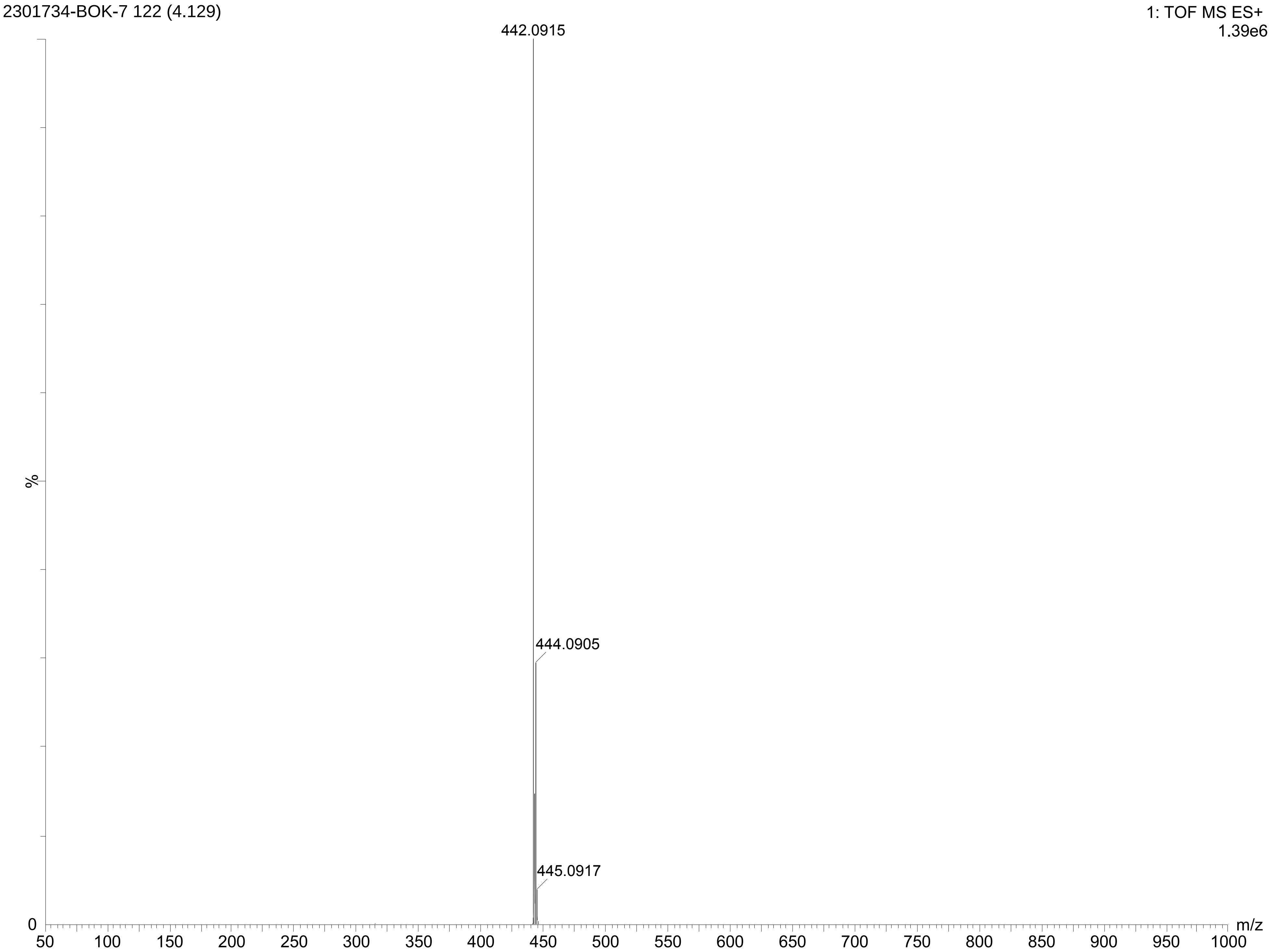

Mass spectrum of 4-(4-((benzo[d]oxazol-2-ylthio)methyl)-1H-1,2,3-triazol-1-yl)-N-(4-chlorophenyl)-3-oxo butanamide **3g (BOK-7)**.

COMPOUND 3h (BOK-8) SPECTRAL DATA

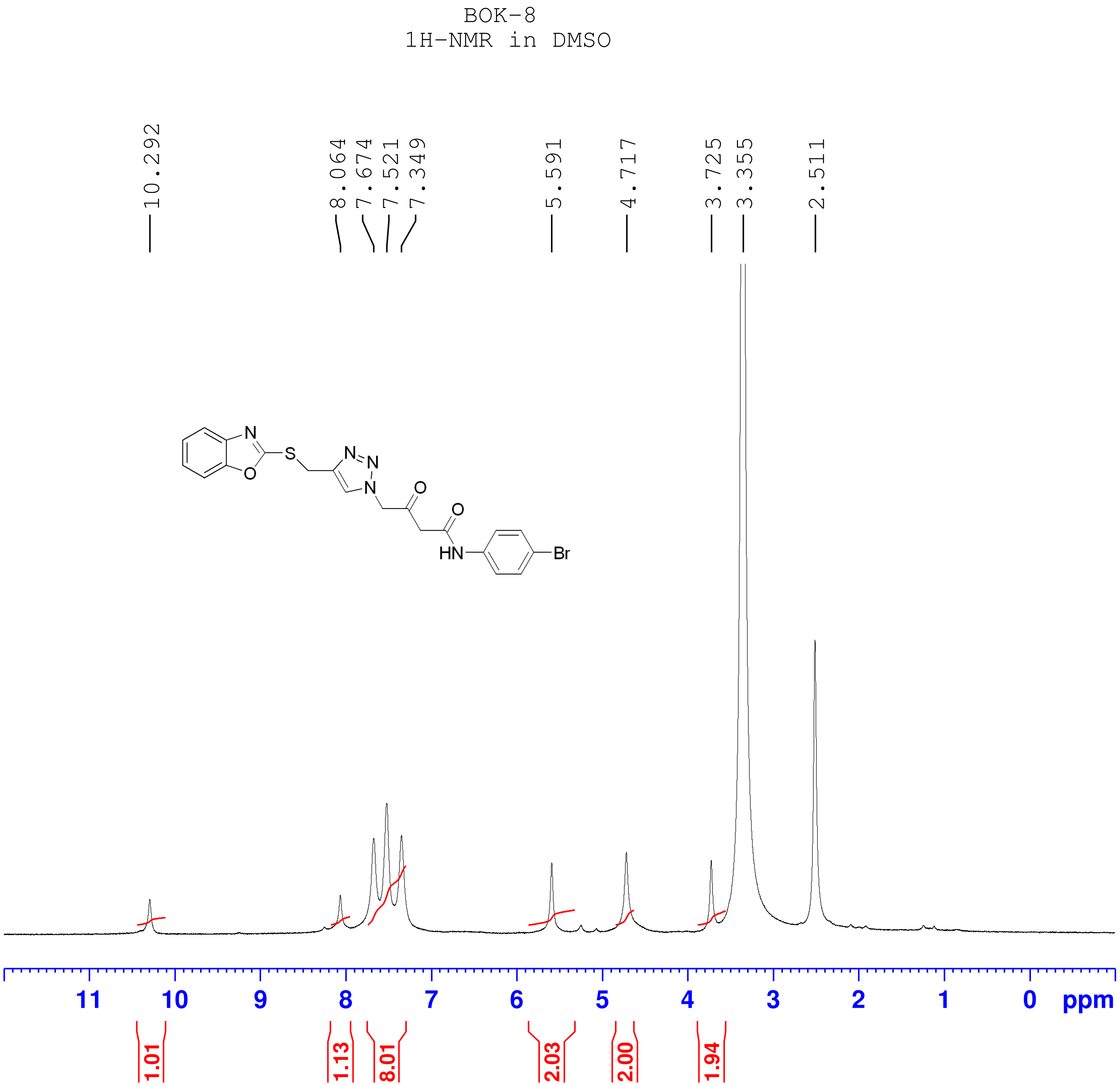

Current Data Parameters
NAME 23000509-BO-4Br
EXPNO 1
PROCNO 1

F2 - Acquisition Parameters
Date_ 20230520
Time 10.36 h
INSTRUM spect
PROBHD Z108618_0984 (
PULPROG zg30
TD 65536
SOLVENT DMSO
NS 32
DS 2
SWH 8012.820 Hz
FIDRES 0.244532 Hz
AQ 4.0894465 sec
RG 32.13
DW 62.400 usec
DE 17.09 usec
TE 297.8 K
D1 1.00000000 sec
TD0 1
SFO1 400.3124719 MHz
NUC1 1H
P0 4.67 usec
P1 14.00 usec
PLW1 11.28999996 W

F2 - Processing parameters
SI 65536
SF 400.3100000 MHz
WDW EM
SSB 0
LB 0.30 Hz
GB 0
PC 1.00

^{1}H NMR spectrum of 4-(4-((benzo[d]oxazol-2-ylthio)methyl)-1H-1,2,3-triazol-1-yl)-N-(4-bromophenyl)-3-oxo butanamide **3h (BOK-8)**.

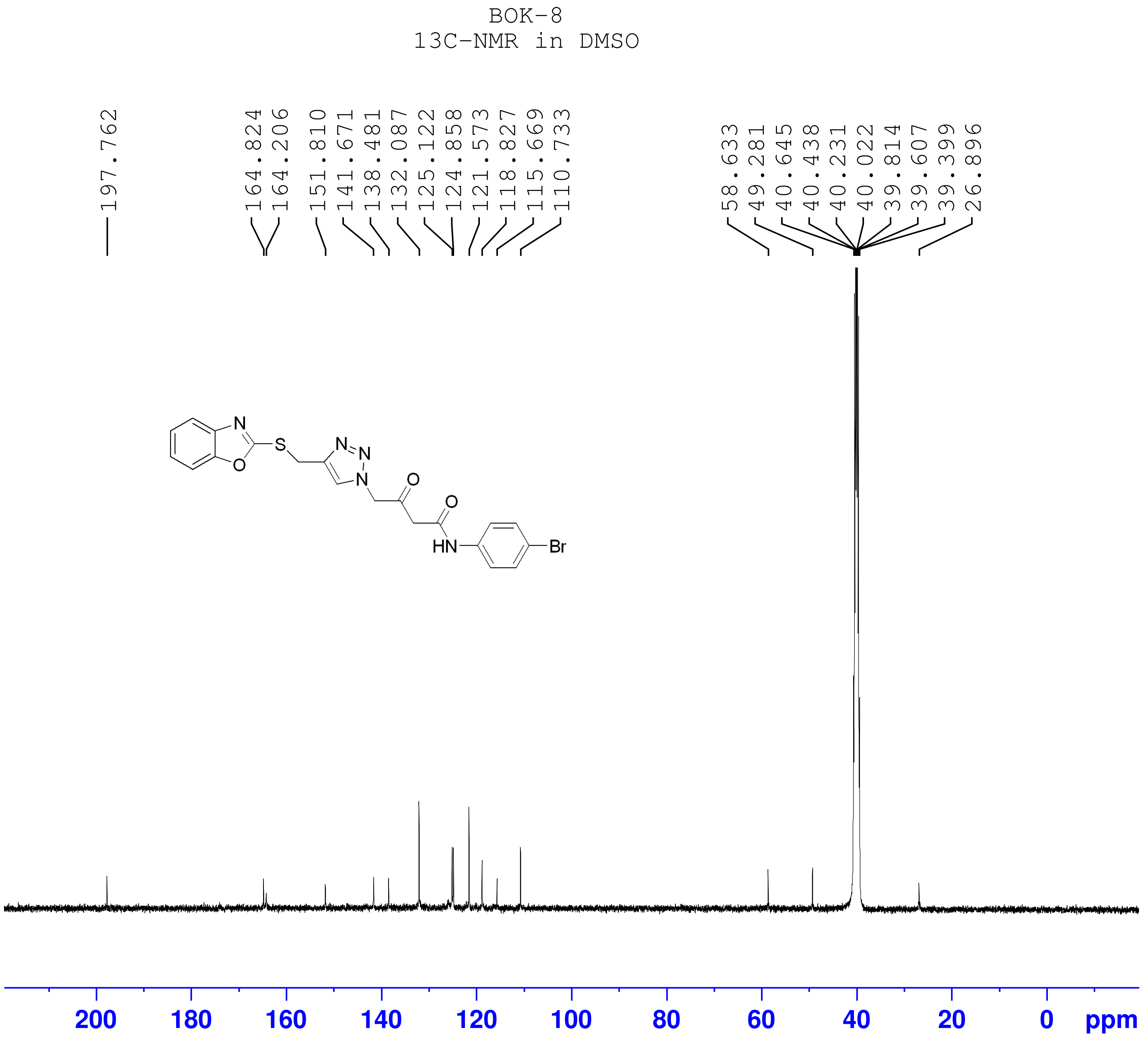

^{13}C NMR spectrum of 4-(4-((benzo[d]oxazol-2-ylthio)methyl)-1H-1,2,3-triazol-1-yl)-N-(4-bromophenyl)-3-oxo butanamide **3h (BOK-8)**.

Current Data Parameters

NAME	23000509-BO-4Br
EXPNO	2
PROCNO	1

F2 - Acquisition Parameters

Date_	20230522
Time	14.40 h
INSTRUM	spect
PROBHD	Z108618_0984 (
PULPROG	zgpg30
TD	65536
SOLVENT	DMSO
NS	1024
DS	4
SWH	26041.666 Hz
FIDRES	0.794729 Hz
AQ	1.2582912 sec
RG	202.84
DW	19.200 usec
DE	6.50 usec
TE	298.8 K
D1	1.00000000 sec
D11	0.03000000 sec
TD0	1
SFO1	100.6680954 MHz
NUC1	13C
P0	3.33 usec
P1	10.00 usec
PLW1	51.02600098 W
SFO2	400.3116012 MHz
NUC2	1H
CPDPRG[2	waltz65
PCPD2	90.00 usec
PLW2	11.28999996 W
PLW12	0.27318001 W
PLW13	0.13741000 W

F2 - Processing parameters

SI	32768
SF	100.6580296 MHz
WDW	EM
SSB	0
LB	1.00 Hz
GB	0
PC	1.40

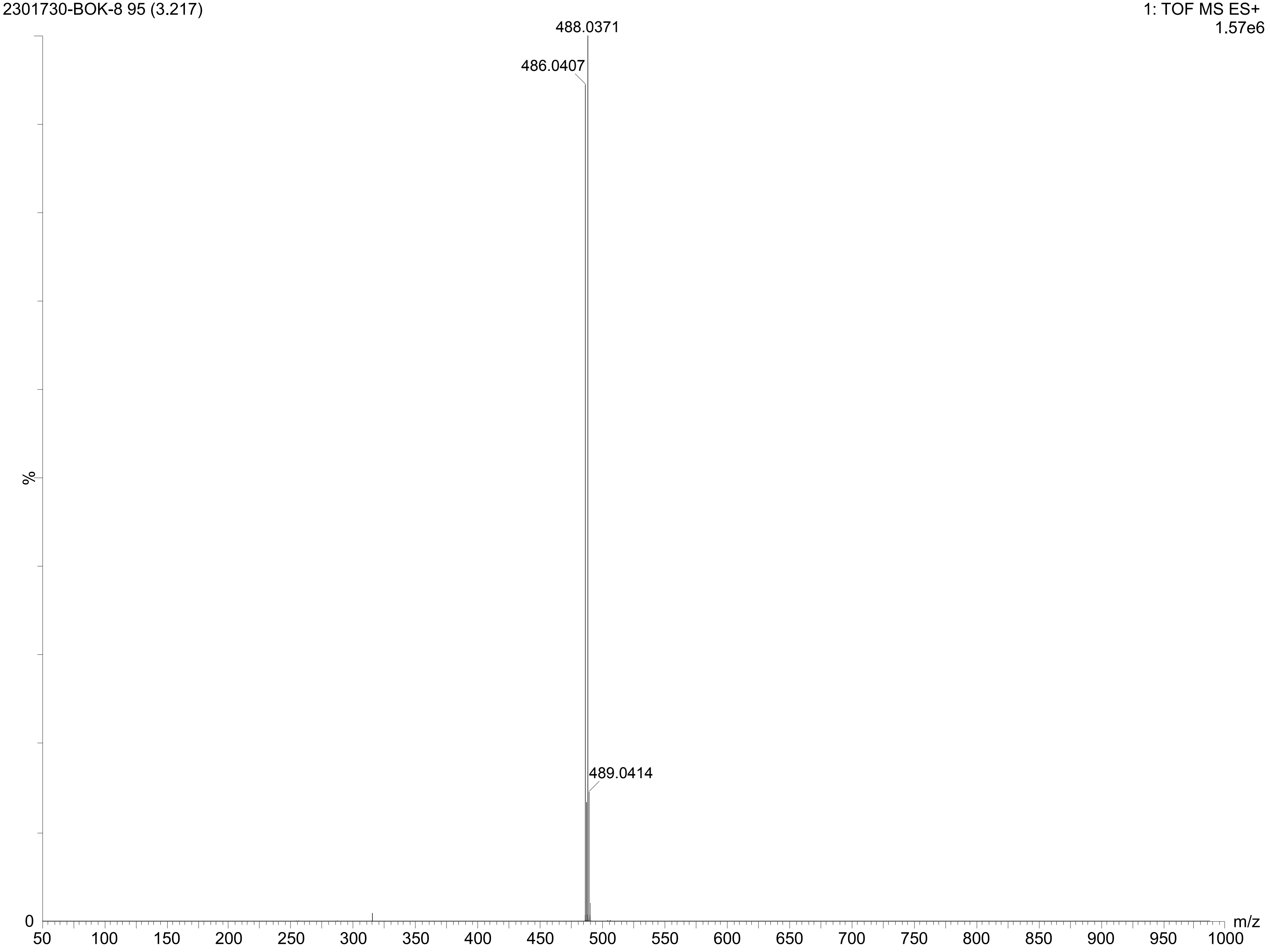

Mass spectrum of 4-(4-((benzo[d]oxazol-2-ylthio)methyl)-1H-1,2,3-triazol-1-yl)-N-(4-bromophenyl)-3-oxo butanamide **3h (BOK-8)**.

COMPOUND 3i (BOK-9) SPECTRAL DATA

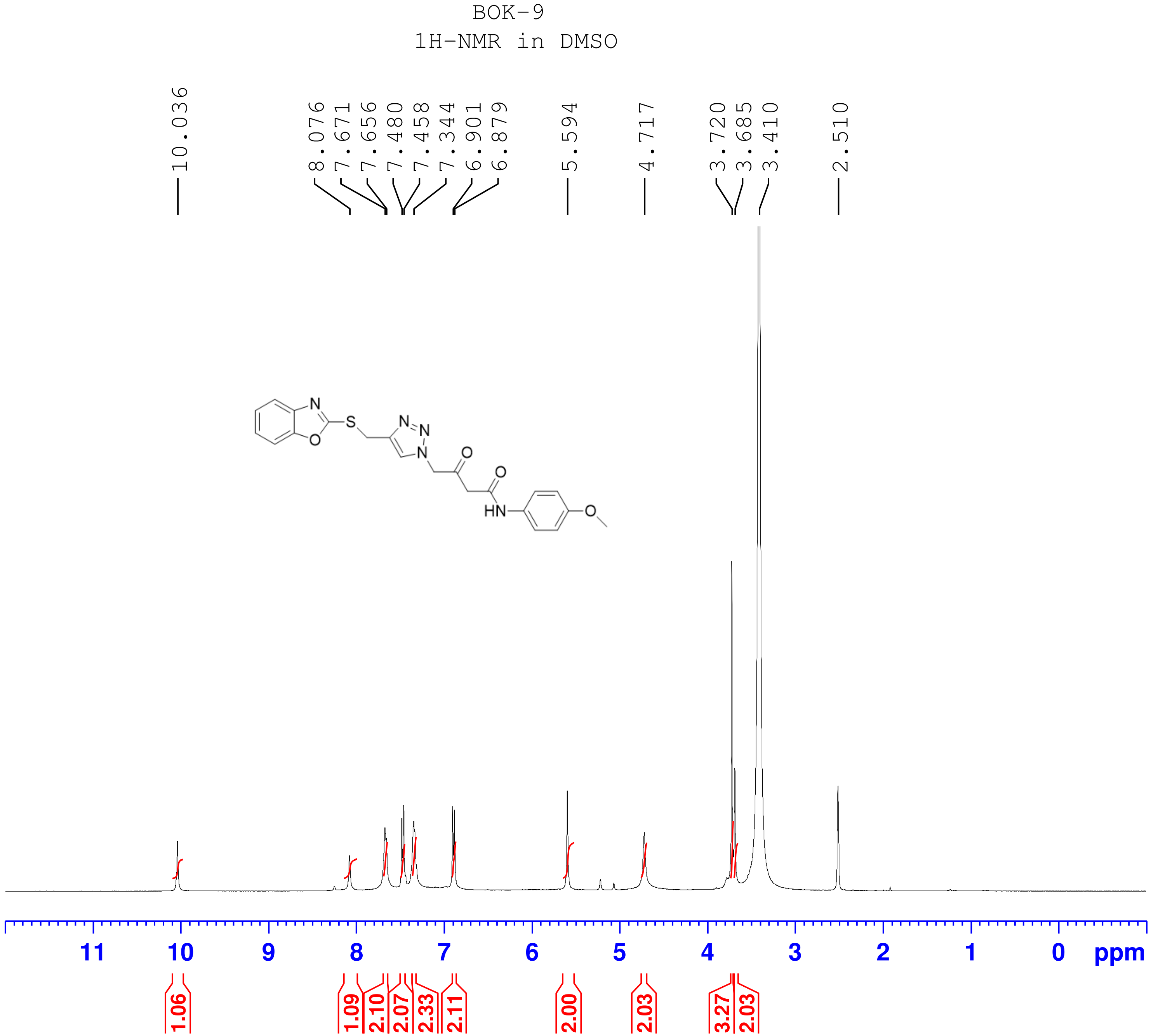

Current Data Parameters
NAME 23000782-BO-4OCH3
EXPNO 1
PROCNO 1

F2 - Acquisition Parameters
Date_ 20230801
Time 5.57 h
INSTRUM spect
PROBHD Z108618_0984 (
PULPROG zg30
TD 65536
SOLVENT DMSO
NS 16
DS 2
SWH 8012.820 Hz
FIDRES 0.244532 Hz
AQ 4.0894465 sec
RG 114.18
DW 62.400 usec
DE 17.09 usec
TE 296.9 K
D1 1.00000000 sec
TD0 1
SFO1 400.3124719 MHz
NUC1 1H
P0 4.67 usec
P1 14.00 usec
PLW1 11.28999996 W

F2 - Processing parameters
SI 65536
SF 400.3100000 MHz
WDW EM
SSB 0
LB 0.30 Hz
GB 0
PC 1.00

^{1}H NMR spectrum of 4-(4-((benzo[d]oxazol-2-ylthio)methyl)-1H-1,2,3-triazol-1-yl)-N-(4-methoxyphenyl)-3-oxo butanamide **3i (BOK-9)**.

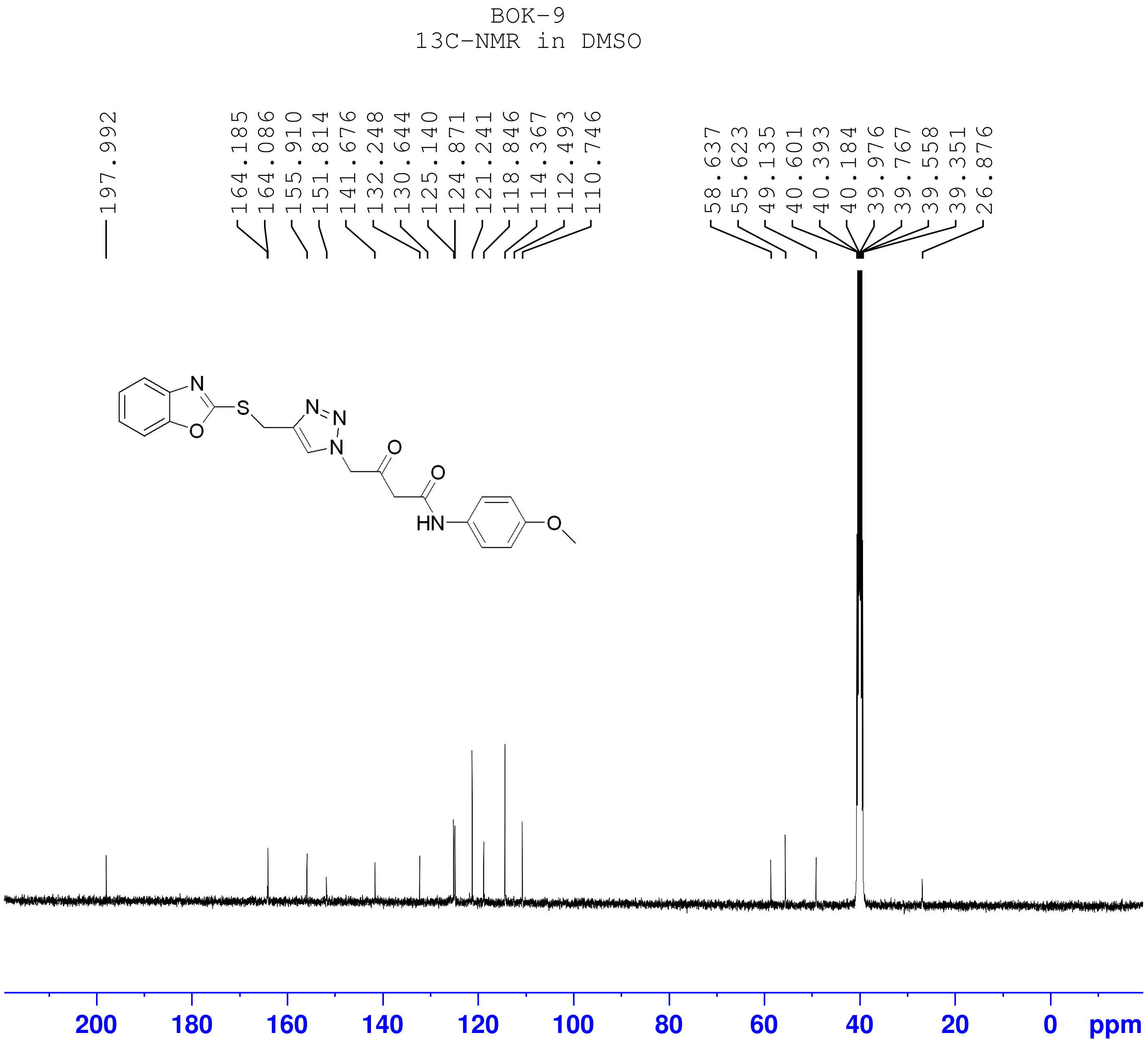

Current Data Parameters
NAME 23000782-BO-4OCH3
EXPNO 2
PROCNO 1

F2 - Acquisition Parameters
Date_ 20230801
Time 6.40 h
INSTRUM spect
PROBHD Z108618_0984 (
PULPROG zgpg30
TD 65536
SOLVENT DMSO
NS 1024
DS 4
SWH 24038.461 Hz
FIDRES 0.733596 Hz
AQ 1.3631488 sec
RG 2050
DW 20.800 usec
DE 6.50 usec
TE 297.9 K
D1 1.00000000 sec
D11 0.03000000 sec
TD0 1
SFO1 100.6680954 MHz
NUC1 13C
P0 3.33 usec
P1 10.00 usec
PLW1 51.02600098 W
SFO2 400.3116012 MHz
NUC2 1H
CPDPRG[2 waltz65
PCPD2 90.00 usec
PLW2 11.28999996 W
PLW12 0.27318001 W
PLW13 0.13741000 W

F2 - Processing parameters
SI 32768
SF 100.6580296 MHz
WDW EM
SSB 0
LB 1.00 Hz
GB 0
PC 1.40

^{13}C NMR spectrum of 4-(4-((benzo[d]oxazol-2-ylthio)methyl)-1H-1,2,3-triazol-1-yl)-N-(4-methoxyphenyl)-3-oxo butanamide **3i (BOK-9)**.

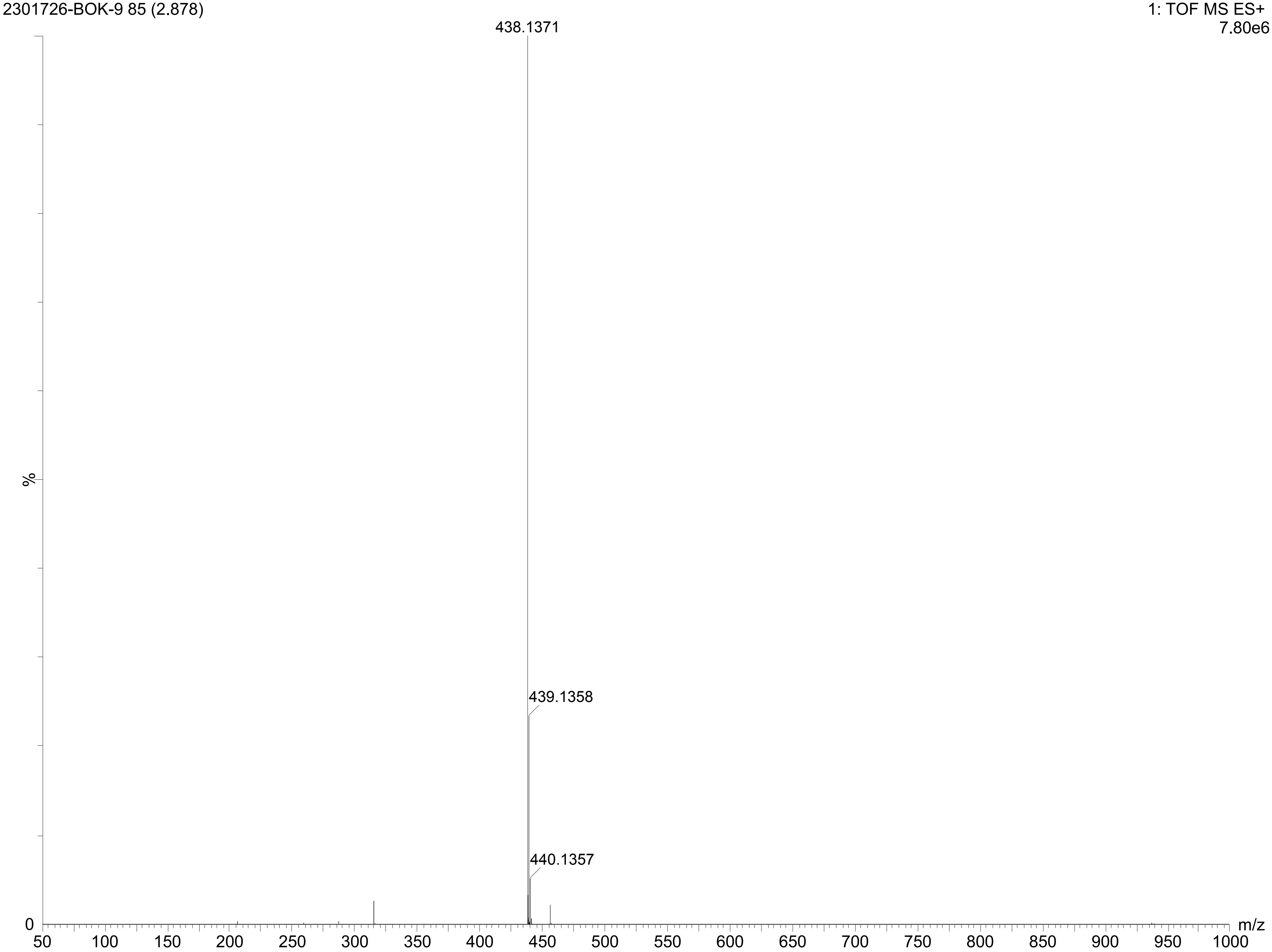

Mass spectrum of 4-(4-((benzo[d]oxazol-2-ylthio)methyl)-1H-1,2,3-triazol-1-yl)-N-(4-methoxyphenyl)-3-oxo butanamide **3i (BOK-9)**.

COMPOUND 3j (BOK-10) SPECTRAL DATA

BOK-10
1H-NMR in DMSO

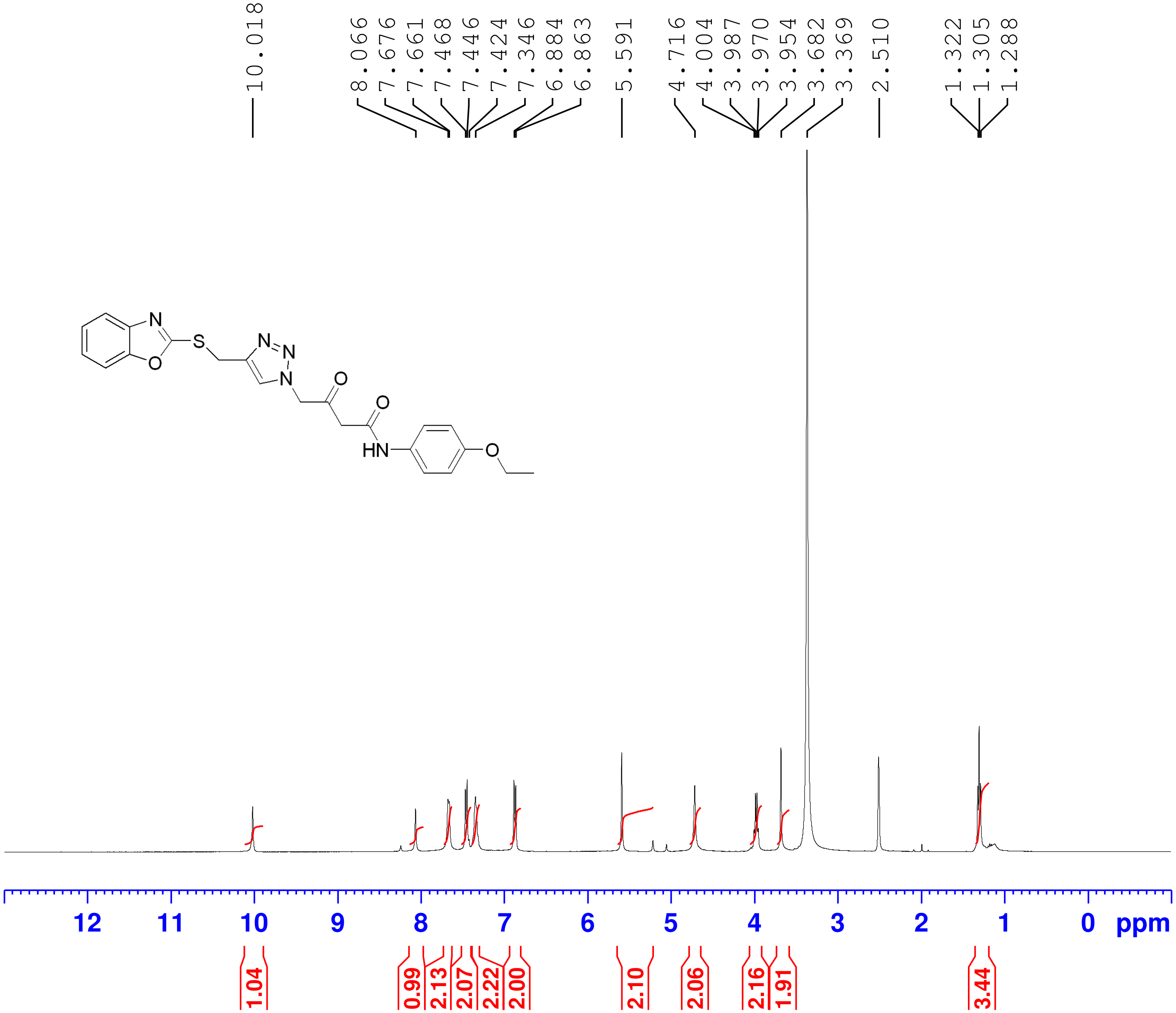

Current Data Parameters
NAME 23000512-Bo-4EO
EXPNO 1
PROCNO 1

F2 - Acquisition Parameters
Date_ 20230520
Time 10.54 h
INSTRUM spect
PROBHD Z108618_0984 (
PULPROG zg30
TD 65536
SOLVENT DMSO
NS 32
DS 2
SWH 8012.820 Hz
FIDRES 0.244532 Hz
AQ 4.0894465 sec
RG 32.13
DW 62.400 usec
DE 17.09 usec
TE 297.5 K
D1 1.00000000 sec
TD0 1
SFO1 400.3124719 MHz
NUC1 1H
P0 4.67 usec
P1 14.00 usec
PLW1 11.28999996 W

F2 - Processing parameters
SI 65536
SF 400.3100000 MHz
WDW EM
SSB 0
LB 0.30 Hz
GB 0
PC 1.00

^{1}H NMR spectrum of 4-(4-((benzo[d]oxazol-2-ylthio)methyl)-1H-1,2,3-triazol-1-yl)-N-(4-ethoxyphenyl)-3-oxo butanamide **3j (BOK-10)**

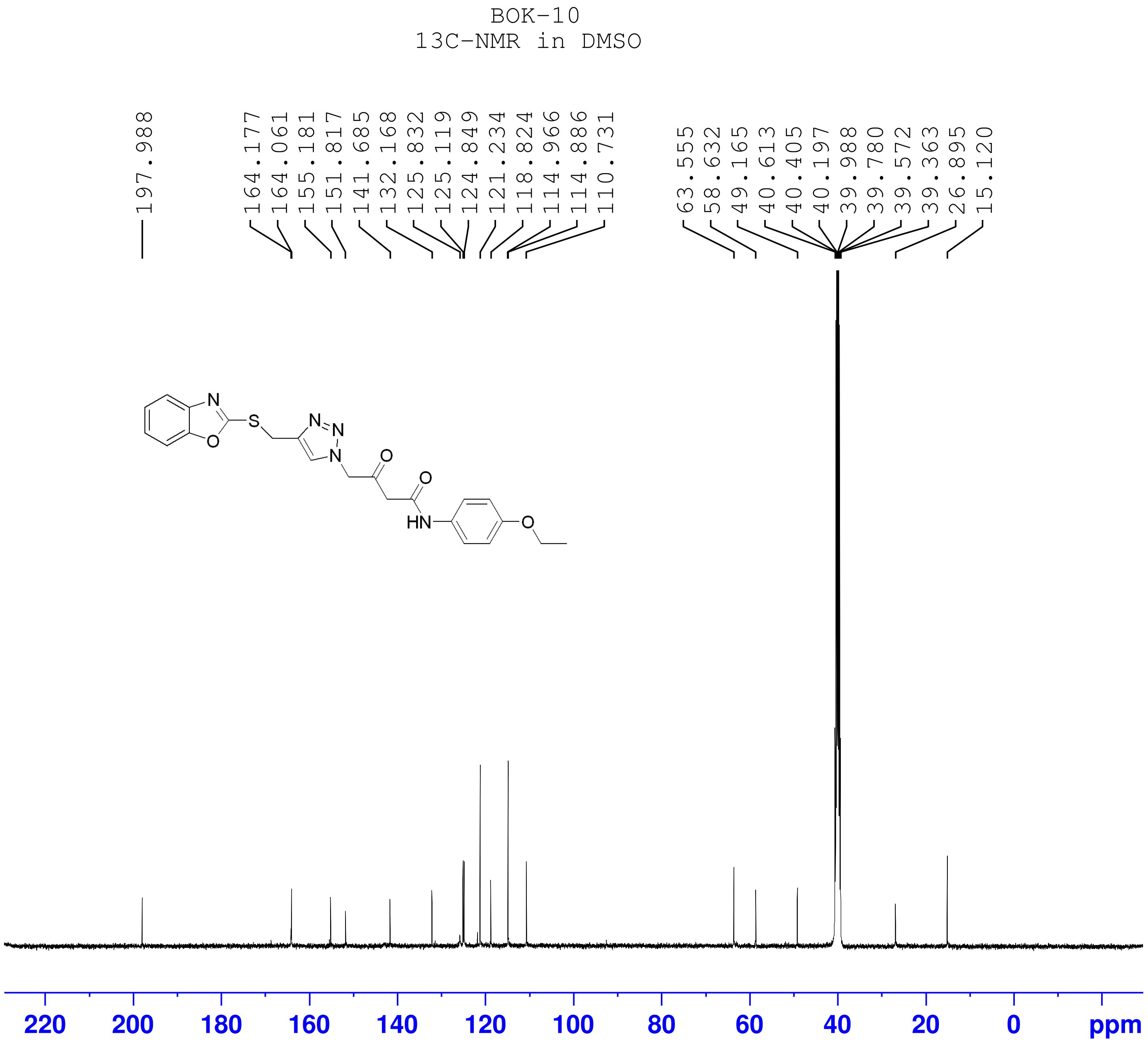

Current Data Parameters
NAME 23000512-Bo-4EO
EXPNO 2
PROCNO 1

F2 - Acquisition Parameters
Date_ 20230522
Time 10.24 h
INSTRUM spect
PROBHD Z108618_0984 (
PULPROG zgpg30
TD 65536
SOLVENT DMSO
NS 1024
DS 4
SWH 26041.666 Hz
FIDRES 0.794729 Hz
AQ 1.2582912 sec
RG 202.84
DW 19.200 usec
DE 6.50 usec
TE 298.8 K
D1 1.00000000 sec
D11 0.03000000 sec
TD0 1
SFO1 100.6680954 MHz
NUC1 13C
P0 3.33 usec
P1 10.00 usec
PLW1 51.02600098 W
SFO2 400.3116012 MHz
NUC2 1H
CPDPRG[2 waltz65
PCPD2 90.00 usec
PLW2 11.28999996 W
PLW12 0.27318001 W
PLW13 0.13741000 W

F2 - Processing parameters
SI 32768
SF 100.6580296 MHz
WDW EM
SSB 0
LB 1.00 Hz
GB 0
PC 1.40

^{13}C NMR spectrum of 4-(4-((benzo[d]oxazol-2-ylthio)methyl)-1H-1,2,3-triazol-1-yl)-N-(4-ethoxyphenyl)-3-oxo butanamide **3j (BOK-10)**.

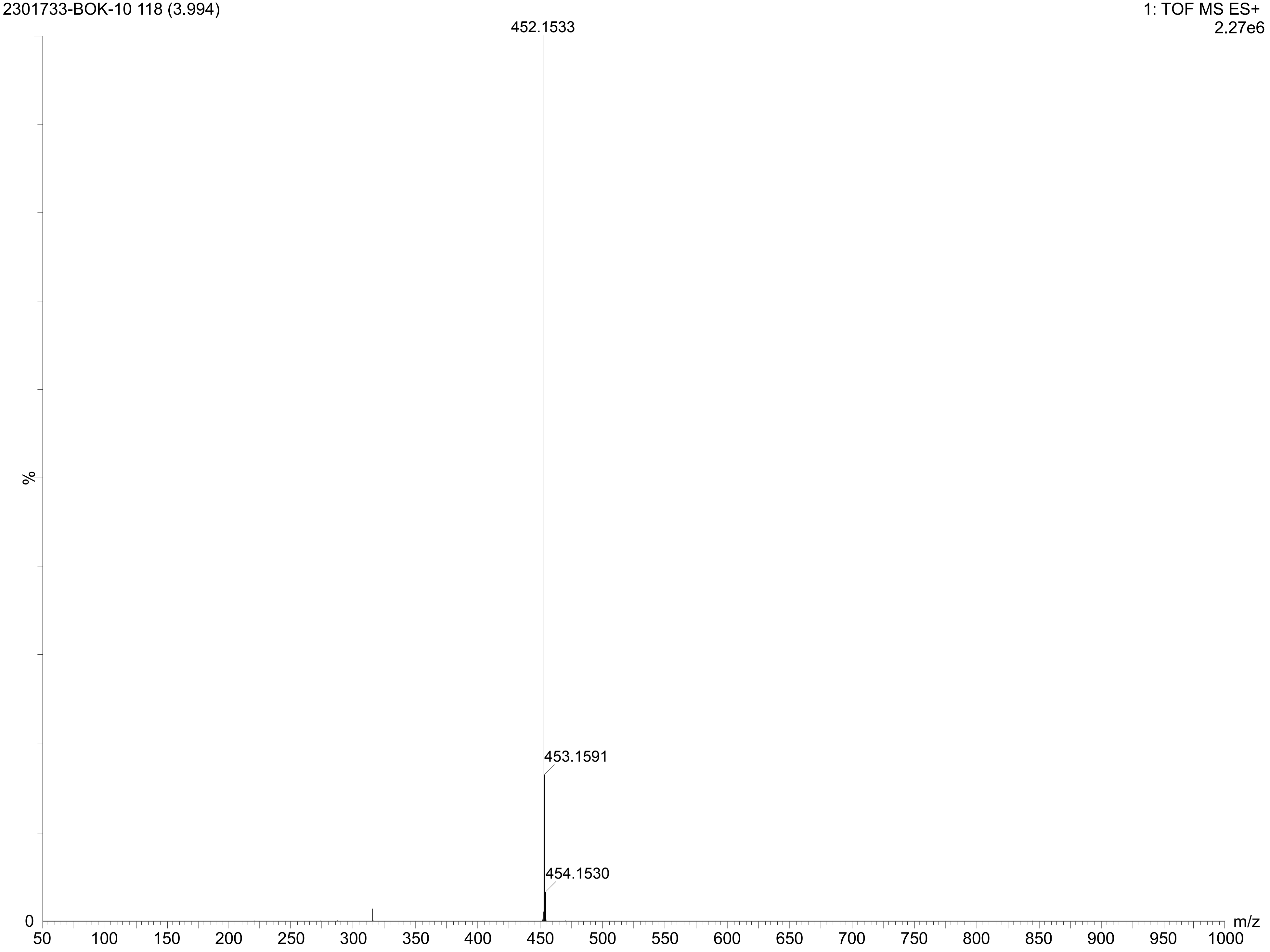

Mass spectrum of 4-(4-((benzo[d]oxazol-2-ylthio)methyl)-1H-1,2,3-triazol-1-yl)-N-(4-ethoxyphenyl)-3-oxo butanamide **3j (BOK-10)**.

COMPOUND 3k (BOP-1) SPECTRAL DATA

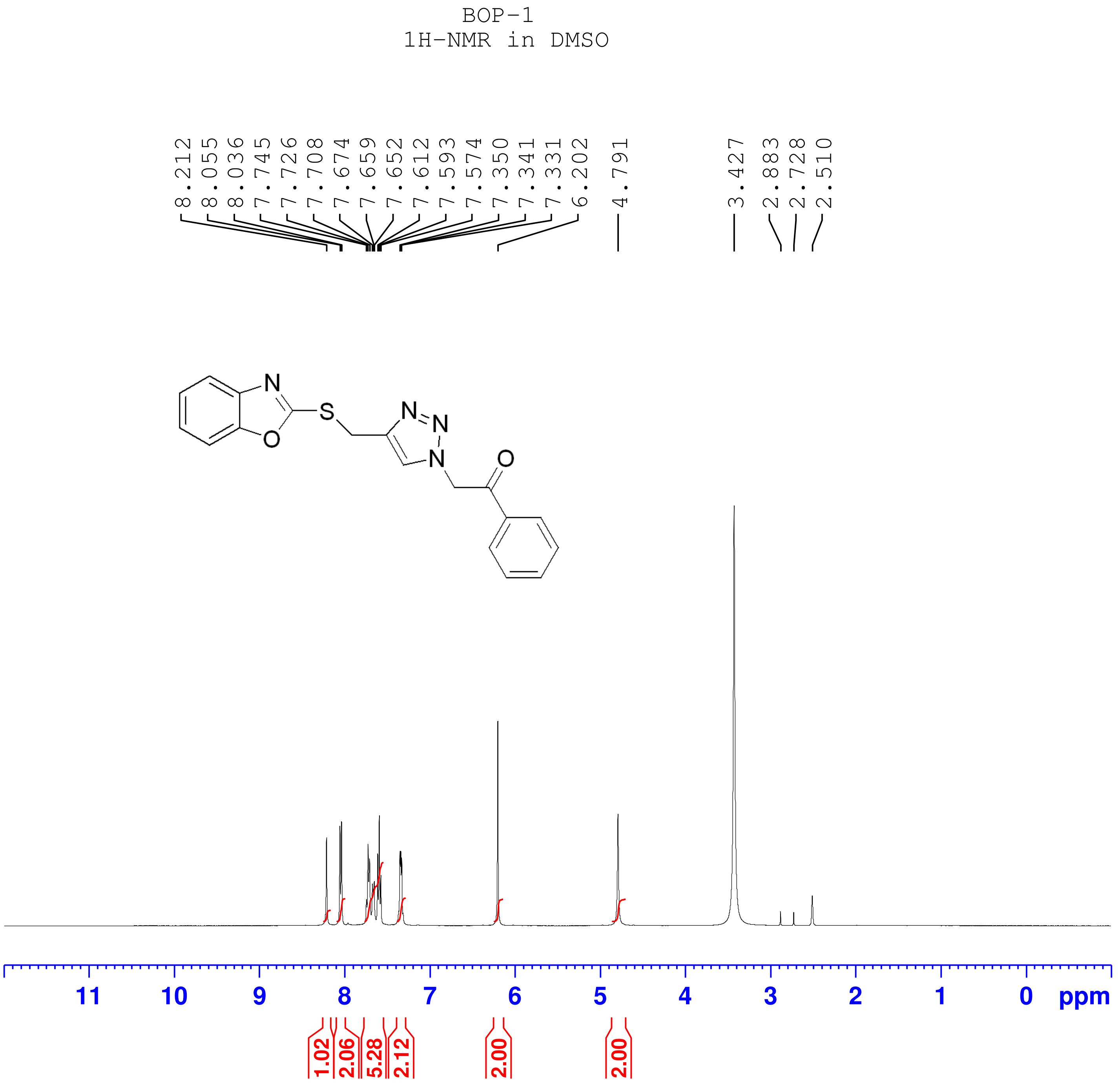

Current Data Parameters
NAME 23000787-Ph-BO
EXPNO 1
PROCNO 1

F2 - Acquisition Parameters
Date_ 20230801
Time 9.19 h
INSTRUM spect
PROBHD Z108618_0984 (
PULPROG zg30
TD 65536
SOLVENT DMSO
NS 16
DS 2
SWH 8012.820 Hz
FIDRES 0.244532 Hz
AQ 4.0894465 sec
RG 80.45
DW 62.400 usec
DE 17.09 usec
TE 297.0 K
D1 1.00000000 sec
TD0 1
SFO1 400.3124719 MHz
NUC1 1H
P0 4.67 usec
P1 14.00 usec
PLW1 11.28999996 W

F2 - Processing parameters
SI 65536
SF 400.3100000 MHz
WDW EM
SSB 0
LB 0.30 Hz
GB 0
PC 1.00

^{1}H NMR spectrum of 2-(4-((benzo[d]oxazol-2-ylthio)methyl)-1H-1,2,3-triazol-1-yl)-1-phenylethan-1-one 3k (BOP-1).

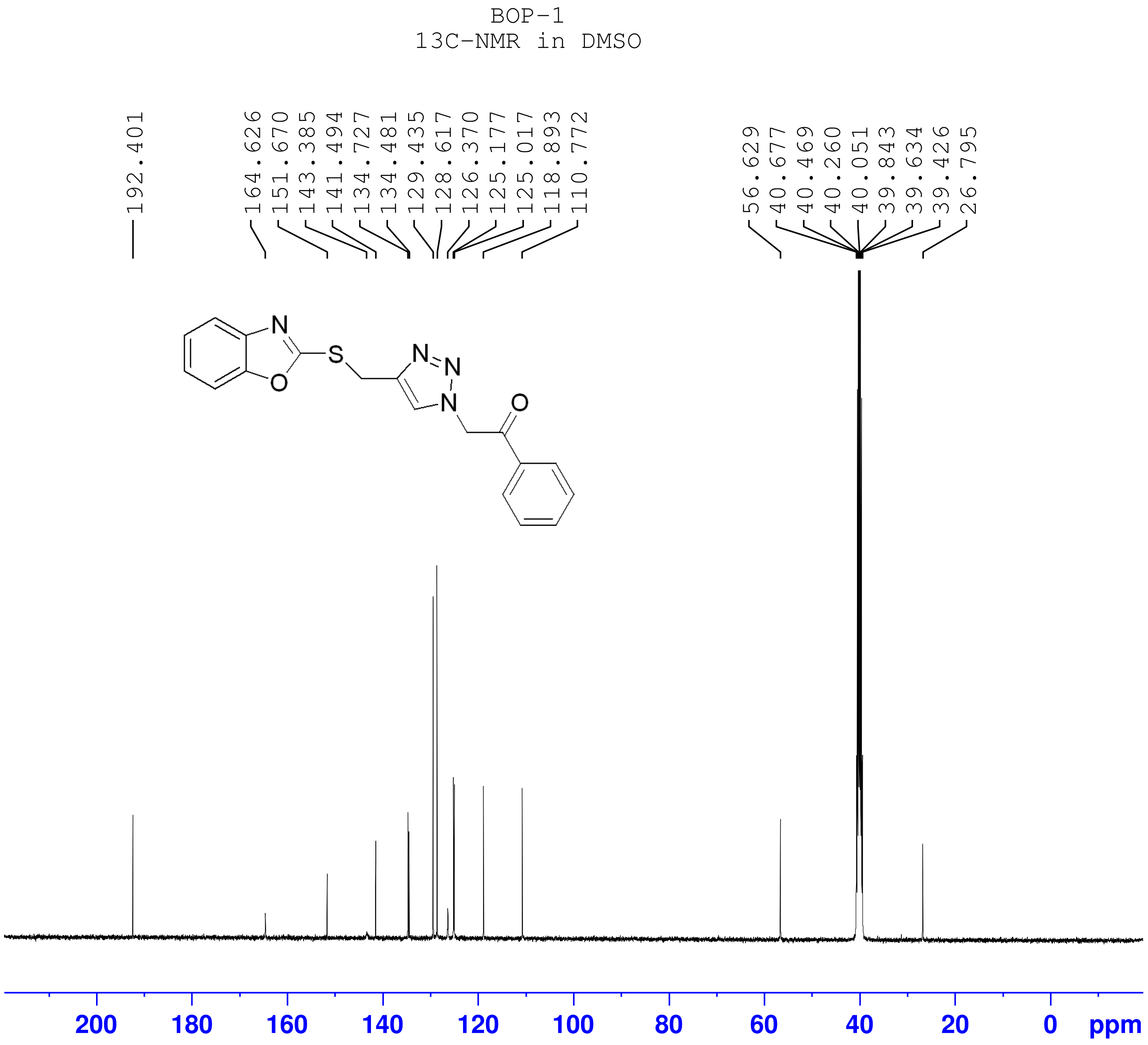

Current Data Parameters
NAME 23000787-Ph-BO
EXPNO 2
PROCNO 1

F2 - Acquisition Parameters
Date_ 20230801
Time 10.02 h
INSTRUM spect
PROBHD Z108618_0984 (
PULPROG zgpg30
TD 65536
SOLVENT DMSO
NS 1024
DS 4
SWH 24038.461 Hz
FIDRES 0.733596 Hz
AQ 1.3631488 sec
RG 2050
DW 20.800 usec
DE 6.50 usec
TE 297.2 K
D1 1.00000000 sec
D11 0.03000000 sec
TD0 1
SFO1 100.6680954 MHz
NUC1 13C
P0 3.33 usec
P1 10.00 usec
PLW1 51.02600098 W
SFO2 400.3116012 MHz
NUC2 1H
CPDPRG[2 waltz65
PCPD2 90.00 usec
PLW2 11.28999996 W
PLW12 0.27318001 W
PLW13 0.13741000 W

F2 - Processing parameters
SI 32768
SF 100.6580296 MHz
WDW EM
SSB 0
LB 1.00 Hz
GB 0
PC 1.40

^{13}C NMR spectrum of 2-(4-((benzo[d]oxazol-2-ylthio)methyl)-1H-1,2,3-triazol-1-yl)-1-phenylethan-1-one **3k (BOP-1).**

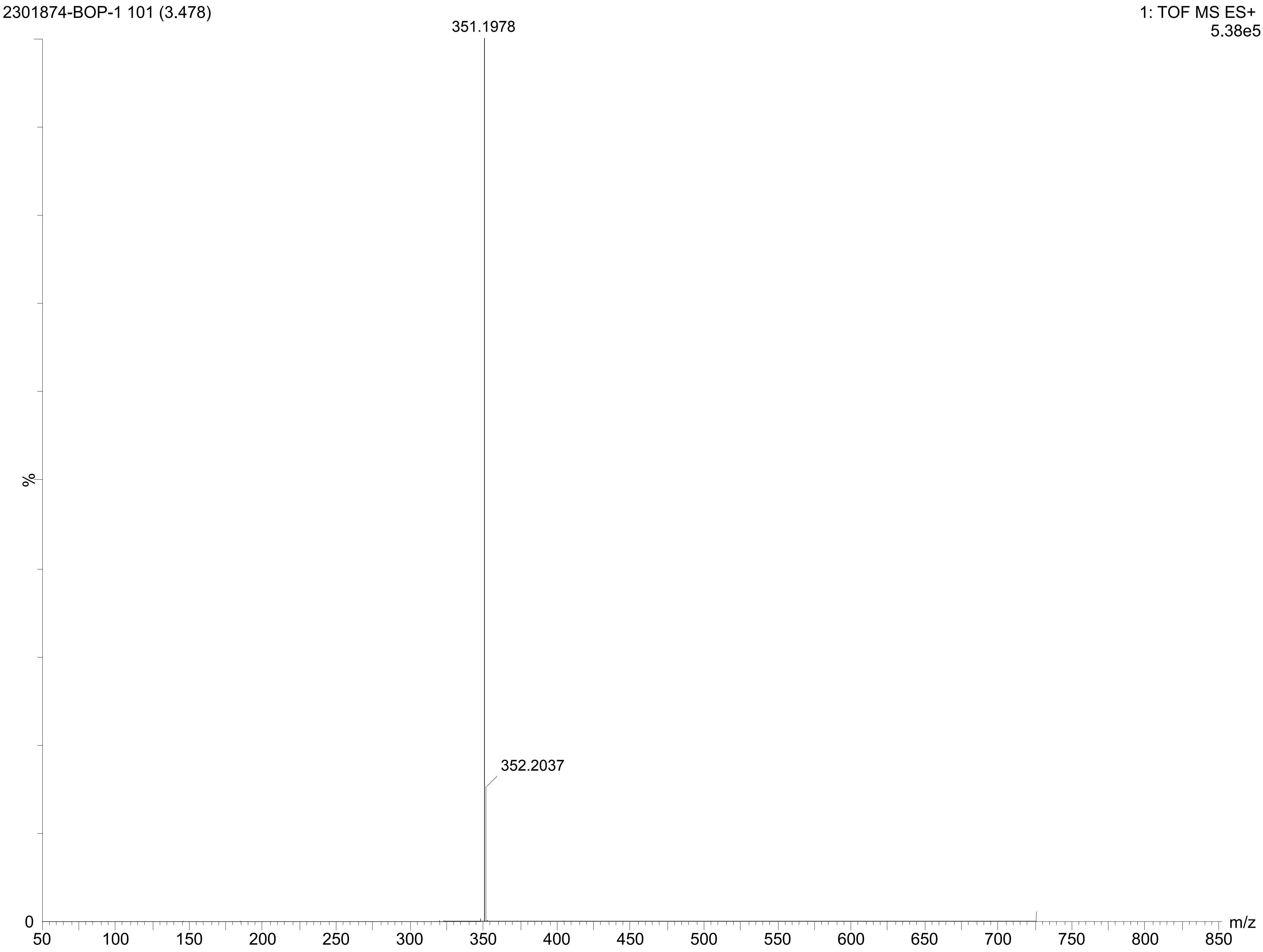

Mass spectrum of 2-(4-((benzo[d]oxazol-2-ylthio)methyl)-1H-1,2,3-triazol-1-yl)-1-phenylethan-1-one **3k (BOP-1)**.

COMPOUND 3l (BOP-2) SPECTRAL DATA

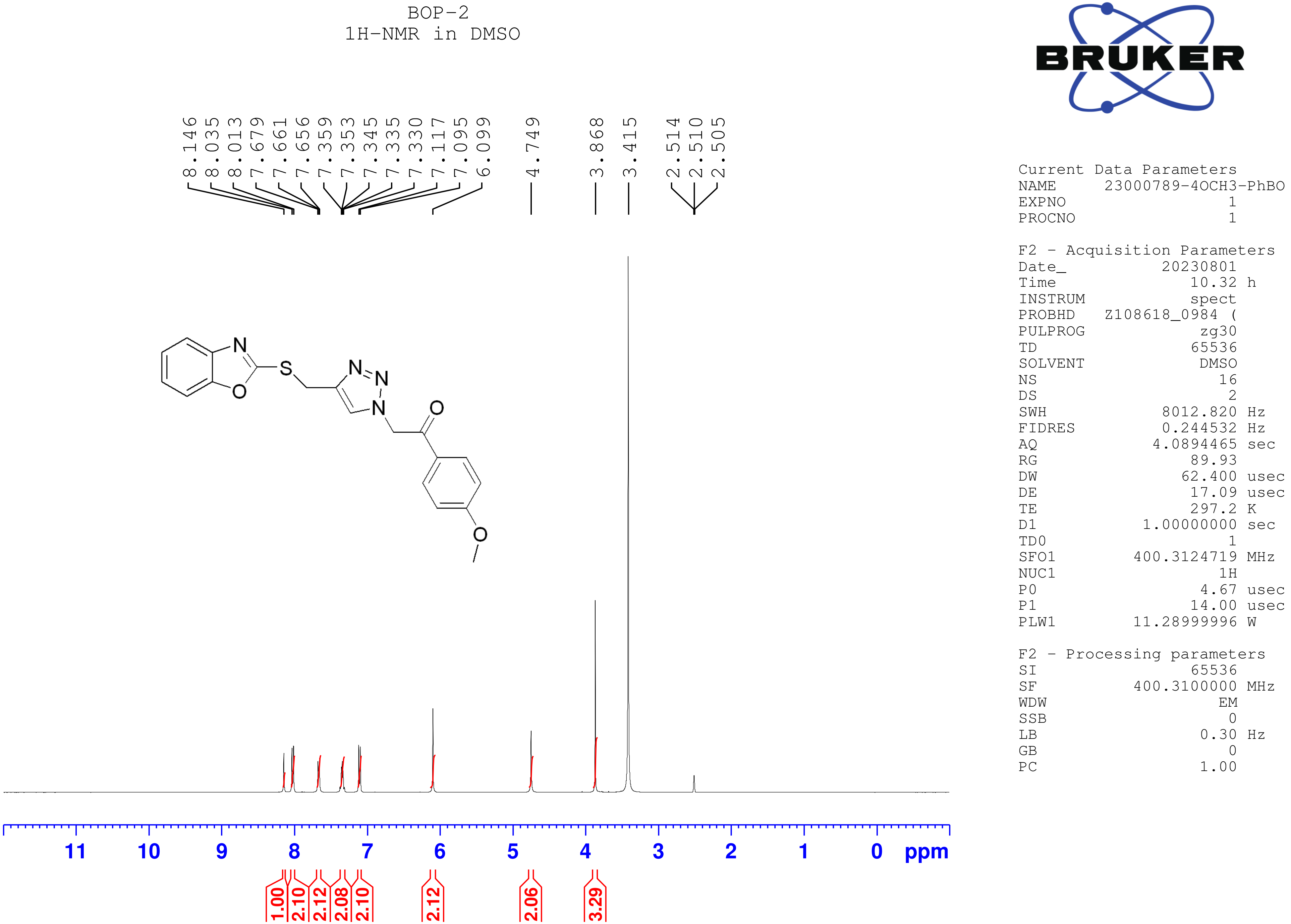

^{1}H NMR spectrum of 2-(4-((benzo[d]oxazol-2-ylthio)methyl)-1H-1,2,3-triazol-1-yl)-1-(4-methoxyphenyl)ethan-1-one **3l (BOP-2)**

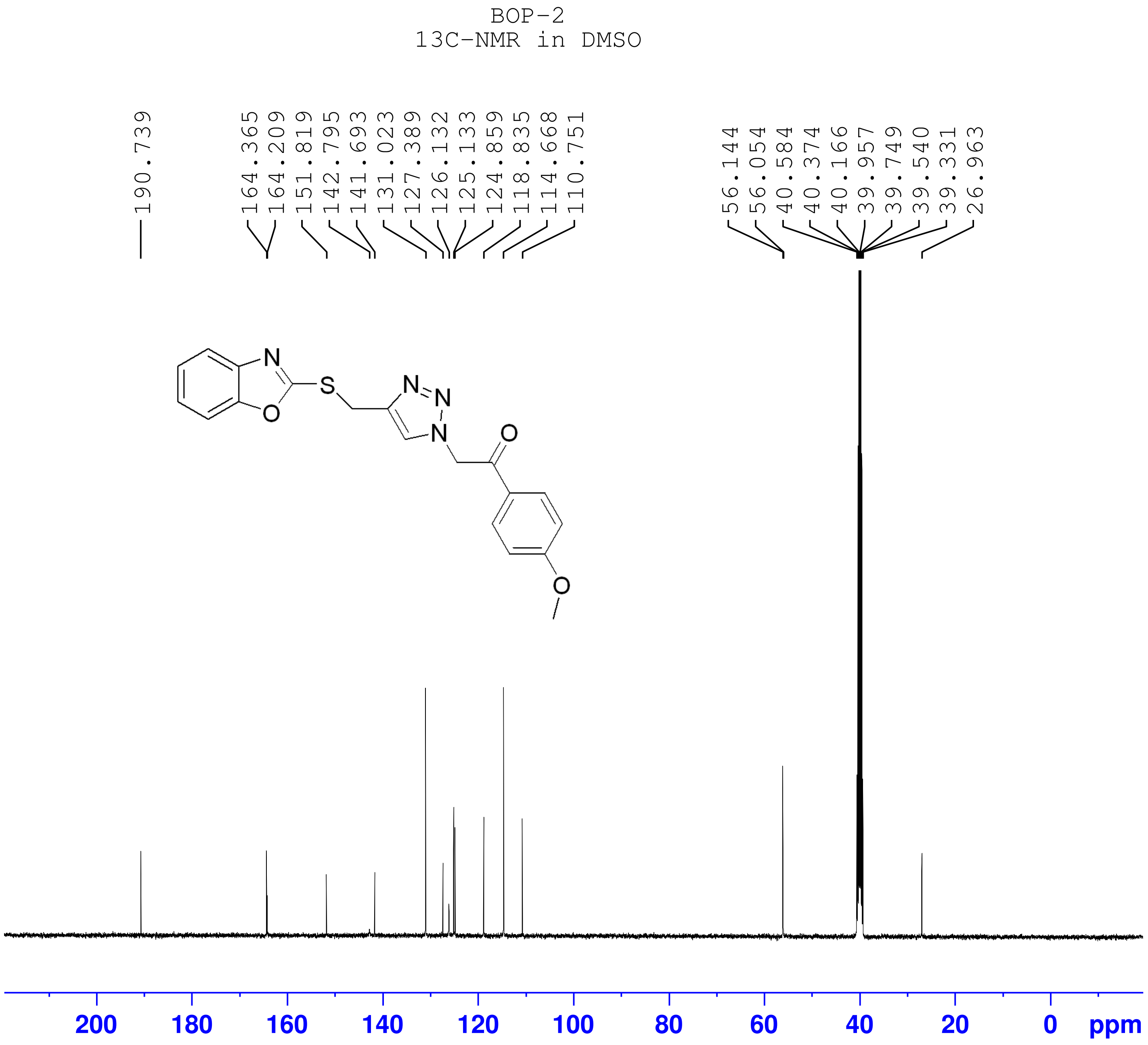

Current Data Parameters
NAME 23000789-4OCH3-PhBO
EXPNO 2
PROCNO 1

F2 - Acquisition Parameters
Date_ 20230801
Time 11.16 h
INSTRUM spect
PROBHD Z108618_0984 (
PULPROG zgpg30
TD 65536
SOLVENT DMSO
NS 1024
DS 4
SWH 24038.461 Hz
FIDRES 0.733596 Hz
AQ 1.3631488 sec
RG 2050
DW 20.800 usec
DE 6.50 usec
TE 298.5 K
D1 1.00000000 sec
D11 0.03000000 sec
TD0 1
SFO1 100.6680954 MHz
NUC1 13C
P0 3.33 usec
P1 10.00 usec
PLW1 51.02600098 W
SFO2 400.3116012 MHz
NUC2 1H
CPDPRG[2 waltz65
PCPD2 90.00 usec
PLW2 11.28999996 W
PLW12 0.27318001 W
PLW13 0.13741000 W

F2 - Processing parameters
SI 32768
SF 100.6580296 MHz
WDW EM
SSB 0
LB 1.00 Hz
GB 0
PC 1.40

^{13}C NMR spectrum of 2-(4-((benzo[d]oxazol-2-ylthio)methyl)-1H-1,2,3-triazol-1-yl)-1-(4-methoxyphenyl)ethan-1-one **3l (BOP-2)**

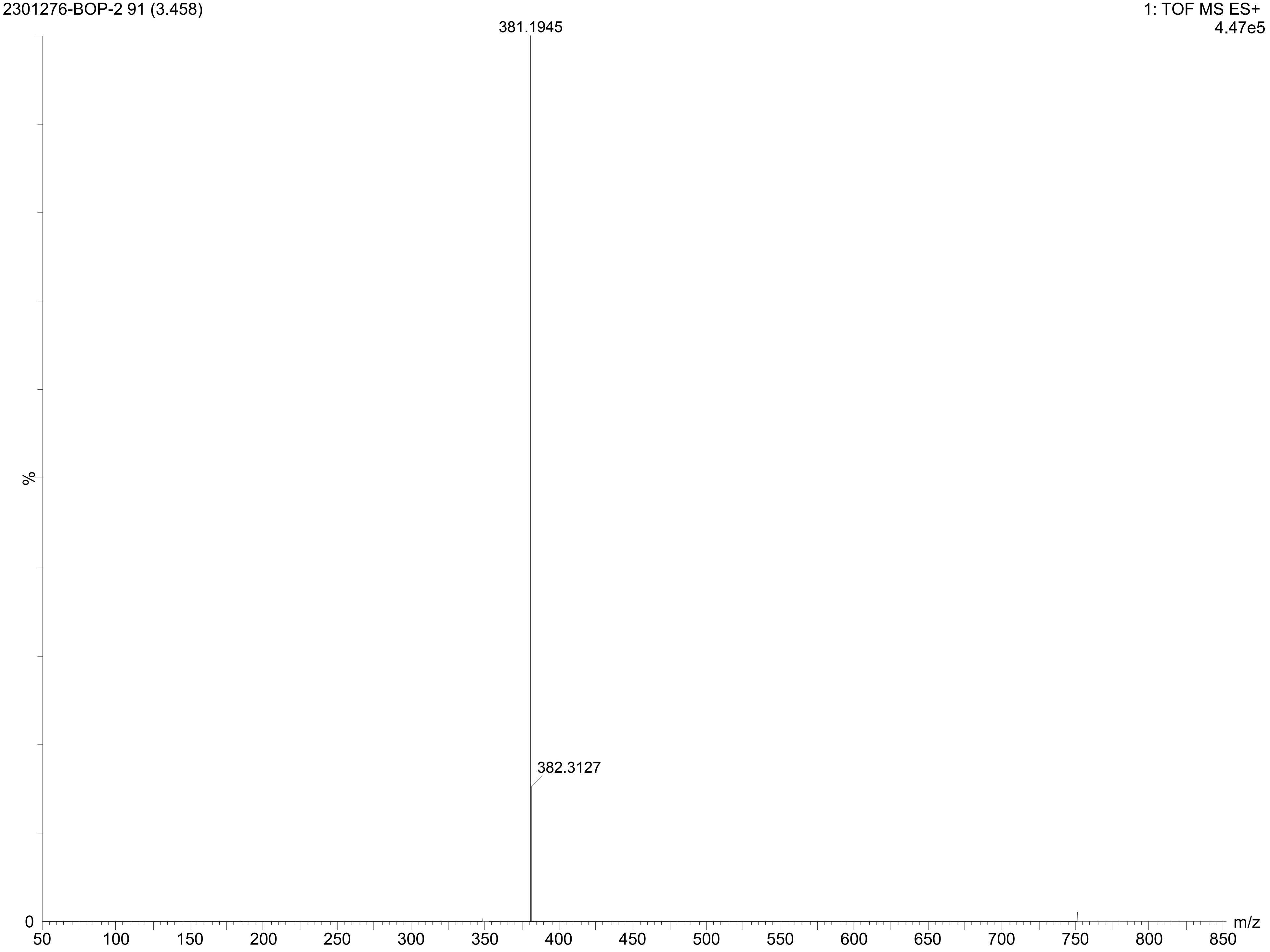

Mass spectrum of 2-(4-((benzo[d]oxazol-2-ylthio)methyl)-1H-1,2,3-triazol-1-yl)-1-(4-methoxyphenyl)ethan-1-one **3l (BOP-2).**

COMPOUND 3m (BOP-3) SPECTRAL DATA

BOP-3
1H-NMR in DMSO

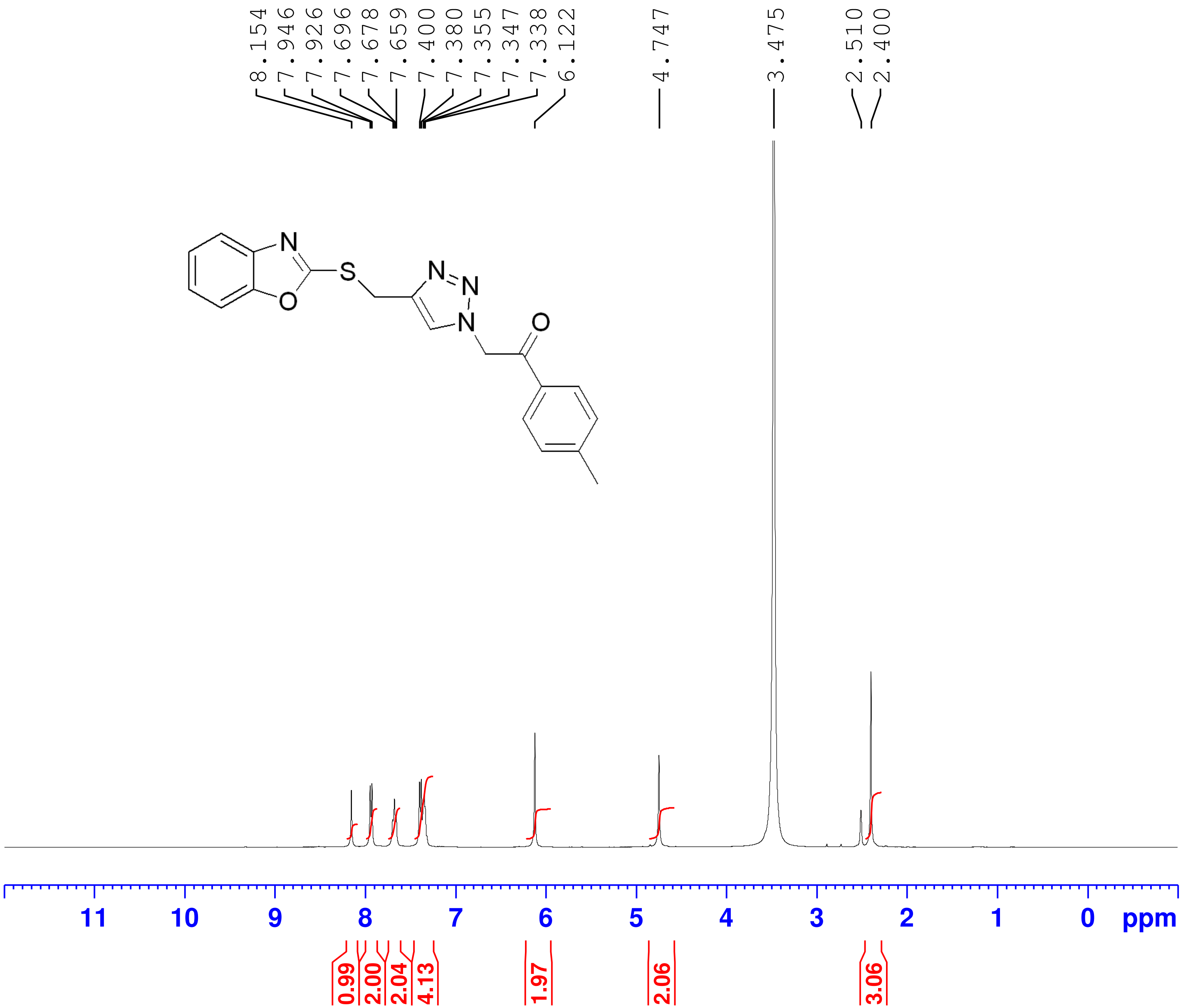

Current Data Parameters

NAME	23000622-4CH3-PhBo
EXPNO	1
PROCNO	1

F2 - Acquisition Parameters

Date_	20230628
Time	11.27 h
INSTRUM	spect
PROBHD	Z108618_0984 (
PULPROG	zg30
TD	65536
SOLVENT	DMSO
NS	32
DS	2
SWH	8012.820 Hz
FIDRES	0.244532 Hz
AQ	4.0894465 sec
RG	32.13
DW	62.400 usec
DE	17.09 usec
TE	297.5 K
D1	1.00000000 sec
TD0	1
SFO1	400.3124719 MHz
NUC1	1H
P0	4.67 usec
P1	14.00 usec
PLW1	11.28999996 W

F2 - Processing parameters

SI	65536
SF	400.3100000 MHz
WDW	EM
SSB	0
LB	0.30 Hz
GB	0
PC	1.00

^{1}H NMR spectrum of 2-(4-((benzo[d]oxazol-2-ylthio)methyl)-1H-1,2,3-triazol-1-yl)-1-(p-tolyl)ethan-1-one **3m (BOP-3).**

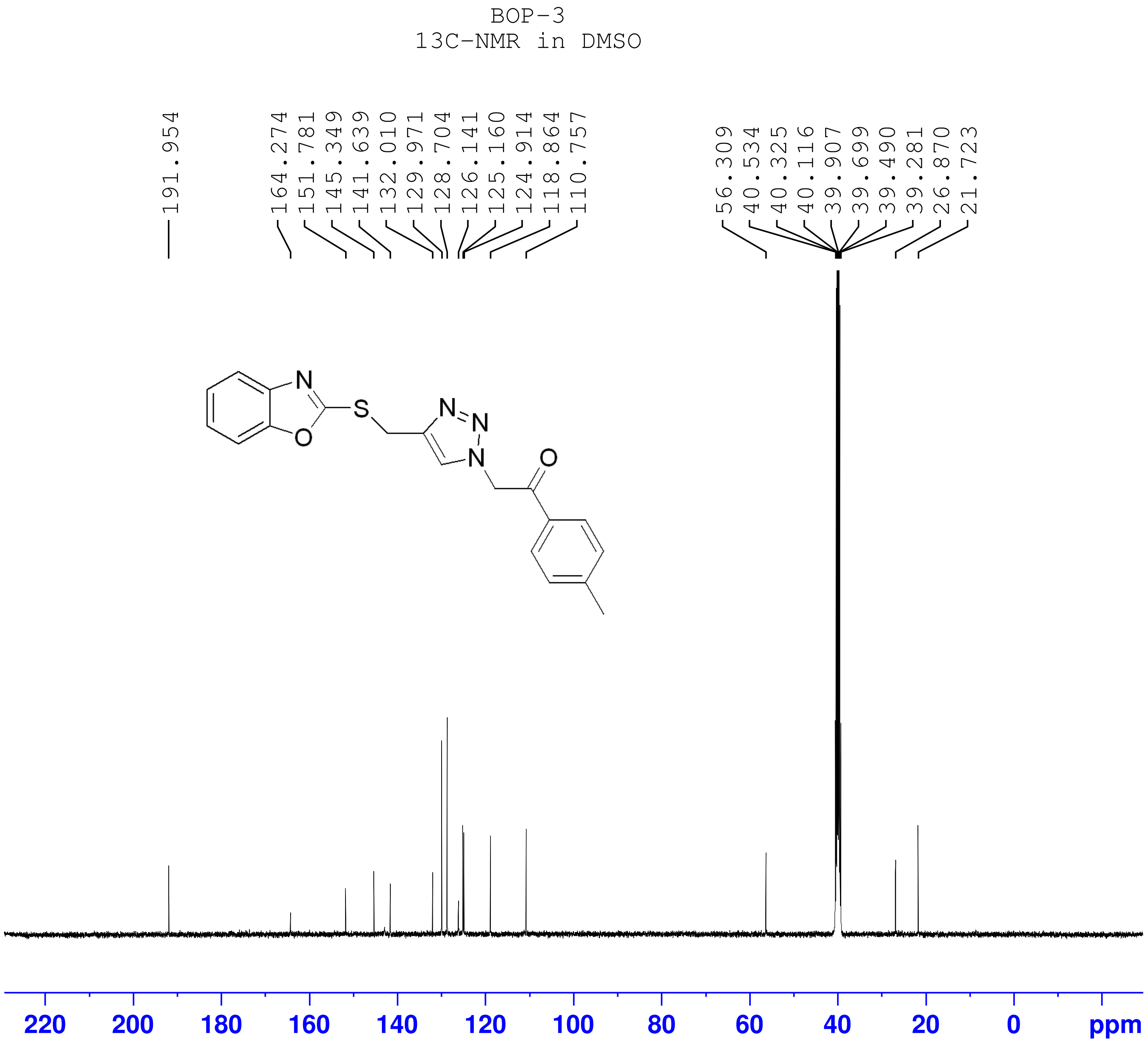

Current Data Parameters

NAME	23000622-4CH3-PhBo
EXPNO	2
PROCNO	1

F2 - Acquisition Parameters

Date_	20230628
Time	12.11 h
INSTRUM	spect
PROBHD	Z108618_0984 (
PULPROG	zgpg30
TD	65536
SOLVENT	DMSO
NS	1128
DS	4
SWH	26041.666 Hz
FIDRES	0.794729 Hz
AQ	1.2582912 sec
RG	202.84
DW	19.200 usec
DE	6.50 usec
TE	298.6 K
D1	1.00000000 sec
D11	0.03000000 sec
TD0	1
SFO1	100.6680954 MHz
NUC1	13C
P0	3.33 usec
P1	10.00 usec
PLW1	51.02600098 W
SFO2	400.3116012 MHz
NUC2	1H
CPDPRG[2	waltz65
PCPD2	90.00 usec
PLW2	11.28999996 W
PLW12	0.27318001 W
PLW13	0.13741000 W

F2 - Processing parameters

SI	32768
SF	100.6580296 MHz
WDW	EM
SSB	0
LB	1.00 Hz
GB	0
PC	1.40

^{13}C NMR spectrum of2-(4-((benzo[d]oxazol-2-ylthio)methyl)-1H-1,2,3-triazol-1-yl)-1-(p-tolyl)ethan-1-one **3m (BOP-3).**

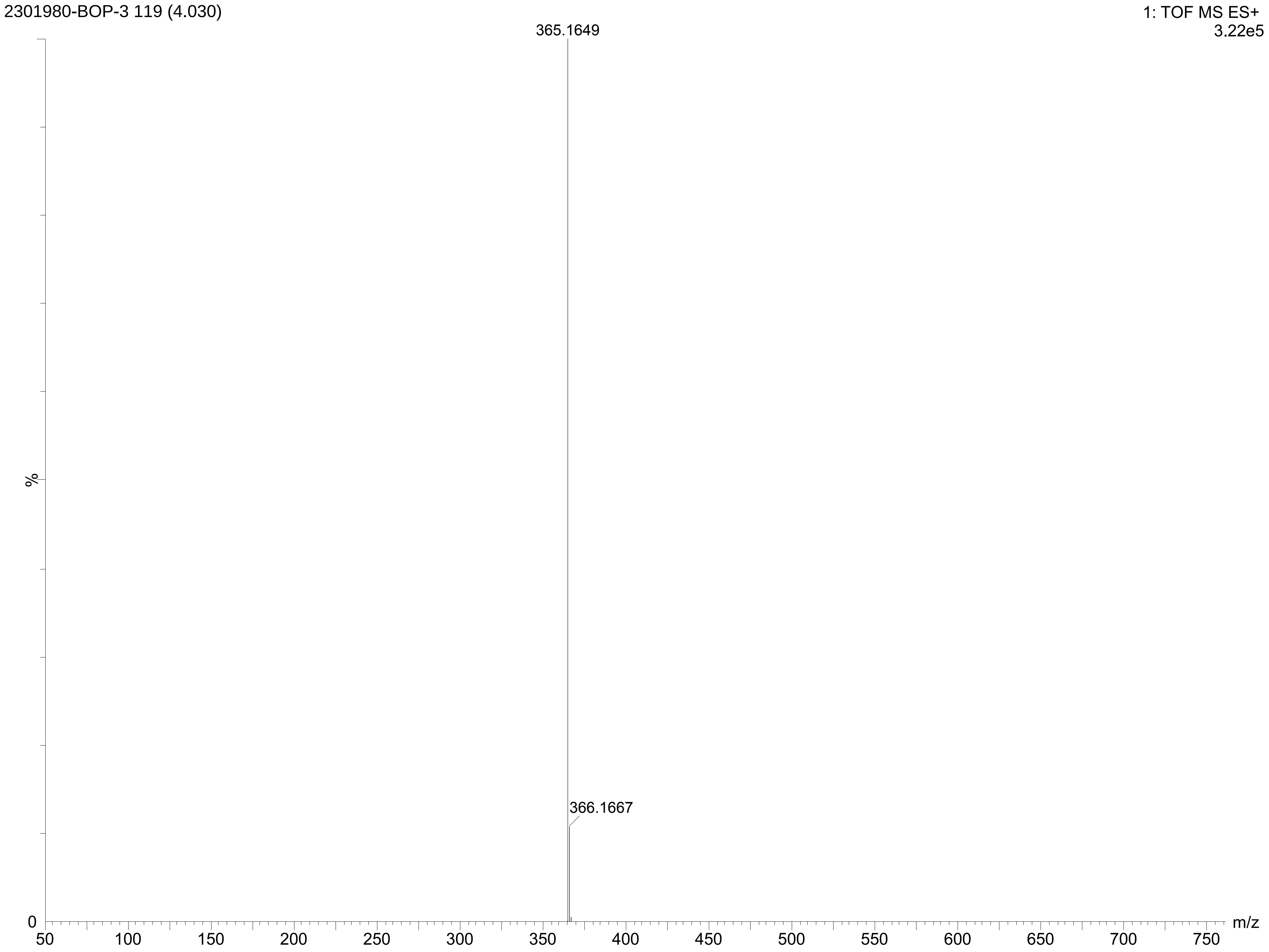

Mass spectrum of 2-(4-((benzo[d]oxazol-2-ylthio)methyl)-1H-1,2,3-triazol-1-yl)-1-(p-tolyl)ethan-1-one **3m (BOP-3)**

COMPOUND 3n (BOP-4) SPECTRAL DATA

BOP-4
1H-NMR in DMSO

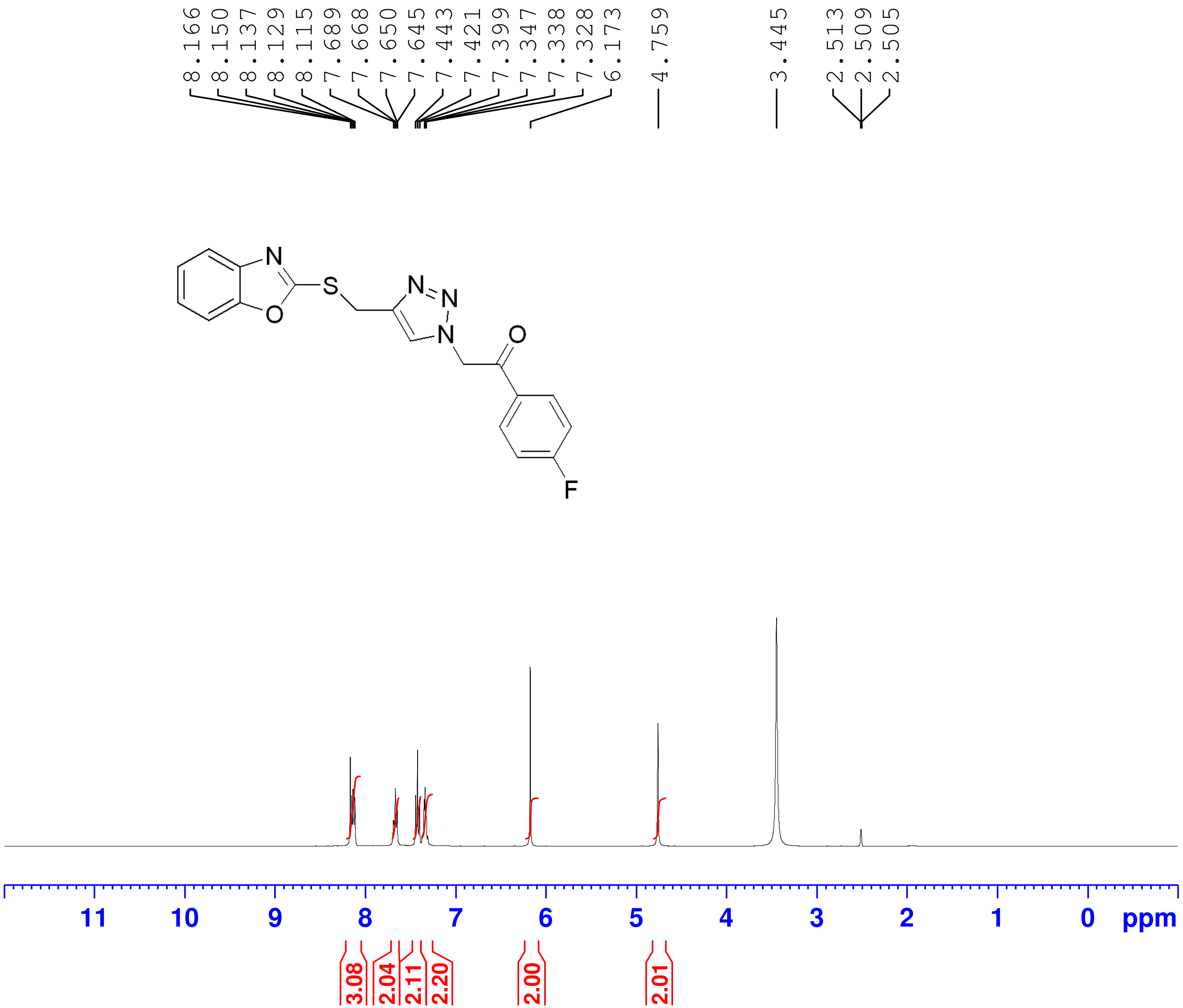

Current Data Parameters
NAME 23000788-4F-Ph-BO
EXPNO 1
PROCNO 1

F2 - Acquisition Parameters
Date_ 20230801
Time 10.06 h
INSTRUM spect
PROBHD Z108618_0984 (
PULPROG zg30
TD 65536
SOLVENT DMSO
NS 16
DS 2
SWH 8012.820 Hz
FIDRES 0.244532 Hz
AQ 4.0894465 sec
RG 73.11
DW 62.400 usec
DE 17.09 usec
TE 297.2 K
D1 1.00000000 sec
TD0 1
SFO1 400.3124719 MHz
NUC1 1H
P0 4.67 usec
P1 14.00 usec
PLW1 11.28999996 W

F2 - Processing parameters
SI 65536
SF 400.3100000 MHz
WDW EM
SSB 0
LB 0.30 Hz
GB 0
PC 1.00

^{1}H NMR spectrum of 2-(4-((benzo[d]oxazol-2-ylthio)methyl)-1H-1,2,3-triazol-1-yl)-1-(4-fluorophenyl)ethan-1-one **3n (BOP-4).**

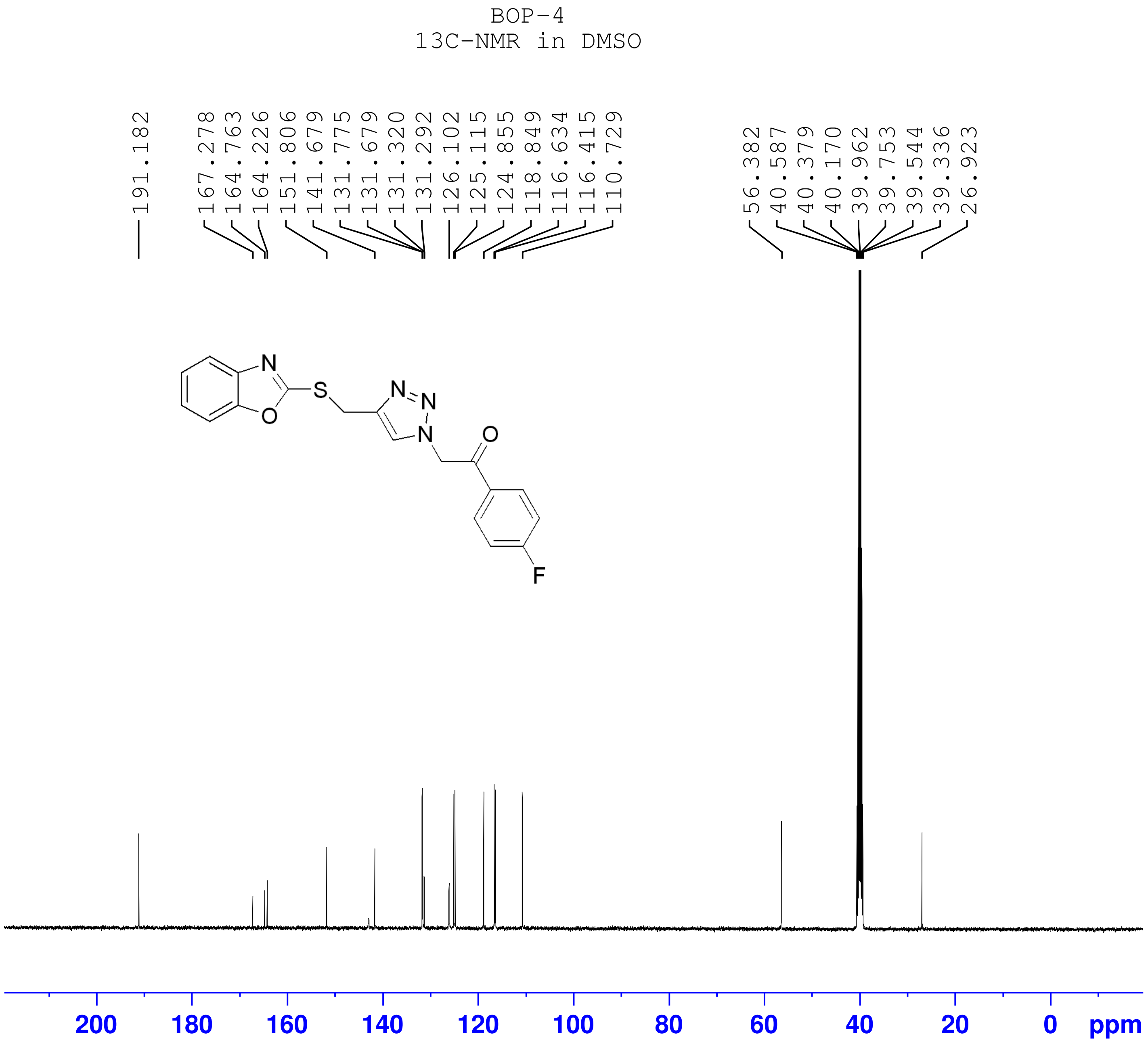

Current Data Parameters
NAME 23000788-4F-Ph-BO
EXPNO 2
PROCNO 1

F2 - Acquisition Parameters
Date_ 20230801
Time 10.28 h
INSTRUM spect
PROBHD Z108618_0984 (
PULPROG zgpg30
TD 65536
SOLVENT DMSO
NS 512
DS 4
SWH 24038.461 Hz
FIDRES 0.733596 Hz
AQ 1.3631488 sec
RG 2050
DW 20.800 usec
DE 6.50 usec
TE 298.2 K
D1 1.00000000 sec
D11 0.03000000 sec
TD0 1
SFO1 100.6680954 MHz
NUC1 13C
P0 3.33 usec
P1 10.00 usec
PLW1 51.02600098 W
SFO2 400.3116012 MHz
NUC2 1H
CPDPRG[2 waltz65
PCPD2 90.00 usec
PLW2 11.28999996 W
PLW12 0.27318001 W
PLW13 0.13741000 W

F2 - Processing parameters
SI 32768
SF 100.6580296 MHz
WDW EM
SSB 0
LB 1.00 Hz
GB 0
PC 1.40

^{13}C NMR spectrum of 2-(4-((benzo[d]oxazol-2-ylthio)methyl)-1H-1,2,3-triazol-1-yl)-1-(4-fluorophenyl)ethan-1-one **3n (BOP-4)**.

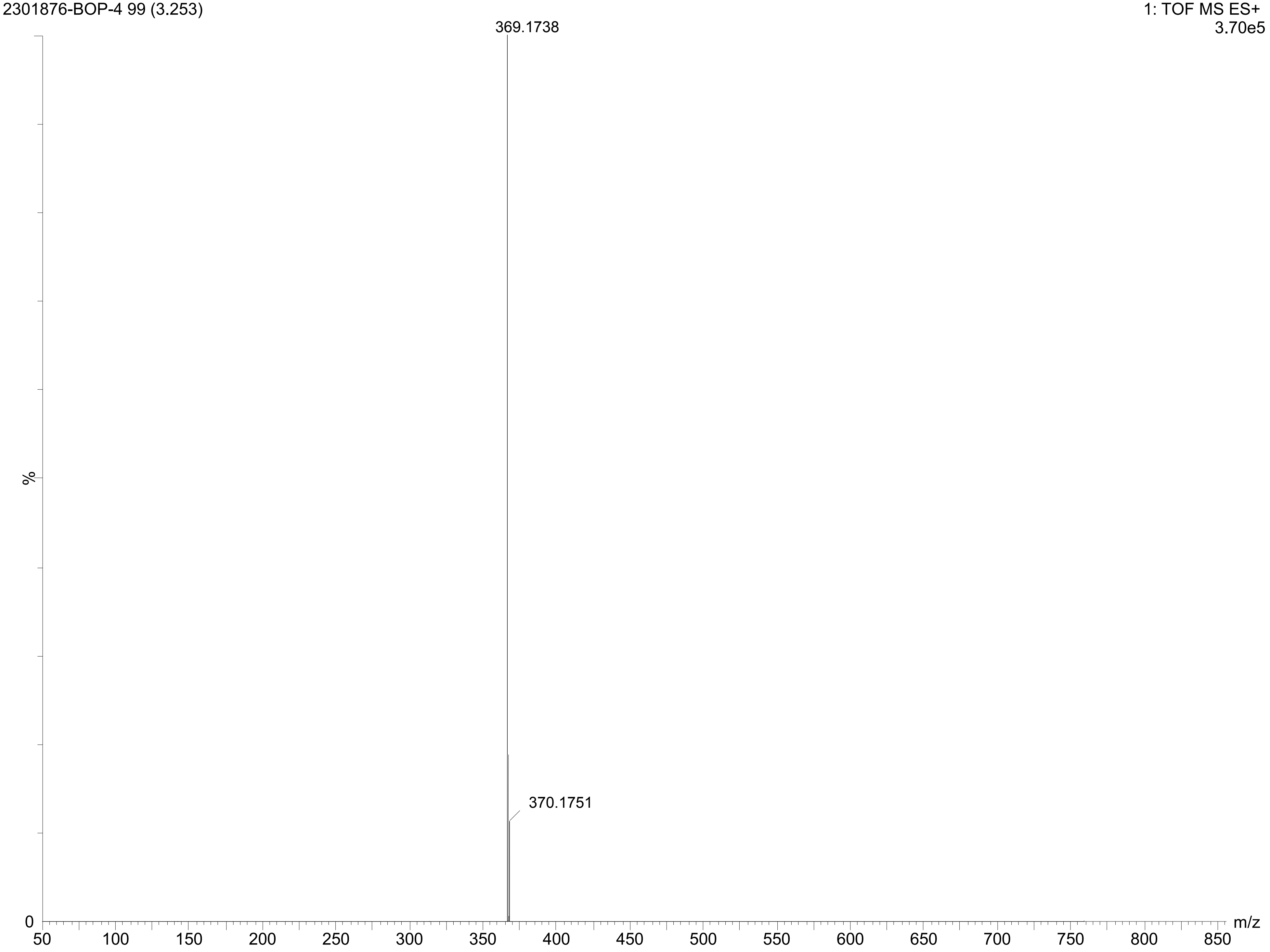

Mass spectrum of 2-(4-((benzo[d]oxazol-2-ylthio)methyl)-1H-1,2,3-triazol-1-yl)-1-(4-fluorophenyl)ethan-1-one **3n (BOP-4)**.

COMPOUND 3o (BOP-5) SPECTRAL DATA

BOP-5
1H-NMR in DMSO

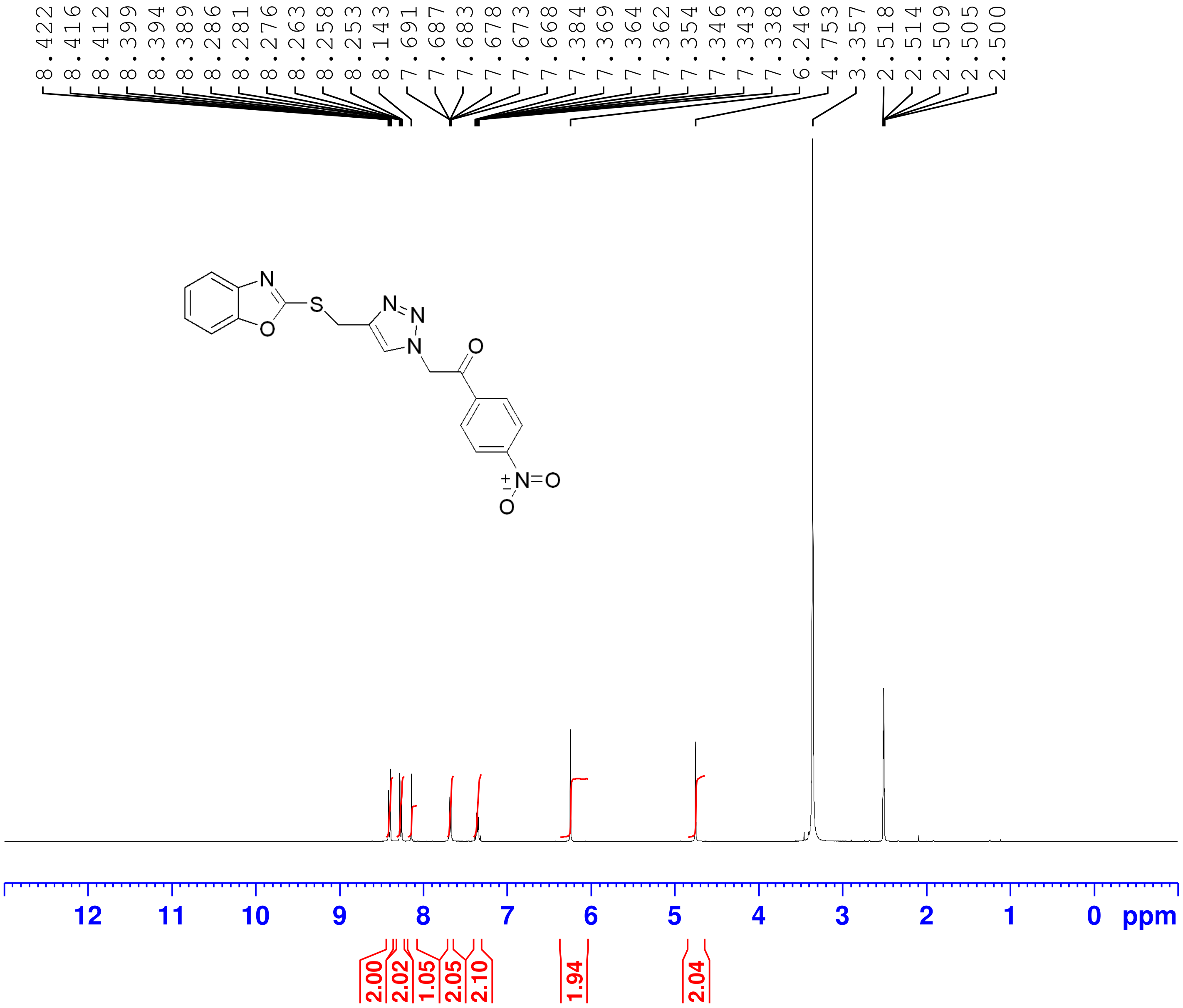

Current Data Parameters
NAME 23000514-4No2-PhBO
EXPNO 1
PROCNO 1

F2 - Acquisition Parameters
Date_ 20230520
Time 11.02 h
INSTRUM spect
PROBHD Z108618_0984 (
PULPROG zg30
TD 65536
SOLVENT DMSO
NS 32
DS 2
SWH 8012.820 Hz
FIDRES 0.244532 Hz
AQ 4.0894465 sec
RG 32.13
DW 62.400 usec
DE 17.09 usec
TE 297.5 K
D1 1.00000000 sec
TD0 1
SFO1 400.3124719 MHz
NUC1 1H
P0 4.67 usec
P1 14.00 usec
PLW1 11.28999996 W

F2 - Processing parameters
SI 65536
SF 400.3100000 MHz
WDW EM
SSB 0
LB 0.30 Hz
GB 0
PC 1.00

^{1}H NMR spectrum of 2-(4-((benzo[d]oxazol-2-ylthio)methyl)-1H-1,2,3-triazol-1-yl)-1-(4-nitrophenyl)ethan-1-one **3o (BOP-5).**

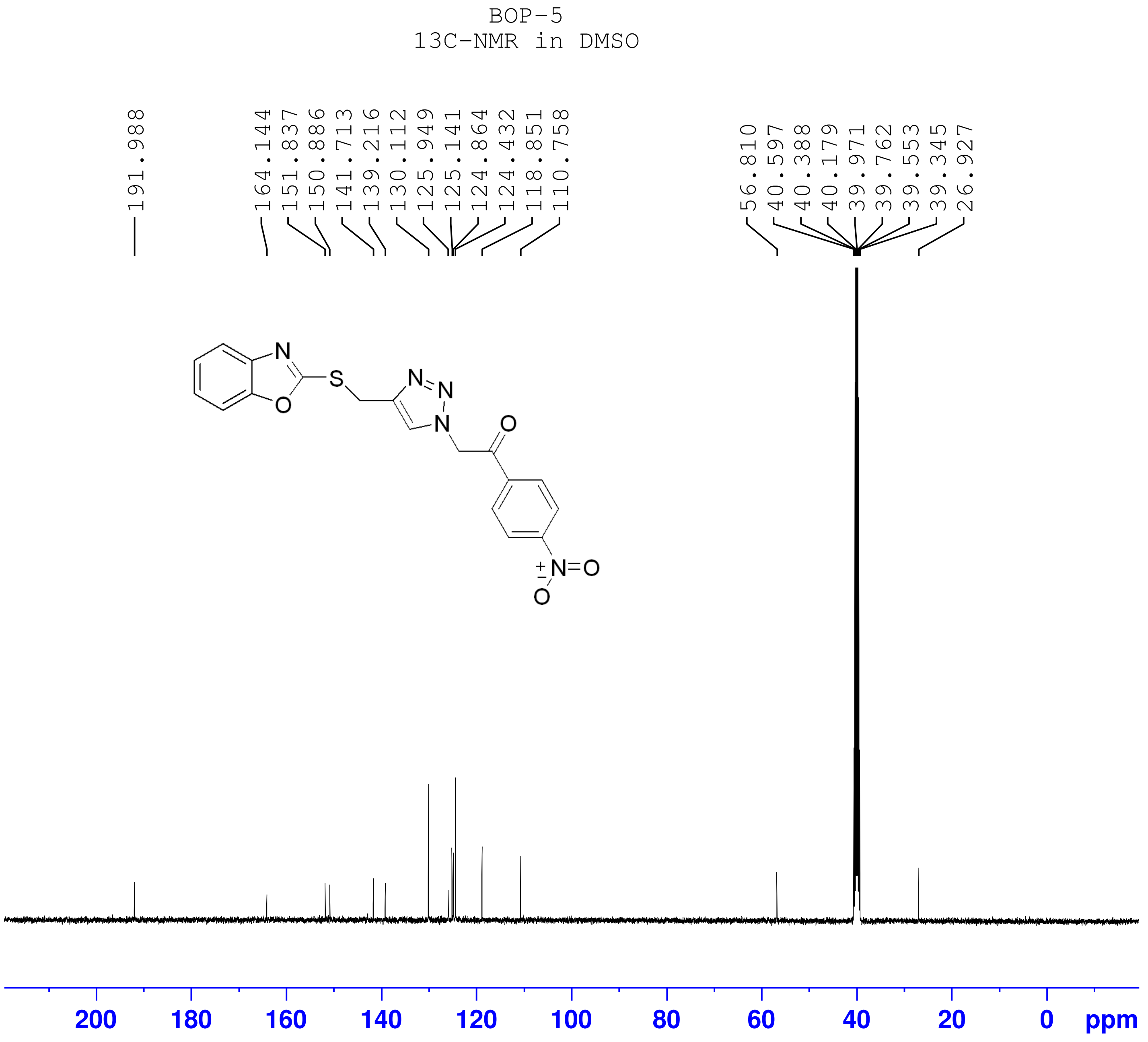

Current Data Parameters

NAME	23000514-4No2-PhBO
EXPNO	2
PROCNO	1

F2 - Acquisition Parameters

Date_	20230522
Time	12.42 h
INSTRUM	spect
PROBHD	Z108618_0984 (
PULPROG	zgpg30
TD	65536
SOLVENT	DMSO
NS	512
DS	4
SWH	24038.461 Hz
FIDRES	0.733596 Hz
AQ	1.3631488 sec
RG	202.84
DW	20.800 usec
DE	6.50 usec
TE	297.6 K
D1	1.00000000 sec
D11	0.03000000 sec
TD0	1
SFO1	100.6680954 MHz
NUC1	13C
P0	3.33 usec
P1	10.00 usec
PLW1	51.02600098 W
SFO2	400.3116012 MHz
NUC2	1H
CPDPRG[2	waltz65
PCPD2	90.00 usec
PLW2	11.28999996 W
PLW12	0.27318001 W
PLW13	0.13741000 W

F2 - Processing parameters

SI	32768
SF	100.6580296 MHz
WDW	EM
SSB	0
LB	1.00 Hz
GB	0
PC	1.40

^{13}C NMR spectrum of2-(4-((benzo[d]oxazol-2-ylthio)methyl)-1H-1,2,3-triazol-1-yl)-1-(4-nitrophenyl)ethan-1-one **3o (BOP-5).**

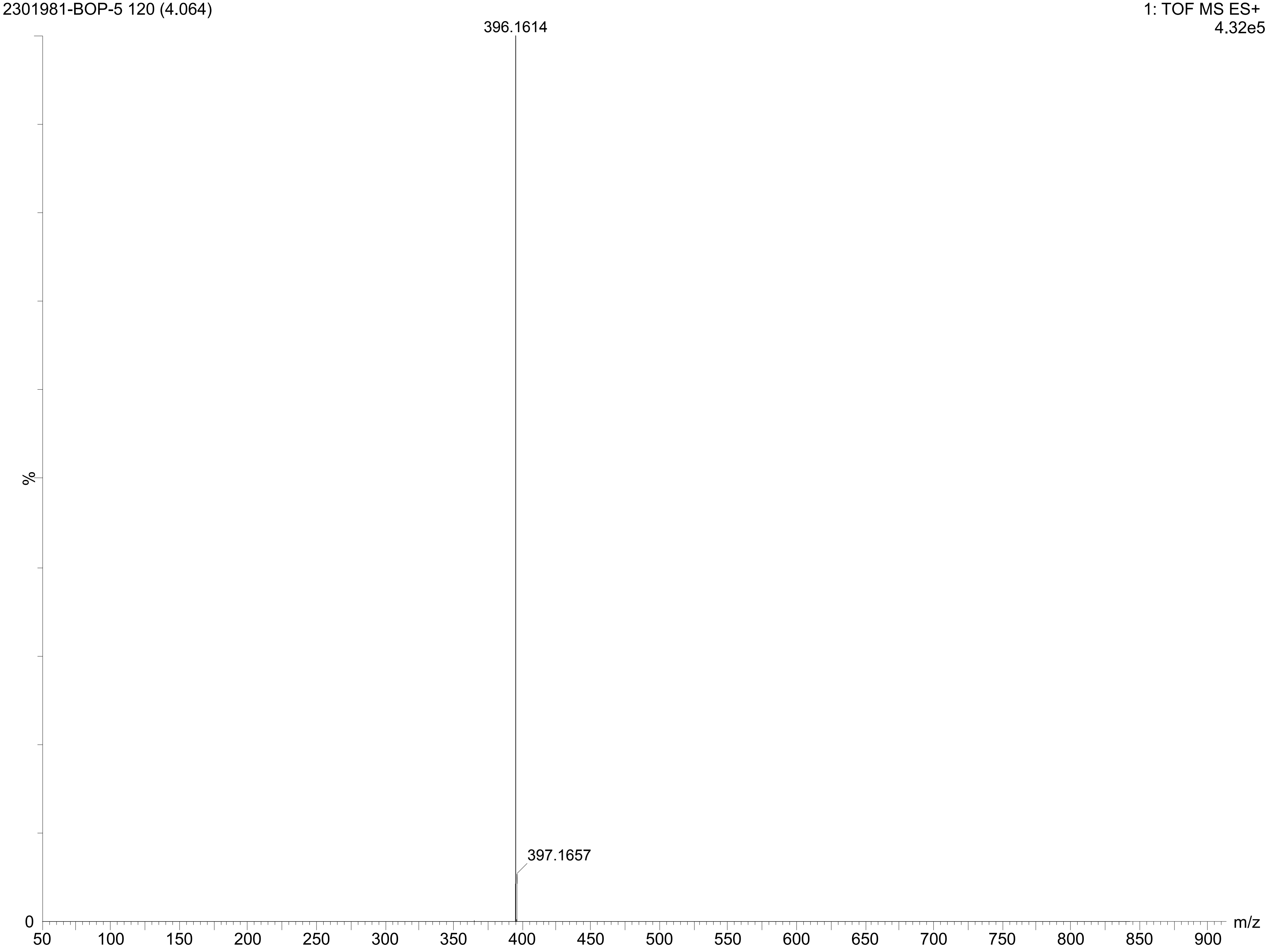

Mass spectrum of 2-(4-((benzo[d]oxazol-2-ylthio)methyl)-1H-1,2,3-triazol-1-yl)-1-(4-nitrophenyl)ethan-1-one **3o (BOP-5).**

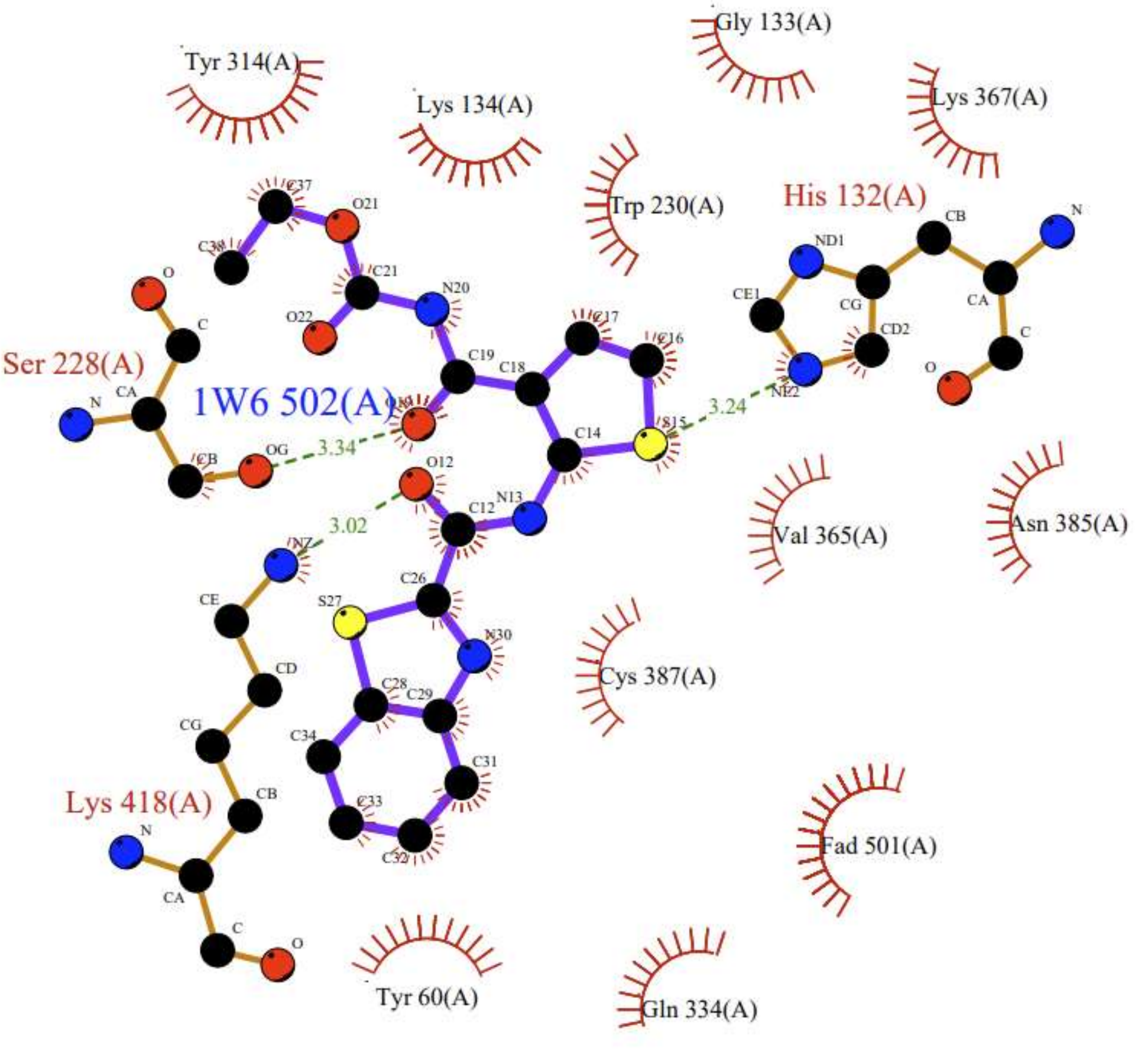

Fig. S46. DprE1 protein binding sheet for TCA-1

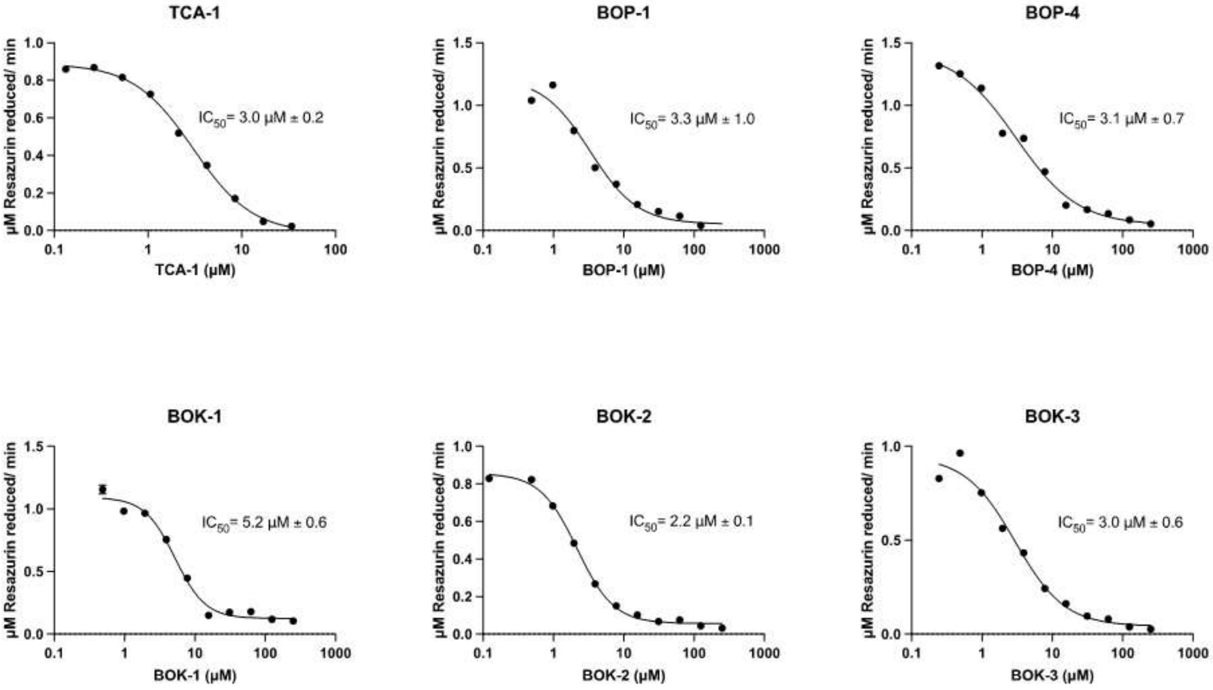

Fig. S47. Concentration response graph of DprE1 inhibition fluorometric assay

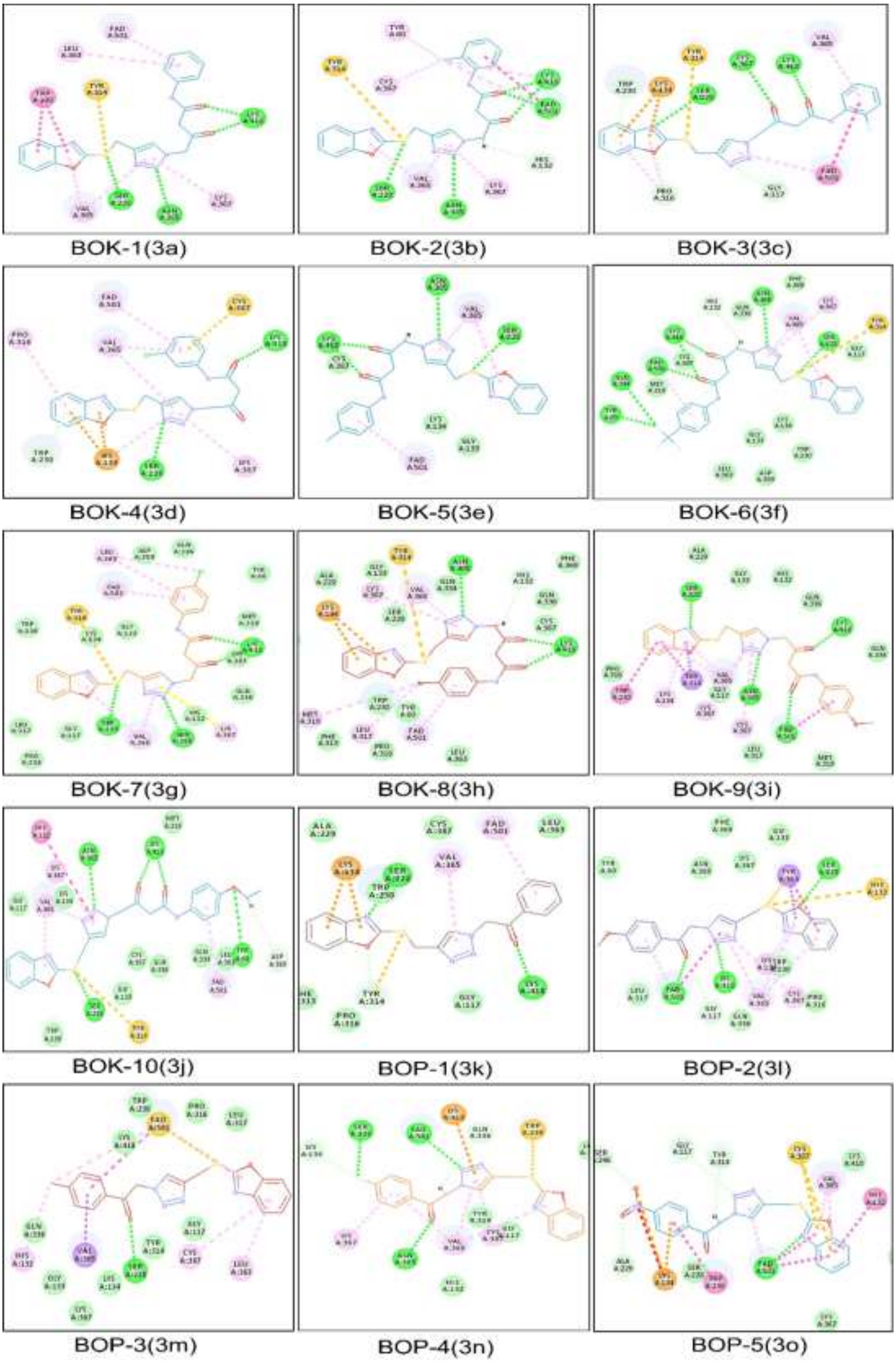

Fig. S48. 2D molecular docking interaction images of the compounds

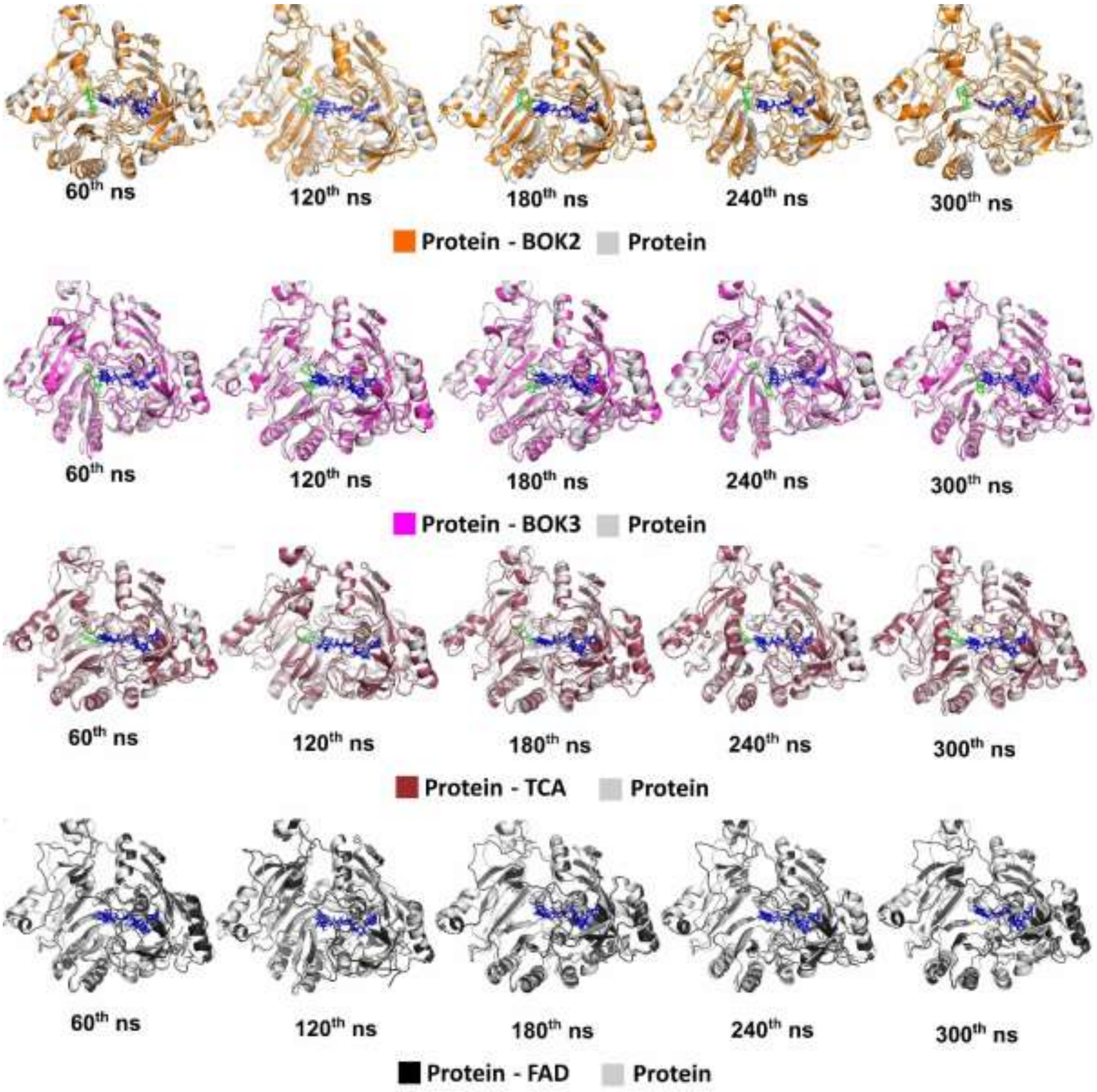

Fig. S49 MD trajectory analysis of superimposed structures of ligand-free protein and ligand-bound protein at different time intervals.

Table. S1. Molecular Docking Scores for Synthesised Compounds with Different substitutions.

S.No.	Compound Code	Substitution	Position of substitution	(-) CDocker Energy	(-) CDocker interaction energy
1.	BOK-4	Chloro	meta	34.54	52.72
2.	BOK-5	Methyl	para	37.58	51.58
3.	BOK-6	TrimethylFluoro	para	37.49	54.15
4.	BOK-7	Chloro	para	36.89	52.36
5.	BOK-8	Bromo	para	35.30	56.69
6.	BOK-9	Methoxy	para	33.81	52.73
7.	BOK-10	Ethoxy	para	36.97	53.26
8.	BOP-2	Methoxy	para	23.74	44.71
9.	BOP-3	Methyl	para	25.71	43.67
10.	BOP-5	Nitro	para	22.28	45.95

Table. S2 Assessment of ADMET Characteristics and Drug-likeness in Newly Synthesised

S.No.	Ligand	MW	RB	HBA	HBD	MR	TPSA	ilogP	ESOL LogS	BBB	log Kp	LV	BS	PAINS alerts
1.	BOK-1	407.45	9	6	1	108.71	128.21	2.07	-4.37	No	-6.44	0	0.55	0
2.	BOK-2	421.47	9	6	1	113.67	128.21	2.7	-4.67	No	-6.26	0	0.55	0
3.	BOK-3	425.44	9	7	1	108.66	128.21	2.54	-4.53	No	-6.47	0	0.55	0
4.	BOK-4	441.89	9	6	1	113.72	128.21	2.11	-4.96	No	-6.33	0	0.55	0
5.	BOK-5	421.47	9	6	1	113.67	128.21	2.32	-4.67	No	-6.06	0	0.55	0
6.	BOK-6	475.44	10	9	1	113.71	128.21	2.34	-5.22	No	-6.60	0	0.55	0
7.	BOK-7	441.89	9	6	1	113.72	128.21	2.28	-4.96	No	-6.33	0	0.55	0
8.	BOK-8	486.34	9	6	1	116.41	128.21	3.20	-5.27	No	-6.39	0	0.55	0
9.	BOK-9	437.47	10	7	1	115.20	137.44	3.29	-4.44	No	-5.84	0	0.55	0
10.	BOK-10	451.50	11	7	1	120.01	137.44	2.88	-4.68	No	-7.27	0	0.55	0
11.	BOP-1	350.39	6	5	1	94.77	99.11	2.74	-4.36	No	-6.01	0	0.55	0
12.	BOP-2	380.42	7	6	0	101.27	108.34	3.08	-4.42	No	-5.34	0	0.55	0
13.	BOP-3	364.42	6	5	0	99.74	99.11	3.02	-4.65	No	-5.56	0	0.55	0
14.	BOP-4	368.38	6	6	1	94.73	99.11	2.7	-4.51	No	-6.05	0	0.55	0
15.	BOP-5	395.39	7	7	0	103.60	144.93	2.31	-4.41	No	-5.76	0	0.55	0
16.	TCA-1	375.42	8	5	2	96	153.87	1.81	-4.54	No	-5.79	0	0.55	0

Compounds[a] in comparison to a literature-reported molecule (TCA-1).

[a]MW = molecular weight, RB = Rotatable bonds, HBA = Hydrogen bond Acceptor, HBD = Hydrogen bond donor, MR = Molecular Refractivity, TPSA = topological polar surface area, ilogP = Octanol-water partition coefficient, ESOL logS = Aqueous Solubility, BBB = blood brain barrier, log kP = Skin Permeation, LV = Lipinski's violation, BS = Bioavailability Score, and PAINS alerts = Pan-Assay Interference.
